# Floristic and Vegetation Changes on a Small Mediterranean Island over the Last Century

**DOI:** 10.3390/plants10040680

**Published:** 2021-04-01

**Authors:** Saverio Sciandrello, Salvatore Cambria, Gianpietro Giusso del Galdo, Riccardo Guarino, Pietro Minissale, Salvatore Pasta, Gianmarco Tavilla, Antonia Cristaudo

**Affiliations:** 1 Department of Biological, Geological and Environmental Sciences, University of Catania, via A. Longo 19, 95125 Catania, Italy; cambria_salvatore@yahoo.it (S.C.); g.giusso@unict.it (G.G.d.G.); p.minissale@unict.it (P.M.); gianmarco.tavilla@phd.unict.it (G.T.); acristau@unict.it (A.C.); 2 Department STEBICEF, University of Palermo, via Archirafi 38, 90123 Palermo, Italy; riccardo.guarino@unipa.it; 3 Institute of Biosciences and BioResources (IBBR), National Research Council (CNR), Unit of Palermo, Corso Calatafimi 414, 90129 Palermo, Italy; salvatore.pasta@ibbr.cnr.it

**Keywords:** landscape dynamics, turnover, nature conservation, diachronic analysis, vegetation, Mediterranean islets

## Abstract

A synthetic and updated overview about the vascular flora and vegetation of the Island of Capo Passero (SE-Sicily) is provided. These data issue from two series of field surveys—the first carried out between 1997 and 2000, and the second between 2005 and 2019 and mostly focused on refining and implementing vegetation data. The current islet’s flora consists of 269 *taxa*, of which 149 (58%) are annual plants. The Mediterranean species are largely prevailing, 108 (40%) of which have a strictly Mediterranean biogeographical status. The comparison with a species list published in 1919 and updated in 1957 suggest that, despite the overall prevalence of anemochorous taxa, the vertebrate fauna represents an important vector for the plant colonization of the island, while the immigration of myrmechocorous taxa does not compensate the extinction rate. As many as 202 phytosociological relevés, 191 of which issue from original recent field surveys, enabled identifying 12 different plant communities. The comparison with a vegetation map published in 1965 suggests a strong reduction in dune habitats (2120 and 2210 according to EU ‘Habitats’ Directive 92/43), as well as a deep disruption in the succession typical of the local psammophilous vegetation series. In order to preserve rare, endangered and protected plant species (such as *Aeluropus lagopoides*, *Cichorium spinosum*, *Limonium hyblaeum*, *L. syracusanum*, *Poterium spinosum*, *Senecio pygmaeus* and *Spergularia heldreichii*) and to stop the ongoing habitat degradation, urgent and effective conservation measures should be adopted for this tiny, yet precious islet.

## 1. Introduction

Although they represent a small part of the emerged lands, islands host a remarkable portion of the global biological richness [1]. Indeed, the isolation of these lands and their ecosystems has not only favoured the processes of evolutionary divergence and endemism, but also offered refuge to organisms that are threatened or have disappeared elsewhere.

Additionally, the small uninhabited islets offer an exceptional research field for life scientists because they represent both conservative and extremely simplified contexts [2,3,4,5,6,7,8,9]. As for the small circum-Sicilian islets, they represent sites of high conservation value for the occurrence of several endemic or rare vascular plant species [10,11]. In the past, many of them drove the interest of botanists, which have mainly investigated their vascular floras [12,13,14,15,16,17,18,19,20,21,22,23,24,25,26,27].

Among the circum-Sicilian islets, the Island of Capo Passero (SW-Sicily) stands out for having a prominent interest because, thanks to its easy accessibility, it has been targeted by numerous botanists. Its vascular flora was investigated for the first time during spring 1664 by the English botanist John Ray, who recorded more than 60 plants. Even if incomplete, Ray’s list probably represents the first inventory of a small Mediterranean island and provides interesting information on the plants growing there more than 350 years ago [28,29].

A far more complete plant species list, issuing from two visits carried out on the islet (1909 and 1917), was published long time after by Albo [30], with some additions 40 years later [31]. The Island of Capo Passero has been attractive to botanists not only for its flora but also for its landscape peculiarities. For instance, Albo [30] emphasized the remarkable integrity and extent of the local dwarf palm (*Chamaerops humilis* L.) community, defined in the same years as “the most beautiful dwarf-palm shrubland in Italy” [32]. The first vegetation surveys were carried out on the islet by Pirola [33,34]. During the following decades, many species new to Island of Capo Passero have been reported by Galletti [35] and by Camatta et al. [36].

In the last 25 years, the authors of the present contribution carried out two series of field surveys. The first one (from 1997 to 2000) enabled actualizing local flora and vegetation data, the second (from 2005 to 2019) was mostly focused on refining and implementing the vegetation dataset. With the exception of some information already published by Cristaudo and Maugeri [37] and Cristaudo and Margani [38], the data issuing from both campaigns are here presented for the first time. Main objective of this contribution are: (1) to provide an updated list of the local vascular flora, (2) to present a comprehensive syntaxonomic framework of the local plant communities, and (3) to perform a diachronic analysis of the flora and vegetation changes occurred during last 60 years and provide an explanation for them.

## 2. Results and Discussion

### 2.1. The Vascular Flora: Traits Analysis and Taxa of Outstanding Interest

The vascular plant species recorded from the Island of Capo Passero during the last century and their related traits are listed in alphabetical order in Appendix A.

**Figure 1:**
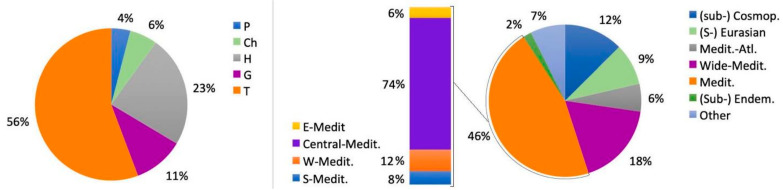
Life form (**left**) and biogeographical status (**right**) of the *taxa* recorded from the Island of Capo Passero in our survey (see Appendix A). Life form abbreviations: P, phanerophytes; Ch, chamaephytes; H hemicryptophytes; G, geophytes; T, therophytes.

Our field surveys enabled confirming the occurrence of 269 *taxa* belonging to 55 families. The families represented by more than 10 *taxa* were the following: Asteraceae (42 *taxa*), Poaceae (38 *taxa*), Fabaceae (29 *taxa*), Caryophyllaceae (14 *taxa*) and Apiaceae (11 *taxa*). Therophytes were the prevalent life form (149 *taxa*, i.e., 56% of the whole flora), followed by hemicryptophytes (64; 23%) and geophytes (29; 11%), while the percentage of chamaephytes (16; 6%) and (nano-)phanerophytes (11; 4%) was very low (Figure 1).

The Mediterranean chorotype was largely prevailing (124 *taxa*; 46%). Relevant was also the presence of the wide-Mediterranean (48; 18%) and (sub-)cosmopolitan *taxa* (34; 12%). The endemic component included two *taxa* restricted to SE-Sicily, *Limonium hyblaeum* and *L. syracusanum*, one restricted to SE-Sicily, Lampedusa and the Maltese Islands, *Senecio pygmaeus*, and two restricted to Sicily and southern Italy, *Crocus longiflorus* and *Echium italicum* subsp. *siculum*. The alien component, lumped into the category "other", is really negligible, being represented by five species which do not show any invasive behaviour locally (see Appendix A for details).

**Figure 2:**
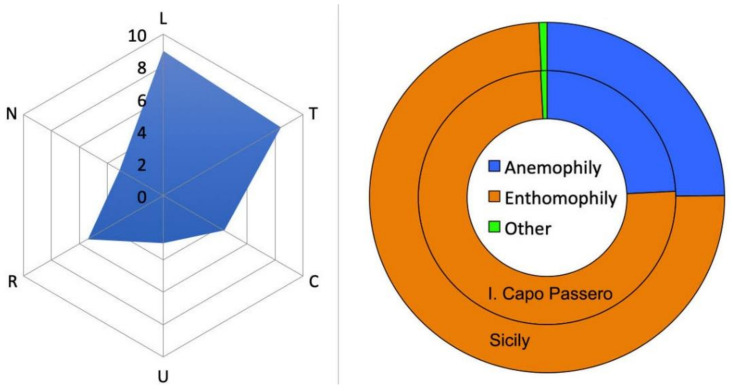
Ecogram of the vascular flora of the Island of Capo Passero (**left**) and target diagram (**right**) comparing the pollination strategies of the phanerogamic flora of Sicily with that of the Island of Capo Passero. Abbreviations: L—light conditions, T—temperature, C—continentality, U soil moisture, R soil reaction, N—soil nutrients.

The ecological fingerprint of the vascular flora of the Island of Capo Passero, based on the Ellenberg’s indicator values [39], suggested the intense solar radiation and the summer drought stress as major environmental drivers on the island, along with a neutral soil reaction and a relative lack of nutrients (Figure 2).

**Figure 3:**
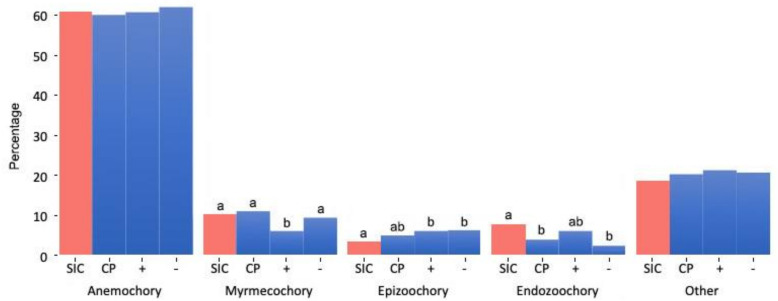
Seed dispersal strategies of the phanerogamic flora of Sicily (SIC) with that of the Island of Capo Passero (CP). The bar “+” refers to the species recorded in the present survey but not observed by Albo (1919, 1957); “−” refers to the species recorded by Albo (l.c.) but not observed by us. Different letters indicate significant differences (*p* < 0.05).

The pollination strategies of the flora of the Island of Capo Passero almost perfectly overlapped those of the whole Sicilian flora, characterized by a large prevalence of enthomophilous taxa (Figure 2). Similarly, no significant variations were observed in the seed dispersal strategies, apart from endozoochory, that is proportionally more represented in the flora of Sicily (Figure 3, first two columns).

The floristic differences with respect to the Albo’s checklists [30,31] suggested that, despite the overall prevalence of anemochorous *taxa*, the vertebrate fauna represents an important vector for the plant colonization of the island and that epizoochorous plants have more chances to survive than endozoochorous. Additionally, the immigration of myrmechocorous *taxa* does not compensate the extinction rate (Figure 3, first three columns). The flora of the Island of Capo Passero hosts several *taxa* of high phytogeographic interest. The most interesting ones are briefly commented in the following paragraphs.

#### 2.1.1. *Aeluropus lagopoides* (L.) Trin. ex Thwaites

This salt-tolerant geophyte usually grows in the gaps of chenopod scrub. Its distribution range includes the Mediterranean Islands, the Sahara and the Indian Subcontinent. Once widespread in Sicily, due to anthropogenic disturbance it currently occurs in few saltmarshes of western and southeastern Sicily. It features among the species assigned to Least Concern (LC) category according to IUCN criteria [40].

#### 2.1.2. *Cichorium spinosum* L.

In Italy, this species is restricted to the SE-Sicilian coasts [31,41], which actually host the north-westernmost isolated population of this chamaephytic plant, quite common from sea level up to more than 1300 m a.s.l. in the East Mediterranean countries, but also widespread in Maltese Islands [42]. In the Island of Capo Passero, *C. spinosum* is localized in small areas along the rocky coast. Orsenigo et al. [40] reports this species as Endagered (EN) according to IUCN criteria.

#### 2.1.3. *Limonium hyblaeum* Brullo

This salt-tolerant hemicryptophyte is considered to be endemic to SE-Sicily, where it is found only between Scoglitti and Capo Passero [43,44,45,46]. Quite surprisingly, it also occurs on the coastal rocky shores of Contrada Faraglione on the island Favignana (western Sicily), where it was already reported by Brullo [45] and was confirmed by S. Pasta and L. Scuderi (October 2004). As for the Island of Capo Passero, one single small population, represented by tiny individuals, was observed growing near the Spanish fortress. Although Orsenigo et al. [47] assigned the IUCN category Least Concern (LC) to this species, during recent times its extent of occurrence is rapidly shrinking, as the species is currently threatened with urban sprawl in most of its growing sites.

#### 2.1.4. *Limonium syracusanum* Brullo

This chamaephyte is endemic to SE-Sicily [44,45]. More in detail, it is only found on the rocky cliffs along the Ionian coasts, between Penisola della Maddalena and Vendicari [41,43,48]. Never observed before on the islet, local population counts only a few individuals, localized in the extreme southern tip of the island. Orsenigo et al. [47] included this species among the Least Concern (LC).

#### 2.1.5. *Poterium spinosum* L.

In Italy, this thorny shrub occurs in Calabria, Apulia and in one single locality of Sardinia [49], while SE Sicily hosts its main population, ranging from the sea level up to 600 m a.s.l.. Recently, Orsenigo et al. [40] confirmed the status Endangered (EN) for the Italian territory.

#### 2.1.6. *Senecio pygmaeus* DC.

This therophyte is reported to be endemic to SE-Sicily, Maltese Archipelago and Lampedusa, growing near the coast, mostly in shallow soil pockets or along the sides of seasonal rock pools. Conti et al. [50] reported it as Endagered (EN) at the regional scale.

#### 2.1.7. *Spergularia heldreichii* E. Simon & P. Monnier

The Island of Capo Passero hosts the only known population of this tiny therophyte in the whole Sicilian territory [38]. This salt-tolerant plant species, with a Mediterranean distribution, grows along the rocky coast, on small pools with sandy soil rich in salt, and subject to temporary flooding. Currently, no risk assessment has been carried out for this species.

### 2.2. Species Turnover, Population Trends and Landscape Evolution

Even if the list compiled by Albo presented a few identification pending issues (see notes in Appendix A for further details), the long time elapsed since his investigation on the Island of Capo Passero and the most recent ones raises some interesting considerations on the extent and direction of local species turnover and induced the authors to try to correlate these trends with local landscape dynamics. Several psammophilous species reported by Albo [30] have not been found anymore, such as *Achillea maritima*, *Eryngium maritimum*, *Echinophora spinosa.* This fact, together with the negative trend of some other coastal plants, such as *Limonium hyblaeum* and *Calamagrostis arenaria* subsp. *arundinacea* (=*Ammophila arenaria*) point out the severe effect of current disturbances (mostly linked to the seagull colony and seasonal tourism), to which have to be added also the sea currents and the wave motion that, in recent years, have determined the erosion of the sandy coast.

As already pointed out by Bergmeier and Dimopoulos [51], when the time lapse between floristic inventories is too large, like in our case, the available lists are often unable to ‘capture’ the ups and downs of local plant metapopulations. The risk of observation gaps is real: on the one hand, during the 30 years after the last census of Albo [31] as much as 48 *taxa* new to the islet have been recorded by Pirola [33] (8 additions), Galletti [35] (16 additions) and by Camatta et al. [36] (24 additions). On the other hand, many of these ‘new entries’ seem to have disappeared once again, as they have not been confirmed neither during the campaign carried out between 1997 and 2000 nor during last surveys (see Appendix A).

According to one of the key assumptions of island biogeography [52], colonization chances are higher—and consequently species turnover is more intense—on the small islets which are very close to large “species sources”: this is the case of the tiny Island of Capo Passero, only 300 metres from the largest and plant species-richest island of the Mediterranean. Hence, it is not surprising if a relevant proportion of the taxa observed by Albo does not occur anymore. For the same reason, several species found for the first time by Cristaudo and Maugeri [37] (e.g., *Hypericum triquetrifolium*, *Lemna minor*, *Vicia bithynica*) have not been confirmed during the last survey, when some other *taxa* (e.g., *Arenaria serpyllifolia*, *Cachrys pungens*, *Plantago afra*, *Spergularia rubra* and *Vicia villosa*) were recorded for the first time (see Appendix A).

### 2.3. Current Vegetation Units

**Figure 4:**
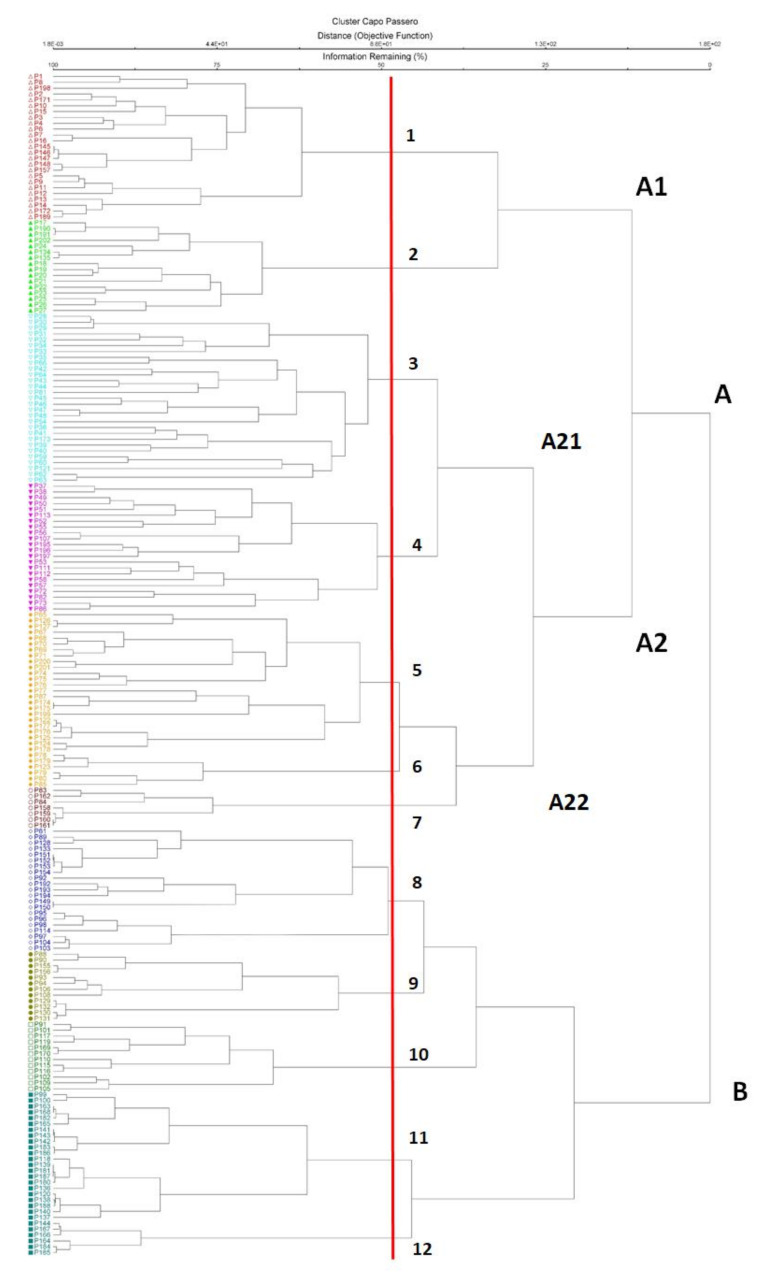
Cluster analysis of the surveyed plant communites: 1. Pistacio lentisci-Chamaeropetum humilis; 2. *Chamaeropo humilis-Sarcopoterietum spinosi*; 3. *Stipellula capensis* and *Asteriscus aquaticus* community; 4. *Parapholido incurvae-Catapodietum balearici*; 5. *Centaureo sphaerocephalae-Ononidetum ramosissimae*; 6. *Medicagini marinae-Ammophiletum australis*; 7. *Salsolo-Cakiletum maritimae*; 8. *Limonietum hyblaei*; 9. *Spergulario bocconei-Mesembryanthemetum nodiflori*; 10. *Crithmo maritimi-Limonietum virgati*; 11. *Limonio virgati-Arthrocnemetum macrostachyi*; 12. *Halimiono portulacoidis-Suaedetum verae*.

The cluster analysis of all vegetation relevés shows two main branches and 12 groups of plots belonging to 10 phytosociological classes (Figure 4, Appendix B and Appendix C).

The first branch is characterized by scrub vegetation (cluster A1) and psammophilous communities together with the annual herbaceous communities (cluster A2); the second one (cluster B) includes the litho-halophilous vegetation linked to rocky coasts. The tallest vegetation found in the island is a maquis dominated by *Chamaerops humilis* and *Pistacia lentiscus*, referred to *Pistacio lentisci-Chamaeropetum humilis* (cluster 1, Table A2), which occurs in many other coastal sites of North-Western and South-Eastern Sicily [12,53,54,55]. This maquis is often replaced by a phrygana-like shrubland dominated by the thorny cushions of *Poterium spinosum* on dry rocky stands with a shallow layer of soil, often representing a degradation serial stage of the coastal maquis. According to Minissale et al. [48], this community may be referred to the association *Chamaeropo-Sarcopoterietum spinosi* (cluster 2, Table A2). The most degraded stage of the vegetation in the inner areas is represented by a community dominated by *Stipellula capensis* and *Asteriscus aquaticus* belonging to *Stipo-Bupleuretalia semicompositi* order (cluster 3, Table A3), which is linked to uncultivated fields. Along the limestone rocky coast, in the small depressions covered by a thin silty-sandy layer, an annual halo-nitrophilous vegetation grows referable to *Parapholido incurvae-Catapodietum balearici,* often mixed with halophilous perennial species, as *Limonium sinuatum* and *Limonium virgatum,* and some annual plants of the class *Stipo-Trachynietea distachyae* (cluster 4, Table A2). Psammophilous vegetation only occurs in the few dune remnants near the southwestern shore of the islet. Across the landward gradient, the first community is the *Salsolo-Cakiletum maritimae* (cluster 7, Table A4), which forms the first vegetation belt along the shoreline, colonizing the sandy surfaces subject to accumulation of organic matter stranded during storm surges. This species-poor community is formed by late-flowering, scattered therophytes, namely *Cakile maritima* and *Salsola tragus*. The coastal erosion and strong winds induced a loss of the dunal vegetation dominated by *Calamagrostis arenaria* subsp. *arundinacea* (*Ammophiletea*), which normally occurs on well-developed shifting dunes. The floristic remnants include other psammophytes, such as *Pancratium maritimum*, *Scolymus hispanicus* and *Euphorbia paralias* (cluster 6, Table A4). Probably also because of the disappearance of *Thynopyrum junceum*, the latter species tends to become dominant on the embryonic dunes closer to the sea. Inwards, the retrodunal sand hosts a community dominated by *Centaurea sphaerocephala*, *Ononis natrix* subsp. *ramosissima* and *Euphorbia terracina*. This plant community can be referred to the *Centaureo sphaerocephalae-Ononidetum ramosissimae* (cluster 5, Table A4), growing on relatively stable and compact sandy soils [56]. The gaps among the above-mentioned perennial herbs and shrubs are covered by ephemeral psammophilous swards dominated by *Silene nicaensis* and *Senecio coronopifolius* belonging to *Vulpietalia* order. However, most of the island’s coasts are charaterized by rock outcrops, colonized by communities belonging to the class *Crithmo-Staticetea*. Among these, the most widespread is the *Crithmo maritimi-Limonietum virgati* (cluster 10, Table A5), which is dominated by *Limonium virgatum* and *Crithmum maritimum*, and few other species, such as *L. sinuatum* and *Cichorium spinosum*. Conversely, the *Limonietum hyblaei*, already reported for the adjacent coast by Bartolo et al. [41], covers a very restricted area of the island (cluster 8, Table A5). These communities are often replaced by nuclei with *Limonium virgatum* and *L. sinuatum*, as a consequence of the anthropogenic disturbance. The brackish rock pools amidst the *Crithmo-Staticetea* vegetation and the seasonally inundated sediments behind the cliffs are home to a vegetation characterized by *Arthrocaulon meridionalis*, a succulent chenopod scrub usually linked to seasonally inundated salt marshes, and *Limonium virgatum*. This vegetation (cluster 11, Table A6) is quite similar to the *Limonio virgati-Arthrocnemetum macrostachyi*, an association belonging to the class *Salicornietea fruticosae*, described by Biondi et al. [57] from southern Apulia and already reported for Sicily by Minissale and Sciandrello [20]. Furthermore, on the island, in the hyper-nitrified areas, rarely subject to submersion, a vegetation dominated by *Suaeda vera* grows, with few other halophilous species, such as *Halimione portulacoides*, *Arthrocaulon meridionalis*, *Aeluropus lagopoides*, *Limonium virgatum* (cluster 12, Table A6). This halo-subnitrophilous vegetation is referable to *Halimiono portulacoidis-Suaedetum verae*, an association belonging to *Suaedion verae*, alliance of *Salicornietea fruticosae* [58]. The thin layer of sediments accumulating on limestones with horizontal attitude, intensively subject to seabird droppings and to human seasonal trampling, are colonized by a halo-nitrophilous annual community dominated by *Mesembryanthemum nodiflorum* and *Beta maritima,* belonging to *Spergulario bocconei-Mesembryanthemetum nodiflori* (cluster 9, Table A5), which usually forms a dense carpet covering large surfaces. In addition, to the plant communities described above, some other monophytic or floristically poor vegetation units have been observed in the island. In particular, the small temporary basins amidst the clearings of shrub vegetation are colonized by an ephemeral vegetation with *Polypogon subspathaceus*, while the coastal rocky crevices are characterized by an annual sub-halophilous community dominated by *Senecio pygmaeus*. Inland disturbed stands, with slightly humid soils, are colonized by *Arundo donax*, probably introduced on the island because its stems were, until a recent past, a precious material for construction, basketry and stakeing. Inland sandy soils, which were cultivated in the past, are colonized by *Saccharum biflorum*, probably also introduced by man as a windbreak. Finally, the banks of a small artificial basin for rainwater collection are colonized by *Typha domingensis*.

### 2.4. Changes Affecting Local Vegetation Patterns over Last Ha Century

**Figure 5:**
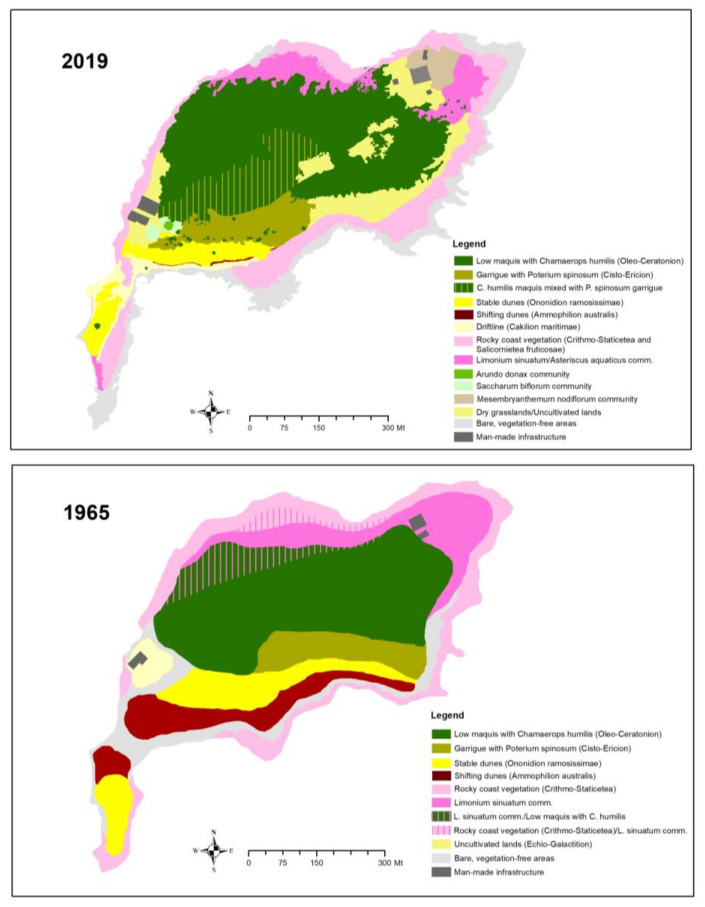
Comparison between the vegetation map published by Pirola (1965) and the one produced by the authors combining the interpretation of recent aerial photos (2019) and field surveys. The list of the plant communities is shown in the legend included in each map. The corresponding habitats (according to European Directive 92/43 CEE) for each plant communities are listed in Table 1.

Interesting clues on the recent evolution of the landscape are given by the comparison between the vegetation map of Pirola [34] with a new one based on aerial photographs dating back to 2019 (Table 1, Figure 5).

**Figure A1:**
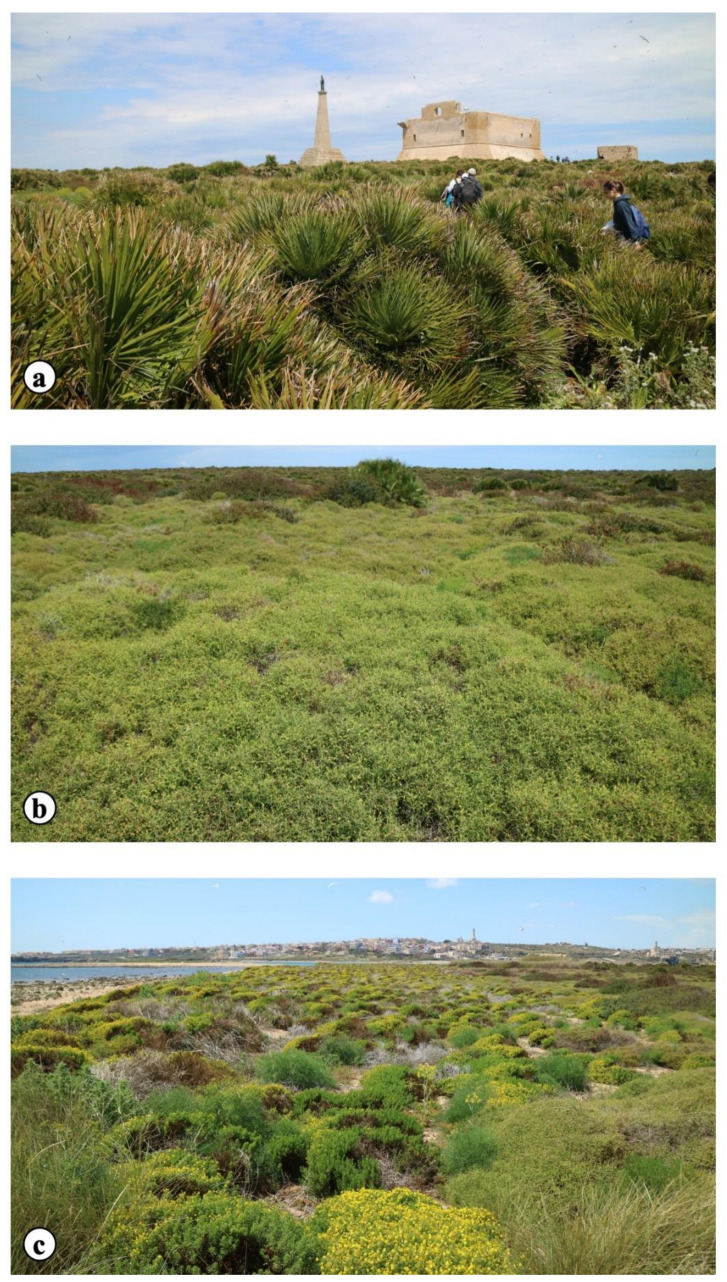
Photo plate illustrating different habitat types on the Island of Capo Passero: (**a**) *Chamaerops humilis* maquis (*Pistacio lentisci-Chamaeropetum humilis*; (**b**) *Poterium spinosum* garrigue (*Chamaeropo humilis-Sarcopoterietum spinosi*); (**c**) Stable dunes with *Ononis ramosissima* (*Centaureo sphaerocephalae-Ononidetum ramosissimae*).

**Figure A2:**
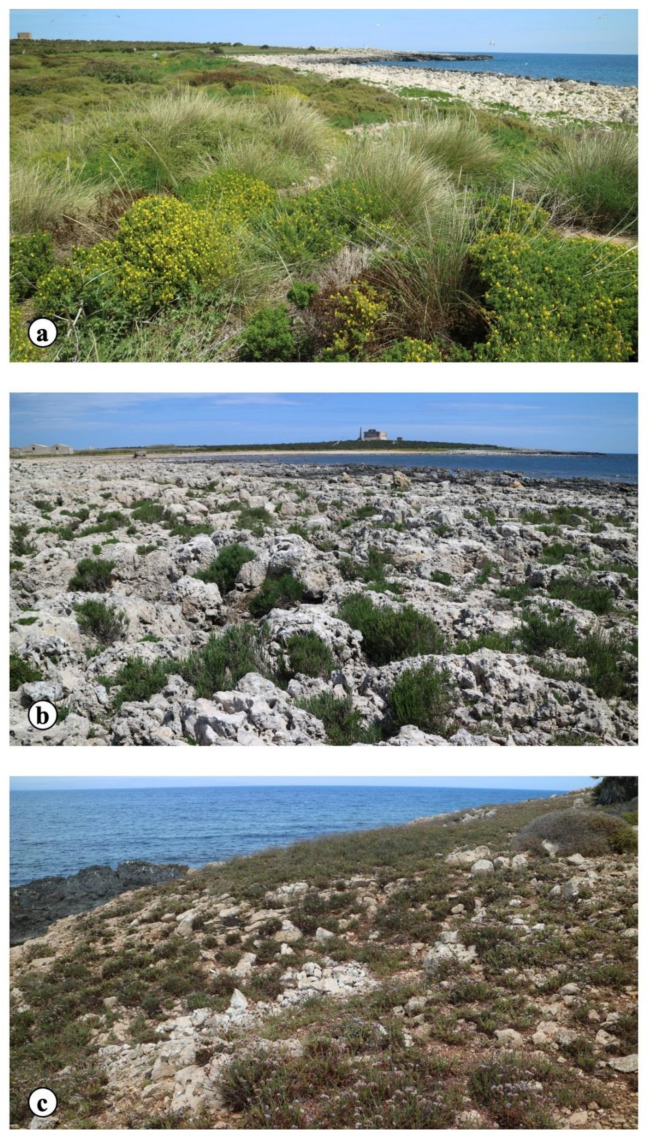
Photo plate illustrating different habitat types on the Island of Capo Passero: (**a**) last strips of vegetation with *Calamagrostis arundinacea* (*Medicagini marinae-Ammophiletum australis*) mixed with *Ononis ramosissima*; (**b**) rocky coast vegetation (*Limonio virgati-Arthrocnemetum macrostachyi*); (**c**) Rocky coast vegetation (*Limonietum hyblaei, Crithmo maritimi-Limonietum virgati*).

Thanks to repeated cross-checks supported by field surveys, the photointerpretation of the aerial photos enabled identifying 12 vegetation types, 10 of which represent habitats of community interest according to the EU ‘Habitats’ Directive 92/43 (Figure A1 and Figure A2).

The comparison of the two maps shows no significant differences in the area occupied by the vegetation. However, some variation in the area of occupancy of some vegetation units has been detected (Table 1). More in detail, the data show a strong shrinking of the vegetation of shifting dunes (*Medicagini marinae-Ammophiletum arenarii*) which decreased from 3 ha (8.7%) in 1965 to 0.05 ha (0.1%) in 2019, as well as of the *Ononis ramosissima* community (*Centaureo-Ononidetum ramosissimae*), which decreased from 3.6 ha (10%) to 1.8 (5.0%) ha. The cover of the most common plant community of the island, i.e., the dwarf maquis with *Chamaerops humilis*, has undergone a slight reduction, from 12.9 ha (36.0%) to 11.0 ha (31.0%). Inversely, the *Poterium spinosum* garrigue, together with the *Chamaerops humilis* maquis mixed with *Poterium spinosum* garrigue, increased from 2.4 ha (6.7%) to 5 ha (14.3%), as well as the dry grasslands (*Stipo-Trachynietea distachyae*) from 0.6 ha (1.7%) to 3.5 ha (10%).

The observed strong reduction in the dune system has been recorded in many other places along the Mediterranean coast [59,60,61,62,63]. These results highlight that human pressure directly and indirectly triggered the disruption of coastal dune systems, hugely affecting both the structure and the function of the local psammophilous plant communities. Therefore, the study case of Capo Passero Island, despite the modest size of the surveyed area, is a very representative example of how, within a few decades, seasonal trampling by tourists can destroy a dune system with direct negative consequences on the species and the communites/habitats linked to sandy shores. For the same reasons, some psammophilous species, recorded by Albo [30], such as *Achillea maritima, Eryngium maritimum* and *Echinophora spinosa*, have totally disappeared. This is in contrast to the conditions observed by Pirola [33], who depicted a vegetation transect (North–South section) indicating a well-preserved dune system in the southern part of the island.

## 3. Materials and Methods

### 3.1. Study Area

**Figure 6:**
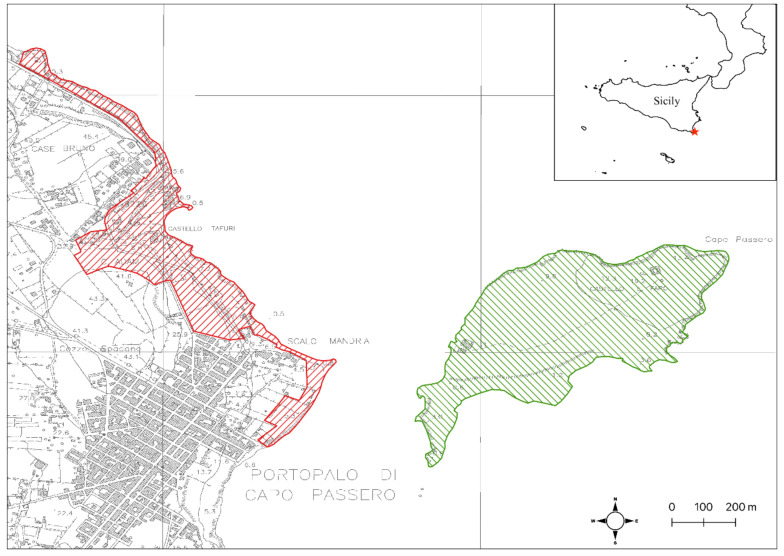
Proposed enlargement on the mainland of the boundaries of the SAC ITA090001 “Isola di Capo Passero”.

The Island of Capo Passero (latitude: 36°41′13″ N; longitude: 15°08′56″ E) has a surface of less than 36 ha and is located in front of the little town of Portopalo di Capo Passero, which corresponds to the south-easternmost corner of Sicily. The highest point of the island is 21 m. a.s.l., and currently hosts a lighthouse (Figure 6).

Despite its small size, the islet is characterized by many different types of sediments and rock outcrops [64,65]. From the most recent to the oldest one, these are: (1) recent sands and coastal dunes (Holocene), (2) limestones with Nummulites (Eocene) in the eastern part of the islet, (3) calcirudites with Rudistae (Upper Cretaceous) on northern sea cliffs and (4) base-rich vulcanites (Upper Cretaceous) along the eastern side.

The topography of the sea channel that separates the islet from Capo Passero, today c. 2.5 m in depth and 300 m in width, has been subject to continuous changes due to intense marine currents [66]. A long list of reports made by military engineers, maps, drawings, geographic treatises, sailor books testifie complex and long-lasting alternation of phases of closing and opening of this channel, partly driven by sea currents. After being isolated for about two centuries, around the mid 18th century the Island of Capo Passero was connected to the mainland by a thick sandy strip. Since then, the islet has been uninterruptedly separated from Sicily, as confirmed by numerous sources [29].

In order to prevent the incursions of pirates and to protect the south-eastern Sicilian coasts against them, the Spanish Government decided to build a fortress on the islet, whose construction was finished in 1635 [67]. Until mid 20th century, the islet was frequently visited by fishermen and people collecting the leaves of dwarf palms, whose fibers were used to produce several items (baskets, fans, hats, ropes, etc.). Nowadays, the Island of Capo Passero is uninhabited but it is home to a very large colony of yellow-legged seagulls (*Larus michahellis* Naumann), which induced a sharp increase in soil nitrogen content, significantly modifying local flora and vegetation as elsewhere in the Mediterranean [13].

Local climate is typically Mediterranean; based on the data from the nearby thermo-pluviometric station of Cozzo Spadaro (just 2.5 km from the study area), the mean annual temperature is 18.5 °C, while the mean annual precipitation, concentrated over the autumn and winter seasons, is 381 mm. According to the bioclimatic classification proposed by Rivas-Martiínez et al. [68,69,70], the investigated territory should be referred to the Mediterranean pluviseasonal oceanic bioclimate, with low thermomediterranean thermotype and dry ombrotype [71].

### 3.2. Data Sets and Data Processing

The collected plant specimens, were pressed in a plant press, dried on a plant dryer and stored in the Herbarium of the University of Catania (CAT). Specimens were identified following the second edition of the Flora d’Italia [72,73,74,75]. The floras by Fiori and Paoletti [76] and Fiori [77] were consulted as well, to ensure the best possibile interpretation of the plant names adopted in the lists published by Albo [30,31].

The family and the scientific name according to the Portal to the Flora of Italy [78] were assigned to each *taxon*, along with the following traits, extracted from the second edition of Flora d’Italia [72,73,74,75]: life form, chorotype, Ellenberg indicator values, pollination and seed dispersal strategies. The Pearson’s χ2 test was used to compare some of the traits with those of the Sicilian flora and to get some clues on the variations occurred between the Albo floristic surveys [30,31] and the current vascular flora of the Island of Capo Passero. Statistical analyses were performed using R 4.0.3 [79] and the ggplot2 package [80] for data visualization.

The vegetation was sampled according to the phytosociological method [81]. The total amount of the vegetation data consisted of 202 phytosociological relevés, 191 of them issuing from recent field surveys carried out by the authors and 11 taken from literature [33]. For the numerical vegetation classification, the original Braun-Blanquet’s sampling scale was transformed into ordinal scale according to van der Maarel [82] and a hierarchical clustering was performed by means of the PC-ORD 6 software. Clusters were interpreted basing on the syntaxonomic scheme by Mucina et al. [83] and other phytosociological papers from Sicily [10]. The detected vegetation units were then correlated to habitats of community interest following the Italian Interpretation Manual for the Habitats of Directive 92/43/EEC [84].

The current area of occupancy and distribution of the vegetation units was mapped using ArcGis 10.6 (ESRI Inc., Redlands, CA, USA). In order to perform a diachronic comparison between the past [34] and the current vegetation patterns, the interpretation of aerial images taken in 2019 (source: Google Earth) was validated by means of repeated field surveys.

## 4. Conclusions

This study has shown how the islet of Capo Passero has great floristic peculiarities and at the same time considerable vulnerability that can cause extinctions/decline of populations and reduction/alterations of habitats under stress. For this reason, it is important to plan and implement targeted conservation actions similar to what has been proposed for other small Mediterranean islands [4,85]. Already 50 years ago, considering the botanical interest of the Island of Capo Passero, Pirola [86] recommended its strict protection. The “Isola di Capo Passero” nature reserve was established on 16 May 1995 by decree of the Sicilian Regional Government. However, in 1998, after a long legal dispute following an appeal against the reserve made by the private owner of the island, the TAR (Regional Administrative Tribunal) canceled the protected area. Fortunately, the conservation measures of the Habitats Directive (EU 92/43) have allowed so far guaranteeing lasting protection of the island’s naturalistic values. In fact, after the SCI proposal dating back to 1995 (ITA090001 “Isola di Capo Passero”), this Natura 2000 site was promoted as a Special Conservation Area (SAC) in 2017. Unfortunately, the lack of an encharged management body for this Natura 2000 site does not allow the implementation of active conservation and management policies (e.g., the regulation of visitors’ access by creating of dedicated paths for seasonal tourists, who represent a serious threat for all the coastal habitats —especially 1210, 2120 and 2210—due to trampling and other kinds of disturbance, the control/eradication of the few occurring alien vascular plant species. This is a problem in common with all the Sicilian Natura 2000 sites which do not fall within the protected areas whose management is regulated by regional laws (e.g., nature reserves or regional parks) and is perfomed by public bodies and private NGOs.

The peculiar interest of this site for botanists is well documented since centuries (Pasta, *submitted*) and its paramount importance until present times its confirmed by recent inclusion among the Italian Important Plant Areas (IPA SIC17) according to Blasi et al. [87]. Moreover, the island and the nearby coast have an exceptional geological and paleontological interest, highlighted since the mid 19th century, for its fossil-rich (rudists, corals) outcropping limestones dating back to Cretaceous [64]. The historical and cultural sites by the island’s castle and the Roman remains on the adjacent coast are no less important [88]. Unfortunately, all these natural and historical-cultural values are not protected adequately by local institutions, which instead have recently promoted some actions aiming at consolidating the rocky coast with concrete, damaging the coastal communities of the mainland promontory and deliberately introducing highly invasive alien plants such as *Carpobrotus* spp. This plant should be eradicated or monitored to avoid its introduction on the islet. Therefore, the authors suggest extending the limits of the SAC, so to include the *Cichorium spinosum* populations located in the promontory of Capo Passero (Figure 6). This regionally rare and highly localized species characterizes two different habitats of community interest, i.e., the “Vegetated sea cliffs of the Mediterranean coasts with endemic spp.” (Habitat code: 1240) and the “*Sarcopoterium spinosum* phryganas” (Habitat code: 5420). The enlargement of the Natura 2000 site, the designation of a qualified management body and the acquisition of the island as public ownership could guarantee a better management of this precious territory and the preservation not only of its naturalistic values but also the cultural heritage as a whole which originated, as in other Mediterranean sites, from with the harmonious balance between those values [89]. Although much still needs to be done, our map of plant communities (Figure 5) is a solid basis for the management and monitoring of the habitats to be protected over time.

This paper is addressed to all who believe that the site of Capo Passero deserves to be adequately managed and preserved, so that future generations will continue to appreciate its natural highlights, studied, described and appreciated since centuries by scientists, poets, historians, painters, travellers and geographers from all over Europe.

## Figures and Tables

**Table 1:** Comparison of the surface covered by the different vegetation units/habitats based on the vegetation map of Pirola [34] and authors’ recent vegetation map (2019).

		1950		2019	
Vegetation	Habitat	ha	%	ha	%
Stable dunes (*Ononidion ramosissimae*)	2210	3.6	10.0	1.8	5.0
Shifting dunes (*Ammophilion australis*)	2120	3.1	8.7	0.05	0.1
Driftline (*Cakilion maritimae*)	1210	-	-	0.88	2.5
Low maquis with *Chamaerops humilis* (*Oleo-Ceratonion*)	5330	12.9	36.0	10.89	30.7
*Chamaerops humilis* maquis mixed with *Poterium spinosum* garrigue	-	-	-	3.05	8.6
Garrigue with *Poterium spinosum* (*Cisto-Ericion*)	5420	2.4	6.7	2.04	5.8
*Limonium sinuatum* community (incl. *Parapholido-Catapodietum balearici*)	1240	4.5	12.6	2.33	6.6
Rocky coast vegetation *(Crithmo-Limonietea* and *Salicornietea fruticosae)*	1240, 1310, 1420	5.6	15.6	5.82	16.4
*Mesembryanthemum nodiflorum/Beta maritima* community	1310	-	-	0.64	1.8
Dry grasslands (*Stipo-Trachynietea distachyae*)	6220	0.6	1.7	3.53	10.0
*Saccharum biflorum* community	-	-	-	0.19	0.5
*Arundo donax* community	-	-	-	0.03	0.1
Bare, vegetation-free areas	-	3.1	8.7	3.85	10.9
Man-made infrastructure	-	-	-	0.39	1.0
		**35.8**		**35.49**	

## Data Availability

All data presented in the manuscript are available in the form of tables and figures in the manuscript.

## Appendix A

**Table A1:** Species list of the vascular plants recorded from the Island of Capo Passero (SE Sicily). Alien species are marked in red. Abbreviations of Ellenberg indicator values: L—light conditions, T—temperature, C—continentality, U soil moisture, R soil reaction, N—soil nutrients. Abbreviations of pollination strategies: AP—anemophily, BP—ballistic, CG—cleistogamy, EP—enthomophily, HP—hydrophily. Abbreviations of seed dispersal strategies: AC—anemochory, AUC1—ballistic autochory, AUC2—hygronastic autochory, BC—barochory, HC—hydrochory, ZC1—epizoochory, ZC2—endozoochory, and ZC3—myrmecochory.

N°	Species	Family	Life Form	Chorotype	This Paper	Albo (1919; 1957) *	Pirola (1959)	Galletti (1988)	Camatta et al. (1990) **	Cristaudo and Maugeri (2005)	L	T	C	U	R	N	S	Pollination Strategies - Seed Dispersal Strategies
1	*Achillea maritima* (L.) Ehrend. & Y.P.Guo subsp. *maritima*	Asteraceae	Ch suffr	Medit.-Atl.		1	1				11	10	4	1	3	1	2	EP − AC
2	*Aeluropus lagopoides* (L.) Trin. ex Thwaites	Poaceae	G rhiz	Medit.-Turan.	1			1		1	11	12	3	4	8	1	3	AP − AC
3	*Agave americana* L. subsp. *americana*	Asparagaceae	P caesp	N-Americ.	1			1		1	9	10	2	2	X	2	0	EP − AC + AUC1
4	*Ajuga iva* (L.) Schreb. subsp. *iva*	Lamiaceae	Ch suffr	Steno-Medit.	1	1				1	8	8	4	3	7	2	0	EP − AC + ZC3
5	*Ajuga iva* (L.) Schreb. subsp. *pseudoiva* (DC.) Briq.	Lamiaceae	Ch suffr	Steno-Medit.	1					1	8	8	4	3	7	2	0	EP − AC + ZC3
7	*Allium ampeloprasum* L.	Amaryllidaceae	G bulb	SE-Europ.	1					1	7	7	5	3	6	5	0	EP − AUC1
6	*Allium nigrum* L.	Amaryllidaceae	G bulb	Steno-Medit.		1					10	9	4	3	6	4	0	EP − AUC1
8	*Allium roseum* L. subsp. *roseum*	Amaryllidaceae	G bulb	Steno-Medit.	1					1	8	8	4	3	6	5	0	EP − AUC1
9	*Allium subhirsutum* L. subsp. *subhirsutum*	Amaryllidaceae	G bulb	W-Medit.		1					8	9	4	2	4	2	0	EP − AUC1
10	*Andropogon distachyos* L.	Poaceae	H caesp	Paleotrop.	1					1	11	7	4	2	8	2	0	AP − AC
11	*Andryala integrifolia* L.	Asteraceae	T scap	Medit.-Atl.	1					1	8	9	3	2	2	1	0	EP − AC
12	*Anisantha fasciculata* (C.Presl) Nevski subsp. *fasciculata*	Poaceae	T scap	S-Medit.	1					1	11	11	5	2	6	2	0	AP − AUC2
13	*Anisantha madritensis* (L.) Nevski subsp. *madritensis*	Poaceae	T scap	Wide-Medit.	1					1	8	7	5	3	X	1	0	AP − AUC2
14	*Anisantha rigida* (Roth) Hyl.	Poaceae	T scap	Paleosubtrop.	1					1	8	8	5	4	6	5	0	AP − AUC2
15	*Anthemis secundiramea* Biv.	Asteraceae	T scap	S-Medit.	1					1	11	11	5	1	3	1	0	EP − AC
16	*Anthoxanthum ovatum* Lag.	Poaceae	T scap	W-Medit.					1		7	8	4	3	3	2	0	AP − AUC2
17	*Arenaria leptoclados* (Rchb.) Guss. subsp. *leptoclados*	Caryophyllaceae	T scap	Paleotemp.	1	1	1			1	9	9	5	2	3	1	0	EP − AC + ZC3
18	*Arenaria serpyllifolia* L. subsp. *serpyllifolia*	Caryophyllaceae	T scap	Subcosmop.	1						9	5	X	4	X	X	0	EP − AC + ZC3
19	*Arisarum vulgare* O.Targ.Tozz. subsp. *vulgare*	Araceae	G rhiz	Steno-Medit.	1	1		1		1	6	8	4	4	4	4	0	EP − ZC2
20	*Arthrocaulon meridionalis* Es.Ramírez, Rufo, Sánchez Mata, V.Fuente	Chenopodiaceae	Ch succ	Steno-Medit.	1	1	1	1		1	11	9	4	8	9	7	3	AP − AC
21	*Arundo donax* L.	Poaceae	G rhiz	Subcosmop.						1	8	9	5	5	5	6	0	AP − AC
22	*Asparagus acutifolius* L.	Asparagaceae	G rhiz	Steno-Medit.	1	1	1	1		1	6	9	4	2	5	5	0	AP + EP − ZC2
23	*Asphodelus fistulosus* L.	Asparagaceae	H scap	Paleosubtrop.	1					1	11	8	5	2	4	2	0	EP − AUC1
24	*Asphodelus ramosus* L. subsp. *ramosus*	Asphodelaceae	G rhiz	Steno-Medit.	1	1		1	1	1	10	9	4	2	3	5	0	EP − AUC1
25	*Asteriscus aquaticus* (L.) Less.	Asteraceae	T scap	Steno-Medit.	1	1	1	1		1	10	9	4	7	7	7	0	EP − AC + HC
26	*Astragalus boeticus* L.	Fabaceae	T scap	S-Medit.	1	1				1	11	11	5	1	3	1	0	EP − BC + AUC1
27	*Atractylis cancellata* L.	Asteraceae	T scap	S-Medit.	1					1	11	8	5	2	6	2	0	EP − AC
28	*Avellinia festucoides* (Link) Valdés & H.Scholz	Poaceae	T scap	Steno-Medit.	1					1	10	9	4	2	5	1	0	AP − AC
29	*Avena barbata* Pott ex Link	Poaceae	T scap	Medit.-Turan.	1	1	1			1	8	8	5	3	7	2	0	AP − AUC2
30	*Avena sterilis* L. subsp. *sterilis*	Poaceae	T scap	Wide-Medit.	1					1	8	9	5	3	6	4	0	AP − AUC2
31	*Barlia robertiana* (Loisel.) Greuter	Orchidaceae	G bulb	Steno-Medit.	1					1	7	9	4	3	6	2	0	EP − AC
32	*Bellardia trixago* (L.) All.	Orchidaceae	T scap	Wide-Medit.	1	1				1	8	8	5	3	3	3	0	EP − AUC1
33	*Bellis annua* L. subsp. *annua*	Asteraceae	T scap	W-Medit.	1					1	6	9	4	7	2	2	0	EP − AC
34	*Bellis sylvestris* Cirillo	Asteraceae	H ros	Steno-Medit.	1					1	5	8	4	3	3	3	0	EP − AC
35	*Beta vulgaris* L. subsp. *maritima* (L.) Arcang.	Chenopodiaceae	H scap	Wide-Medit.	1	1		1		1	10	7	5	6	6	5	1	AP − AC
36	*Blackstonia perfoliata* (L.) Huds.	Gentianaceae	T scap	Wide-Medit.	1					1	8	7	5	X	9	4	0	EP − AUC1
37	*Borago officinalis* L.	Boraginaceae	T scap	Wide-Medit.	1	1				1	7	8	5	3	5	5	0	EP − ZC3
38	*Brachypodium distachyon* (L.) P.Beauv.	Poaceae	T scap	Medit.-Turan.	1				1	1	10	9	3	1	3	2	0	AP − AC
39	*Brachypodium retusum* (Pers.) P.Beauv.	Poaceae	H caesp	W-Medit.	1					1	11	10	3	2	5	2	0	AP − AUC2
40	*Briza maxima* L.	Poaceae	T scap	Paleosubtrop.	1	1			1	1	8	10	5	2	4	1	0	AP − AC
41	*Bromus hordeaceus* L. subsp. *hordeaceus*	Poaceae	T scap	Subcosmop.	1	1	1				7	6	5	X	X	X	0	AP − AUC2
42	*Bromus scoparius* L.	Poaceae	T scap	Steno-Medit.	1						11	9	4	2	6	2	0	AP − AUC2
43	*Cachrys libanotis* L.	Apiaceae	H scap	W-Medit.	1	1				1	11	9	5	3	7	2	0	EP − BC
44	*Cachrys pungens* Jan ex Guss.	Apiaceae	H scap	SW-Medit.	1						11	11	5	3	7	2	0	EP − BC
45	*Cachrys sicula* L.	Apiaceae	H scap	W-Medit.	1			1			11	10	4	2	5	2	0	EP − BC
46	*Cakile maritima* Scop. subsp. *maritima*	Apiaceae	T scap	Medit.-Atl.	1	1		1		1	9	8	2	6	X	8	3	EP − AC + HC
47	*Calamagrostis arenaria* (L.) Roth subsp. *arundinacea* (Husn.) Banfi, Galasso & Bartolucci	Poaceae	G rhiz	Wide-Medit.	1	1	1	1	1	1	6	5	5	5	5	5	0	AP − AC
48	*Calendula arvensis* (Vaill.) L.	Asteraceae	T scap	Wide-Medit.	1	1				1	7	8	5	3	8	5	0	EP − AC + BC + ZC
49	*Campanula erinus* L.	Campanulaceae	T scap	Steno-Medit.	1	1				1	7	8	4	2	X	1	0	EP − AC
50	*Capparis orientalis* Veill.	Capparaceae	NP	Steno-Medit.	1	1		1		1	9	10	5	2	5	1	2	AP + EP − ZC2 + ZC3
51	*Carduus argyroa* Biv.	Asteraceae	T scap	Steno-Medit.	1					1	7	8	4	3	7	7	0	EP − AC
52	*Carex divisa* Huds.	Cyperaceae	G rhiz	Medit.-Atl.	1		1			1	8	8	2	3	5	3	0	AP − AC
53	*Carex flacca* Schreb. subsp. *erythrostachys* (Hoppe) Holub	Cyperaceae	G rhiz	Europ.	1					1	7	5	5	6	8	X	0	AP − ZC
54	*Carlina corymbosa* L.	Asteraceae	H scap	Steno-Medit.	1						6	X	4	2	X	4	0	EP − AC
55	*Carlina gummifera* (L.) Less.	Asteraceae	H ros	S-Medit.	1				1	1	11	11	5	1	7	1	0	EP − AC
56	*Carlina hispanica* Lam. subsp. *globosa* (Arcang.) Meusel & Kästner	Asteraceae	H scap	Steno-Medit.	1				1	1	X	X	X	X	X	X	X	EP − AC
57	*Carlina lanata* L.	Asteraceae	T scap	Steno-Medit.	1					1	7	7	4	3	6	2	0	EP − AC
58	*Carlina sicula* Ten. subsp. *sicula*	Asteraceae	H scap	Endem. Ital.						1	11	11	3	1	2	1	0	EP − AC
59	*Carthamus lanatus* L.	Asteraceae	T scap	Wide-Medit.	1				1	1	10	8	5	3	5	6	0	EP − AC
60	*Catapodium balearicum* (Willk.) H.Scholz	Poaceae	T scap	Medit.-Atl.	1	1	1	1	1	1	11	10	3	1	X	1	2	AP − AC
61	*Catapodium pauciflorum* (Merino) Brullo, Giusso, Miniss. & Spamp.	Poaceae	T scap	Medit.-Atl.	1						X	X	X	X	X	X	X	AP − AC
62	*Catapodium rigidum* (L.) C.E.Hubb. subsp. *rigidum*	Poaceae	T scap	Wide-Medit.	1	1	1			1	8	8	5	2	5	4	0	AP − AC
63	*Centaurea melitensis* L.	Asteraceae	T scap	Pantrop.	1	1				1	10	12	5	2	X	5	0	EP − AC + ZC3
64	*Centaurea sphaerocephala* L. subsp. *sphaerocephala*	Asteraceae	H scap	W-Medit.	1		1	1		1	11	10	4	1	X	1	0	EP − AC + ZC3
65	*Centaurium erythraea* Rafn subsp. *erythraea*	Gentianaceae	H bienn	Eurasiat.		1					8	6	5	5	6	X	0	EP − AC
66	*Centaurium pulchellum* (Sw.) Druce subsp. *pulchellum*	Gentianaceae	T scap	Paleotemp.	1		1			1	9	6	7	7	9	3	0	EP − AC
67	*Centaurium tenuiflorum* (Hoffmanns. and Link) Fritsch subsp. *tenuiflorum*	Gentianaceae	T scap	Paleotemp.	1	1				1	9	8	5	7	7	2	0	EP − AC
68	*Cerastium glomeratum* Thuill.	Gentianaceae	T scap	Subcosmop.		1					7	X	5	5	5	5	0	EP − AC + ZC3
70	*Cerastium semidecandrum* L.	Caryophyllaceae	T scap	Cosmop.	1					1	8	7	5	4	X	X	0	EP − AC + ZC3
71	*Cerinthe major* L. subsp. *major*	Boraginaceae	T scap	Steno-Medit.	1	1				1	7	8	4	4	5	9	0	EP − BC
72	*Chamaerops humilis* L.	Arecaceae	NP	W-Medit.	1	1	1	1	1	1	11	10	3	1	4	1	0	AP + EP − BC + ZC
73	*Charybdis pancration* (Steinh.) Speta	Asparagaceae	G bulb	Steno-Medit.	1			1	1	1	11	10	3	1	4	2	X	EP − AUC1
74	*Chenopodiastrum murale* (L.) S.Fuentes, Uotila & Borsch	Chenopodiaceae	T scap	Subcosmop.	1					1	8	7	5	4	X	9	0	AP − AC
75	*Chenopodium album* L. subsp. *album*	Chenopodiaceae	T scap	Subcosmop.	1					1	7	7	5	4	5	7	0	AP − AC
76	*Cichorium spinosum* L.	Asteraceae	Ch suffr	Steno-Medit.	1	1	1	1	1	1	12	10	3	1	5	1	0	EP − AC + HC1
77	*Clinopodium nepeta* (L.) Kuntze subsp. *nepeta*	Lamiaceae	H scap	Steno-Medit.	1					1	5	7	5	3	9	3	0	EP − AC
78	*Clinopodium nepeta* (L.) Kuntze subsp. *spruneri* (Boiss.) Bartolucci & F.Conti	Lamiaceae	H scap	Orof. SE-Europ.		1					5	7	5	3	9	3	0	EP − AC
79	*Convolvulus althaeoides* L.	Convolvulaceae	H scand	W-Medit.	1					1	8	9	4	3	5	2	0	EP − BC
80	*Convolvulus elegantissimus* Mill.	Convolvulaceae	H scand	E-Medit.		1					8	10	5	3	5	2	0	EP − BC
81	*Crassula tillaea* Lest.-Garl.	Crassulaceae	T scap	Medit.-Atl.	1					1	X	X	X	X	X	X	X	EP − AC
82	*Crithmum maritimum* L.	Apiaceae	Ch suffr	Wide-Medit.	1	1	1	1		1	11	8	2	1	X	1	3	EP − AC
83	*Crocus longiflorus* Raf.	Iridaceae	G bulb	Subendem.				1			9	8	4	2	6	2	0	EP − AUC1 + ZC3
84	*Cuscuta planiflora* Ten.	Convolvulaceae	T par	Wide-Medit.	1					1	8	7	5	X	X	X	0	EP − AC + ZC
85	*Cutandia maritima* (L.) Benth. ex Barbey	Poaceae	T scap	Steno-Medit.	1	1	1			1	11	10	3	1	X	1	2	AP − AC
86	*Cynara cardunculus* L. subsp. *cardunculus*	Asteraceae	H scap	Steno-Medit.	1	1		1		1	10	9	4	5	6	3	0	EP − AC
87	*Cynodon dactylon* (L.) Pers.	Poaceae	G rhiz	Cosmop.	1				1	1	8	8	5	4	X	4	0	AP − AC
88	*Cynoglossum cheirifolium* L.	Boraginaceae	H bienn	Steno-Medit.		1					11	9	4	3	7	7	0	EP − ZC1
89	*Cynoglossum clandestinum* Desf.	Boraginaceae	H scap	W-Medit.		1					11	9	5	3	7	7	0	EP − ZC1
90	*Cynoglossum creticum* Mill.	Boraginaceae	H bienn	Wide-Medit.	1	1				1	11	9	5	3	X	7	0	EP − ZC1
91	*Cytisus infestus* (C.Presl) Guss. subsp. *infestus*	Fabaceae	P caesp	Steno-Medit.	1					1	X	X	X	X	X	X	X	EP − BC + AUC1
92	*Dactylis glomerata* L. subsp. *glomerata*	Poaceae	H caesp	Paleotemp.				1			7	6	5	4	5	6	0	AP − AC
93	*Dactylis glomerata* L. subsp. *hispanica* (Roth) Nyman	Poaceae	H caesp	Steno-Medit.	1				1	1	7	6	5	4	5	6	1	AP − AC
94	*Daucus carota* L. subsp. *carota*	Apiaceae	H bienn	Cosmop.	1					1	8	6	5	4	5	4	0	EP − AC + ZC1
95	*Daucus pumilus* (L.) Hoffmanns. & Link	Apiaceae	T scap	Steno-Medit.	1	1	1	1		1	11	10	4	2	2	1	1	EP − AC + ZC1
96	*Delphinium halteratum* Sm. subsp. *halteratum*	Ranunculaceae	T scap	Steno-Medit.	1					1	8	9	4	3	4	2	0	EP − AC
97	*Dittrichia viscosa* (L.) Greuter subsp. *viscosa*	Asteraceae	H scap	Wide-Medit.	1					1	11	8	5	3	7	9	0	EP − AC
98	*Ecballium elaterium* (L.) A.Rich.	Cucurbitaceae	T scap	Wide-Medit.	1	1		1		1	7	8	5	3	5	3	0	EP − AUC1
99	*Echinophora spinosa* L.	Apiaceae	H scap	Wide-Medit.		1	1				12	8	5	4	7	1	2	EP − AC
100	*Echium arenarium* Guss.	Boraginaceae	H bienn	Steno-Medit.	1	1				1	11	10	4	2	3	1	0	EP − AC + BC
101	*Echium italicum* L. subsp. *siculum* (Lacaita) Greuter & Burdet	Boraginaceae	H bienn	Endem. Sic.	1					1	10	8	5	3	3	4	0	EP − AC + BC
102	*Echium parviflorum* Moench	Boraginaceae	H bienn	Steno-Medit.	1	1				1	11	10	4	2	3	1	0	EP − AC + BC
103	*Echium plantagineum* L.	Boraginaceae	H bienn	Wide-Medit.	1	1				1	10	8	5	3	5	5	0	EP − AC + BC
104	*Erigeron bonariensis* L.	Asteraceae	T scap	Americ.	1					1	8	8	5	3	X	7	0	EP − AC
105	*Erodium alnifolium* Guss.	Geraniaceae	T scap	W-Medit.		1					7	8	4	3	5	3	0	EP − BC
106	*Erodium chium* (L.) Willd.	Geraniaceae	H scap	Wide-Medit.		1					11	9	5	2	5	2	0	EP − BC
107	*Erodium cicutarium* (L.) L’Hér.	Geraniaceae	T caesp	Subcosmop.	1					1	8	7	5	3	5	3	0	EP − ZC1 + AUC1 + AUC2
108	*Erodium laciniatum* (Cav.) Willd. subsp. *laciniatum*	Geraniaceae	T scap	Steno-Medit.	1	1	1			1	11	9	4	2	7	2	1	EP − BC
109	*Erodium malacoides* (L.) L’Hér. subsp. *malacoides*	Geraniaceae	H bienn	W-Medit.	1	1				1	10	9	4	2	5	2	0	EP − BC
110	*Ervum tetraspermum* L.	Fabaceae	T scap	Cosmop.	1					1	6	5	5	5	3	4	0	EP − BC + AUC1
111	*Eryngium campestre* L.	Apiaceae	H scap	Wide-Medit.	1					1	9	7	5	3	8	3	0	EP − AC
112	*Eryngium maritimum* L.	Apiaceae	G rhiz	Medit.-Atl.			1				11	8	3	4	7	1	1	EP − AC + BC
113	*Euphorbia exigua* L. subsp. *exigua*	Euphorbiaceae	T scap	Wide-Medit.	1					1	10	9	5	2	6	1	0	EP − AUC1 + ZC3
114	*Euphorbia helioscopia* L. subsp. *helioscopia*	Euphorbiaceae	T scap	Cosmop.	1					1	9	7	5	3	5	6	0	EP − AUC1 + ZC3
115	*Euphorbia paralias* L.	Euphorbiaceae	Ch frut	Medit.-Atl.	1	1	1	1		1	11	8	5	1	X	1	2	EP − AUC1 + ZC3
116	*Euphorbia peplis* L.	Euphorbiaceae	T rept	Wide-Medit.	1			1		1	11	7	2	1	X	1	2	EP − AUC1 + ZC3
117	*Euphorbia peplus* L.	Euphorbiaceae	T scap	Cosmop.	1	1				1	6	7	4	4	5	7	0	EP − AUC1 + ZC3
118	*Euphorbia terracina* L.	Euphorbiaceae	H scap	Steno-Medit.	1	1		1		1	11	9	4	2	3	2	0	EP − AUC1 + ZC3
120	*Festuca fasciculata* Forssk.	Poaceae	T caesp	Medit.-Atl.	1	1	1		1	1	11	10	3	1	X	1	1	AP − AC
121	*Festuca myuros* L.	Poaceae	T scap	Subcosmop.	1						8	9	5	2	6	2	0	AP − AC
122	*Filago germanica* (L.) Huds.	Asteraceae	T scap	Paleotemp.		1	1				8	7	5	3	4	2	0	EP − AC
123	*Filago pygmaea* L.	Asteraceae	T rept	Steno-Medit.	1	1	1		1	1	11	9	4	1	3	1	0	EP − AC
124	*Filago pyramidata* L.	Asteraceae	T scap	Wide-Medit.	1					1	8	7	5	3	4	1	0	EP − AC
125	*Foeniculum vulgare* Mill. subsp. *piperitum* (Ucria) Bég.	Apiaceae	H scap	S-Medit.					1		9	8	5	3	7	7	0	EP − AC + BC
126	*Frankenia hirsuta* L.	Frankeniaceae	Ch suffr	Steno-Medit.-African	1	1		1		1	11	10	4	1	7	1	3	EP − AUC1
127	*Frankenia pulverulenta* L. subsp. *pulverulenta*	Frankeniaceae	T scap	Steno-Medit.-Asiatic	1					1	11	9	4	1	7	1	2	EP − AUC1
128	*Fumaria flabellata* Gasp.	Papaveraceae	T scap	Steno-Medit.	1	1				1	7	9	4	2	5	2	0	EP − AC + ZC3
129	*Galactites tomentosus* Moench	Asteraceae	H bienn	Steno-Medit.	1	1	1		1	1	8	8	4	3	X	7	0	EP − AC
130	*Galium murale* (L.) All.	Rubiaceae	T scap	Steno-Medit.	1	1				1	10	9	4	2	X	1	0	AP + EP − AC
131	*Galium verrucosum* Huds. subsp. *verrucosum*	Rubiaceae	T scap	Steno-Medit.	1	1				1	11	9	4	2	X	1	0	AP + EP − AC + ZC1
132	*Gastridium ventricosum* (Gouan) Schinz & Thell.	Poaceae	T scap	Medit.-Atl.	1					1	8	9	4	2	4	2	0	AP − AC
133	*Geranium dissectum* L.	Geraniaceae	T scap	Cosmop.	1					1	7	8	5	2	5	2	0	EP − AUC1
134	*Geranium molle* L.	Geraniaceae	H bienn	Subcosmop.	1	1				1	7	6	5	3	5	4	0	EP − AUC1
135	*Gladiolus italicus* Mill.	Iridaceae	G bulb	Wide-Medit.	1	1				1	9	9	5	3	5	3	0	EP − AUC1 + ZC3
136	*Glaucium flavum* Crantz	Papaveraceae	H scap	Wide-Medit.	1	1		1	1	1	11	9	5	1	4	1	1	EP − AUC1
137	*Halimione portulacoides* (L.) Aellen	Chenopodiaceae	Ch frut	Circumbor.	1			1		1	11	9	4	2	6	7	3	AP − AC
138	*Hedypnois rhagadioloides* (L.) F.W.Schmidt	Asteraceae	T scap	Steno-Medit.	1					1	11	9	4	3	7	2	0	EP − AC + BC
139	*Heliotropium europaeum* L.	Heliotropiaceae	T scap	Medit.-Turan.	1	1				1	10	8	5	3	7	2	1	EP − AC + BC
140	*Herniaria glabra* L. subsp. *glabra*	Caryophyllaceae	T scap	Paleotemp.		1					9	5	5	4	2	2	0	EP − AC
141	*Herniaria hirsuta* L. subsp. *hirsuta*	Caryophyllaceae	H caesp	Paleotemp.		1					9	6	5	4	2	2	0	EP − AC
142	*Hirschfeldia incana* (L.) Lagr.-Foss. subsp. *incana*	Brassicaceae	H scap	Medit.-Atl.	1	1				1	9	9	5	3	3	2	0	EP − AC
143	*Hordeum bulbosum* L.	Poaceae	H caesp	Paleosubtrop.		1					8	10	5	4	5	4	0	AP − AUC2
144	*Hordeum murinum* L. subsp. *leporinum* (Link) Arcang.	Poaceae	T scap	Wide-Medit.	1					1	8	8	4	5	5	3	0	AP − AUC2
145	*Hordeum murinum* L. subsp. *murinum*	Poaceae	T scap	Circumbor.			1				8	8	4	5	5	3	0	AP − AUC2
146	*Hyoscyamus albus* L.	Solanaceae	H bienn	Wide-Medit.	1	1		1		1	8	8	5	2	X	9	0	EP − AC
147	*Hyoseris scabra* L.	Asteraceae	T ros	Steno-Medit.	1	1	1			1	11	9	3	1	7	1	0	EP − AC
148	*Hyparrhenia hirta* (L.) Stapf subsp. *hirta*	Poaceae	H caesp	Paleotrop.	1				1	1	11	12	5	2	7	3	0	AP − AC
149	*Hypericum perfoliatum* L.	Hypericaceae	H scap	Steno-Medit.	1			1		1	6	8	4	4	3	4	0	EP − AC + ZC
150	*Hypericum perforatum* L. subsp. *perforatum*	Hypericaceae	H caesp	Cosmop.	1	1			1		7	8	6	X	X	X	0	EP − AC + ZC
151	*Hypericum triquetrifolium* Turra	Hypericaceae	H scap	E-Medit.						1	8	8	5	3	7	2	0	EP − AC + ZC
152	*Hypochaeris achyrophorus* L.	Asteraceae	T scap	Steno-Medit.	1		1			1	10	9	4	2	X	2	0	EP − AC
153	*Juncus acutus* L. subsp. *acutus*	Juncaceae	H caesp	Wide-Medit.	1	1				1	11	8	3	8	8	3	3	AP − AC
154	*Juno planifolia* (Mill.) Asch.	Iridaceae	G bulb	S-Medit.	1					1	8	8	3	3	4	2	0	EP − AUC1 + ZC3
155	*Koeleria splendens* C.Presl **	Poaceae	H caesp	Orof. S-Europ.		1					11	7	6	3	7	1	0	AP − AC
156	*Lagurus ovatus* L. subsp. *nanus* (Guss.) Messeri	Poaceae	T scap	Steno-Medit.	1					1	8	9	5	3	X	2	1	AP − AC
157	*Lagurus ovatus* L. subsp. *ovatus*	Poaceae	T scap	Wide-Medit.	1	1	1	1		1	8	9	5	3	X	2	1	AP − AC
158	*Lathyrus clymenum* L.	Fabaceae	T scap	Steno-Medit.	1	1					7	8	4	4	3	3	0	EP − BC
159	*Lemna minor* L.	Araceae	I nat	Subcosmop.						1	7	X	5	12	X	X	0	HP + EP − HC1
160	*Limbarda crithmoides* (L.) Dumort. subsp. *longifolia* (Arcang.) Greuter	Asteraceae	Ch suffr	Medit.-Atl.	1					1	11	8	4	7	9	5	3	EP − AC
161	*Limonium hyblaeum* Brullo *	Plumbaginaceae	H ros	Endem. Sic.	1			1		1	X	X	X	X	X	X	X	EP − AC
162	*Limonium narbonense* Mill.	Plumbaginaceae	H ros	Wide-Medit.	1					1	11	7	5	6	7	5	3	EP − AC
163	*Limonium sinuatum* (L.) Mill.	Plumbaginaceae	H scap	S-Medit.	1	1	1	1		1	11	11	4	1	7	2	1	EP − AC
164	*Limonium syracusanum* Brullo *	Plumbaginaceae	Ch sufrr	Endem. Sic.	1						X	X	X	X	X	X	X	EP − AC
165	*Limonium virgatum* (Willd.) Fourr.	Plumbaginaceae	H ros	Wide-Medit.	1	1	1	1		1	9	9	3	1	9	1	3	EP − AC
166	*Linaria reflexa* (L.) Desf. subsp. *reflexa*	Plantaginaceae	T rept	SW-Medit.	1					1	7	8	5	3	5	3	0	EP − AC
167	*Linum strictum* L.	Linaceae	T scap	Steno-Medit.	1					1	11	9	4	2	5	2	0	EP − AC + ZC
168	*Linum trigynum* L.	Linaceae	T scap	Wide-Medit.	1					1	10	9	5	2	3	2	0	EP − AC + ZC
169	*Lithospermum officinale* L.	Boraginaceae	H scap	Eurosiber.		1					6	X	5	X	8	6	0	EP − BC + ZC
170	*Lobularia maritima* (L.) Desv.	Brassicaceae	H scap	Steno-Medit.	1	1		1	1	1	8	9	4	2	X	1	0	EP − AC
171	*Logfia gallica* (L.) Cosson & Germ.	Asteraceae	T scap	Wide-Medit.	1					1	10	8	5	2	3	1	0	EP − AC
172	*Loncomelos narbonensis* (L.) Raf.	Asparagaceae	G bulb	Wide-Medit.	1	1				1	8	7	5	4	6	4	0	EP − AUC1 + ZC3
173	*Lotus creticus* L.	Fabaceae	Ch suffr	Steno-Medit.	1	1	1	1	1	1	11	10	3	1	X	1	2	EP − BC
174	*Lotus cytisoides* L.	Fabaceae	Ch suffr	Steno-Medit.	1					1	11	10	3	1	X	1	2	EP − BC
175	*Lotus edulis* L.	Fabaceae	T scap	Steno-Medit.	1	1			1	1	9	8	4	2	5	3	0	EP − BC
176	*Lotus ornithopodioides* L.	Fabaceae	T scap	Steno-Medit.	1					1	10	9	4	2	1	1	0	EP − BC
177	*Lotus tetragonolobus* L.	Fabaceae	T scap	Steno-Medit.	1					1	8	6	4	6	9	6	0	EP − BC
178	*Lysimachia arvensis* (L.) U. Manns & Anderb. subsp. *arvensis*	Primulaceae	T rept	Wide-Medit.	1					1	6	6	5	5	X	6	0	EP − AUC1
179	*Lysimachia foemina* (Mill.) U. Manns & Anderb.	Primulaceae	T rept	Subcosmop.		1					8	7	5	4	9	5	0	EP − AUC1
180	*Lythrum hyssopifolia* L.	Lythraceae	T scap	Subcosmop.	1					1	8	7	5	7	3	4	0	EP − AC + HC + ZC
181	*Malva cretica* Cav. subsp. *cretica*	Malvaceae	T scap	Steno-Medit.	1					1	7	7	4	4	7	8	0	EP − AC + BC
182	*Malva sylvestris* L.	Malvaceae	H scap	Subcosmop.	1					1	8	6	4	4	X	8	0	EP − AC + BC
183	*Mandragora autumnalis* Bertol.	Solanaceae	H ros	Steno-Medit.	1	1		1		1	7	9	4	2	7	3	0	EP − ZC2
184	*Marrubium vulgare* L.	Lamiaceae	H scap	Cosmop.	1	1		1		1	9	8	5	3	8	8	0	EP − AC + ZC
185	*Matthiola tricuspidata* (L.) R.Br.	Brassicaceae	T scap	Steno-Medit.	1			1		1	11	10	4	1	3	1	1	EP − AC
186	*Medicago littoralis* Rohde ex Loisel.	Fabaceae	T scap	Wide-Medit.	1		1		1	1	11	9	5	2	X	2	0	EP − ZC1
187	*Medicago minima* (L.) L.	Fabaceae	T scap	Wide-Medit.-Asiatic	1				1	1	10	7	5	3	8	1	0	EP − ZC1
188	*Medicago orbicularis* (L.) Bartal.	Fabaceae	T scap	Wide-Medit.	1				1	1	7	8	5	3	4	4	0	EP − BC
189	*Medicago polymorpha* L.	Fabaceae	T scap	Subcosmop.	1					1	9	9	5	2	X	2	0	EP − ZC1
190	*Medicago truncatula* Gaertn.	Fabaceae	T scap	Steno-Medit.	1					1	11	8	4	1	X	1	1	EP − ZC1
191	*Melica ciliata* L. subsp. *ciliata*	Poaceae	H caesp	Wide-Medit.		1					8	7	5	2	7	2	0	AP − AC + ZC3
192	*Melica ciliata* L. subsp. *magnolii* (Godr. & Gren.) K.Richt.	Poaceae	H caesp	Medit.-Turan.	1	1				1	8	7	5	2	7	2	0	AP − AC + ZC3
193	*Melica minuta* L. subsp. *latifolia* (Coss.) W.Hempel	Poaceae	H caesp	Steno-Medit.	1					1	8	9	4	2	5	2	0	AP − AC + ZC3
194	*Mentha pulegium* L.	Lamiaceae	H scap	Subcosmop.		1					8	7	5	7	X	2	0	EP − AC
195	*Mercurialis annua* L.	Euphorbiaceae	T scap	Paleotemp.	1	1				1	7	7	5	4	7	8	0	AP − AUC1 + ZC3
196	*Mesembryanthemum nodiflorum* L.	Aizoaceae	T scap	S-Medit.-African	1	1	1			1	11	12	5	1	X	1	2	EP − HC1
197	*Micromeria graeca* (L.) Benth. ex Rchb. subsp. *graeca*	Lamiaceae	Ch suffr	Steno-Medit.	1					1	8	8	4	2	X	2	0	EP − AC
198	*Moraea sisyrinchium* (L.) Ker Gawl.	Iridaceae	G bulb	Steno-Medit.	1			1		1	11	9	3	2	4	1	0	EP − AUC1 + ZC3
199	*Narcissus miniatus* Donn.-Morg., Koop. and Zonn.	Amaryllidaceae	G bulb	Steno-Medit.	1					1	8	7	4	4	5	4	0	EP − AUC1
200	*Nigella damascena* L.	Ranunculaceae	T scap	Wide-Medit.	1	1			1	1	8	9	5	3	4	2	0	EP − AUC1
201	*Olea europaea* L. var. *sylvestris* (Mill.) Hegi	Oleaceae	P caesp	Steno-Medit.	1	1			1	1	11	10	4	1	X	2	0	AP + EP − ZC2
202	*Oloptum miliaceum* (L.) Röser and H.R.Hamasha	Poaceae	H caesp	Medit.-Turan.	1	1			1	1	5	7	4	4	7	5	0	AP − AC
203	*Onobrychis caput-galli* (L.) Lam.	Fabaceae	T scap	Steno-Medit.	1				1	1	11	9	4	2	7	1	0	EP − BC + ZC1
204	*Ononis natrix* L. subsp. *ramosissima* (Desf.) Batt.	Fabaceae	H caesp	Wide-Medit.	1	1	1	1	1	1	X	X	X	X	X	X	X	EP − BC
205	*Ononis variegata* L.	Fabaceae	T scap	Steno-Medit.	1	1	1			1	11	9	4	2	X	1	0	EP − BC
206	*Ophrys lutea* Cav.	Orchidaceae	G bulb	Steno-Medit.	1					1	8	9	4	3	6	3	0	EP − AC
207	*Ophrys sicula* Tineo	Orchidaceae	G bulb	Subendem.	1					1	8	9	4	3	6	3	0	EP − AC
208	*Ophrys speculum* Link	Orchidaceae	G bulb	W-Medit.		1					8	9	4	3	6	3	0	EP − AC
209	*Orchis italica* Poir.	Orchidaceae	G bulb	Steno-Medit.	1					1	8	9	4	2	4	2	0	EP − AC
210	*Ornithogalum gussonei* Ten.	Asparagaceae	G bulb	Steno-Medit.	1					1	7	7	4	2	6	2	0	EP − AUC1 + ZC3
211	*Orobanche minor* Sm.	Orobanchaceae	T par	Subcosmop.	1					1	7	6	5	4	5	4	0	EP − AC + AUC1
212	*Oxalis pes-caprae* L.	Oxalidaceae	G bulb	African	1					1	8	10	4	3	X	5	0	EP + CG − ATC
213	*Pallenis spinosa* (L.) Cass. subsp. *spinosa*	Asteraceae	T scap	Wide-Medit.					1		11	9	5	4	X	7	0	EP − AC
214	*Pancratium maritimum* L.	Amaryllidaceae	G bulb	Steno-Medit.	1	1	1	1		1	11	10	3	1	X	1	0	EP − AUC1 + HC2 + ZC2
215	*Papaver hybridum* L.	Papaveraceae	T scap	Medit.-Turan.	1					1	11	8	6	2	3	2	0	EP − AC
216	*Papaver rhoeas* L. subsp. *rhoeas*	Papaveraceae	T scap	E-Medit.	1					1	6	6	5	5	7	X	0	EP − AC
217	*Papaver somniferum* L.	Papaveraceae	T scap	Subcosmop.	1	1				1	X	X	X	X	X	X	X	EP − AC
218	*Parapholis incurva* (L.) C.E.Hubb.	Poaceae	T scap	Medit.-Atl.	1					1	11	7	4	5	7	2	3	AP − AC
219	*Parapholis strigosa* (Dumort.) C.E.Hubb.	Poaceae	T scap	Medit.-Atl.		1	1				11	7	3	5	7	2	2	AP − AC
220	*Parietaria judaica* L.	Urticaceae	H scap	Medit.-Atl.	1					1	7	8	5	3	X	6	0	BP − AC
221	*Parietaria lusitanica* L. subsp. *lusitanica*	Urticaceae	T rept	Steno-Medit.		1					7	10	4	3	4	6	0	BP − AC
222	*Phagnalon saxatile* (L.) Cass.	Asteraceae	Ch suffr	W-Medit.	1					1	7	9	4	2	X	1	0	EP − AC + HC1
223	*Phalaris paradoxa* L.	Poaceae	T scap	Steno-Medit.	1					1	7	7	X	4	6	4	0	AP − AC
224	*Phedimus stellatus* (L.) Raf.	Crassulaceae	T scap	Steno-Medit.	1	1				1	7	9	4	2	2	2	0	EP − AC
225	*Phillyrea latifolia* L.	Oleaceae	P caesp	W-Medit.	1					1	5	8	4	4	X	5	0	AP + EP − ZC2
226	*Pistacia lentiscus* L.	Anacardiaceae	P caesp	Steno-Medit.	1	1	1	1	1	1	10	10	5	2	X	2	0	AP − BC
227	*Plantago afra* L. subsp. *afra*	Plantaginaceae	T scap	Steno-Medit.	1						11	6	4	3	7	2	0	AP + EP − AC + ZC
228	*Plantago coronopus* L.	Plantaginaceae	T scap	Wide-Medit.	1	1	1				8	7	5	7	7	4	0	AP − EP + HC1
229	*Plantago lagopus* L.	Plantaginaceae	T scap	Steno-Medit.	1				1		11	9	4	3	3	1	0	AP + EP − AC + ZC
230	*Plantago serraria* L.	Plantaginaceae	H ros	Steno-Medit.	1	1			1	1	11	10	4	2	7	1	0	AP + EP − BC + ZC
231	*Plantago weldenii* Rchb.	Plantaginaceae	T scap	Wide-Medit.	1				1	1	X	X	X	X	X	X	X	AP + EP − AC + ZC
232	*Polycarpon tetraphyllum* (L.) L. subsp. *alsinifolium* (Biv.) Ball	Caryophyllaceae	T scap	S-Medit.	1					1	7	7	5	4	5	6	0	EP − AC
233	*Polycarpon tetraphyllum* (L.) L. subsp. *diphyllum* (Cav.) O.Bolòs & Font Quer	Caryophyllaceae	T scap	Steno-Medit.	1					1	7	8	4	4	5	6	0	EP − AC
234	*Polygonum maritimum* L.	Polygonaceae	H rept	Subcosmop.	1					1	11	10	4	1	3	1	2	AP + EP − AC
235	*Polypogon maritimus* Willd. subsp. *maritimus*	Poaceae	T scap	Steno-Medit.		1				1	8	8	4	7	9	6	1	AP − AC
236	*Polypogon monspeliensis* (L.) Desf.	Poaceae	T scap	Paleosubtrop.	1	1	1		1	1	8	8	5	9	8	6	0	AP − AC
237	*Polypogon subspathaceus* Req.	Poaceae	T scap	Steno-Medit.	1	1				1	8	8	4	8	8	6	0	AP − AC
238	*Portulaca oleracea* L.	Portulacaceae	T scap	Subcosmop.	1					1	7	8	5	4	7	7	0	AP + EP − BC
239	*Poterium sanguisorba* L.	Rosaceae	H scap	Paleotemp.	1					1	7	6	5	3	8	2	0	AP − AC
240	*Poterium spinosum* L.	Rosaceae	NP	Steno-Medit.	1	1	1	1	1	1	12	11	4	2	X	2	0	AP − ZC2
241	*Prangos ferulacea* (L.) Lindl. **	Apiaceae	H scap	Medit.-Turan.					1		11	8	3	2	7	2	0	EP − BC
242	*Ranunculus bullatus* L.	Ranunculaceae	H ros	Steno-Medit.	1					1	7	8	4	2	7	2	0	EP − HC1
244	*Reichardia intermedia* (Sch. Bip.) Samp.	Asteraceae	T scap	Steno-Medit.	1						X	X	X	X	X	X	X	EP − AC
243	*Reichardia picroides* (L.) Roth	Asteraceae	H scap	Steno-Medit.	1				1	1	7	8	4	3	6	2	0	EP − AC
245	*Rhagadiolus stellatus* (L.) Gaertn.	Asteraceae	T scap	Wide-Medit.					1		8	9	5	3	7	2	0	EP − AC + ZC1
246	*Rostraria cristata* (L.) Tzvelev	Poaceae	T caesp	Subcosmop.	1	1				1	7	5	5	6	8	2	0	AP − AC
247	*Rubia peregrina* L.	Rubiaceae	P lian	W-Medit.	1					1	5	9	4	4	5	3	0	EP − ZC2
248	*Rumex bucephalophorus* L. subsp. *bucephalophorus*	Polygonaceae	T scap	Steno-Medit.	1	1				1	8	12	5	2	2	1	0	AP − AC
249	*Rumex intermedius* DC.	Polygonaceae	H scap	W-Medit.		1					8	7	5	3	7	5	0	AP − AC
250	*Rumex pulcher* L. subsp. *pulcher*	Polygonaceae	H scap	Wide-Medit.	1	1				1	8	8	5	2	6	9	0	AP − AC
251	*Rumex spinosus* L.	Polygonaceae	T scap	W-Medit.	1	1				1	11	12	5	2	3	2	0	AP + EP − AC
252	*Rumex thyrsoides* Desf.	Polygonaceae	H scap	W-Medit.	1					1	8	8	4	3	7	5	0	AP − AC
253	*Saccharum biflorum* Forssk.	Poaceae	H caesp	Paleotrop.	1			1		1	X	X	X	X	X	X	X	AP − X
254	*Sagina apetala* Ard. subsp. *apetala*	Caryophyllaceae	T scap	Wide-Medit.	1					1	8	7	5	6	4	5	0	EP − AC + ZC3
255	*Sagina maritima* Don	Caryophyllaceae	T scap	Medit.-Atl.	1	1				1	8	X	X	7	X	X	0	EP − AC + ZC3
256	*Salsola tragus* L.	Chenopodiaceae	T scap	Paleotemp.	1		1	1		1	X	X	X	X	X	X	X	AP − AC
257	*Salvia clandestina* L.	Lamiaceae	H scap	Steno-Medit.	1	1	1			1	8	8	4	3	5	7	0	EP − AC
258	*Sclerochloa dura* (L.) P.Beauv.	Poaceae	T scap	Wide-Medit.					1		8	8	5	2	5	2	0	AP − AC
259	*Scolymus grandiflorus* Desf.	Asteraceae	H scap	SW-Medit.		1	1	1			11	10	3	2	5	3	0	EP − AC
260	*Scolymus hispanicus* L. subsp. *hispanicus*	Asteraceae	H bienn	Wide-Medit.	1	1	1		1	1	11	8	5	3	X	2	0	EP − AC
261	*Scorpiurus subvillosus* L.	Fabaceae	T scap	Wide-Medit.	1				1	1	X	X	X	X	X	X	X	EP − BC + ZC1
262	*Scrophularia peregrina* L.	Scrophulariaceae	T scap	Steno-Medit.		1					5	8	4	4	6	5	0	EP − AC
263	*Sedum caeruleum* L.	Crassulaceae	T scap	SW-Medit.				1			10	10	3	1	1	1	0	EP − AC
264	*Sedum rubens* L.	Crassulaceae	T scap	Medit.-Atl.	1					1	9	8	3	2	X	2	0	EP − AC
265	*Senecio glaucus* L. subsp. *coronopifolius* (Maire) C.Alexander	Asteraceae	T scap	Saharo-Sind.	1	1				1	11	12	6	1	6	1	0	EP − AC
266	*Senecio pygmaeus* DC.	Asteraceae	T scap	Endem. Sic.-Malta.	1	1				1	9	10	3	1	3	1	0	EP − AC
267	*Senecio vulgaris* L. subsp. *vulgaris*	Asteraceae	T scap	Cosmop.	1	1				1	7	X	X	5	X	8	0	EP − AC
268	*Serapias parviflora* Parl.	Orchidaceae	G bulb	W-Medit.	1					1	11	10	4	2	4	2	0	EP − AC
269	*Seseli tortuosum* L. subsp. *maritimum* C. Brullo, Brullo, Giusso & Sciandr.	Apiaceae	H bienn	Endem. Ital.	1					1	11	10	4	3	7	2	0	EP − AC
270	*Silene colorata* Poir.	Caryophyllaceae	T scap	Steno-Medit.	1	1			1	1	11	9	3	1	X	1	2	EP − AC + ZC3
271	*Silene niceensis* All.	Caryophyllaceae	T scap	Steno-Medit.	1	1	1			1	11	10	4	2	3	1	1	EP − AC + ZC3
272	*Silene sedoides* Poir. subsp. *sedoides*	Caryophyllaceae	T scap	Steno-Medit.	1	1				1	11	10	3	2	2	1	1	EP − AC + ZC3
273	*Silene vulgaris* (Moench) Garcke subsp. *tenoreana* (Colla) Soldano & F.Conti	Caryophyllaceae	H scap	E-Medit.	1	1				1	8	X	X	4	7	2	0	EP − AC + ZC3
274	*Sixalix atropurpurea* (L.) Greuter & Burdet	Dipsacaceae	H bienn	Steno-Medit.	1				1	1	6	8	4	3	X	2	0	EP − AC + ZC
275	*Smilax aspera* L.	Smilacaceae	G rhiz	Paleosubtrop.	1				1	1	6	10	4	2	5	3	0	AP + EP − ZC2
276	*Solanum dulcamara* L.	Solanaceae	NP	Paleotemp.		1					7	5	X	8	X	8	0	EP − ZC2
277	*Solanum linnaeanum* Hepper & P.-M.L.Jaeger	Solanaceae	NP	African		1					9	11	5	2	X	1	0	EP − ZC2
278	*Solanum nigrum* L.	Solanaceae	T scap	Cosmop.	1	1				1	7	6	5	3	5	7	0	EP − ZC2
279	*Sonchus asper* (L.) Hill subsp. *asper*	Asteraceae	T scap	Cosmop.	1					1	7	5	X	4	7	7	0	EP − AC
280	*Sonchus bulbosus* (L.) N.Kilian & Greuter subsp. *bulbosus*	Asteraceae	G bulb	Steno-Medit.	1	1				1	7	8	4	3	5	3	0	EP − AC
281	*Sonchus oleraceus* L.	Asteraceae	T scap	Cosmop.	1					1	7	5	X	4	8	8	0	EP − AC
282	*Sonchus tenerrimus* L.	Asteraceae	T scap	Steno-Medit.	1					1	7	8	4	2	5	4	0	EP − AC
285	*Spergularia bocconei* (Scheele) Graebn.	Caryophyllaceae	T scap	Subcosmop.	1						7	7	X	6	3	4	0	EP − AC + ZC3
283	*Spergularia heldreichii* E.Simon & P.Monnier	Caryophyllaceae	T scap	Steno-Medit.	1					1	11	9	3	2	2	1	2	EP − AC + ZC3
284	*Spergularia marina* (L.) Besser	Caryophyllaceae	T scap	Subcosmop.	1		1			1	7	7	5	6	8	X	2	EP − AC + ZC3
286	*Sporobolus virginicus* (L.) Kunth	Poaceae	G rhiz	Subtrop.	1	1	1	1			11	11	4	1	X	1	3	AP − AC
287	*Stachys major* (L.) Bartolucci & Peruzzi	Lamiaceae	Ch frut	Steno-Medit.	1	1		1		1	11	10	4	2	X	1	0	EP − AC
288	*Stachys romana* (L.) E.H.L.Krause	Lamiaceae	T scap	Steno-Medit.	1	1	1			1	11	9	4	2	6	1	0	EP − AC
289	*Stipellula capensis* (Thunb.) Röser & H.R.Hamasha	Poaceae	T scap	Steno-Medit.	1				1	1	11	10	4	1	4	1	0	AP − AC
290	*Suaeda maritima* (L.) Dumort.	Chenopodiaceae	T scap	Cosmop.				1			9	6	2	8	7	7	3	AP − AC
291	*Suaeda vera* J.F.Gmel.	Chenopodiaceae	NP	Cosmop.	1	1				1	11	10	5	8	9	7	3	AP − AC
292	*Sulla spinosissima* (L.) B.H.Choi & H.Ohashi	Fabaceae	T scap	W-Medit.	1					1	11	9	4	2	3	2	0	EP − ZC1
293	*Symphyotrichum squamatum* (Spreng.) G.L.Nesom	Asteraceae	T scap	Neotrop.	1					1	8	8	5	4	7	7	0	EP − AC
294	*Teucrium fruticans* L. subsp. *fruticans*	Lamiaceae	NP	W-Medit.	1			1	1	1	11	8	4	2	7	2	0	EP − AC
295	*Thapsia garganica* L. subsp. *garganica*	Apiaceae	H scap	S-Medit.	1	1	1		1	1	11	8	5	3	5	3	0	EP − AC + ZC1
296	*Theligonum cynocrambe* L.	Rubiaceae	T scap	Steno-Medit.	1					1	10	9	4	2	3	4	0	EP − AC
297	*Thinopyrum junceum* (L.) Á.Löve	Poaceae	G rhiz	Wide-Medit.				1			12	6	5	7	7	7	2	AP − AC
298	*Thymelaea hirsuta* (L.) Endl.	Thymelaeaceae	Ch suffr	S-Medit.-W-Asiatic	1	1		1		1	11	8	5	2	X	3	1	EP − AUC1
299	*Thymelaea passerina* (L.) Coss. & Germ.	Thymelaeaceae	T scap	Wide-Medit.-Asiatic		1					8	7	5	3	7	2	0	EP − AUC1
300	*Tordylium apulum* L.	Apiaceae	T scap	Steno-Medit.	1					1	11	9	4	2	X	2	0	EP − AC
301	*Trifolium angustifolium* L. subsp. *angustifolium*	Fabaceae	T scap	Wide-Medit.					1		10	9	5	2	3	2	0	EP − AC
302	*Trifolium campestre* Schreb.	Fabaceae	T scap	Paleotemp.	1					1	8	5	5	4	X	3	0	EP − AC
303	*Trifolium nigrescens* Viv. subsp. *nigrescens*	Fabaceae	T scap	Wide-Medit.	1					1	8	6	5	5	5	6	0	EP − AC
304	*Trifolium scabrum* L.	Fabaceae	T rept	Wide-Medit.	1					1	10	8	5	2	9	1	0	EP − AC
305	*Trifolium squarrosum* L.	Fabaceae	T scap	Wide-Medit.					1		10	9	5	2	3	2	0	EP − AC
306	*Trifolium stellatum* L.	Fabaceae	T scap	Wide-Medit.	1					1	10	9	5	2	X	2	0	EP − AC
307	*Trifolium tomentosum* L.	Fabaceae	T rept	Paleotemp.	1					1	9	9	5	4	7	2	0	EP − AC
308	*Triglochin laxiflora* Guss.	Juncaginaceae	G bulb	W-Medit.	1					1	8	8	4	8	7	7	0	AP − AUC1
309	*Trigonella foenum-graecum* L.	Fabaceae	T scap	W-Asiatic					1		9	9	5	2	X	4	0	EP − AC
310	*Tripodion tetraphyllum* (L.) Fourr.	Fabaceae	T scap	Steno-Medit.	1					1	10	10	3	2	X	3	0	EP − AC
311	*Tuberaria guttata* (L.) Fourr.	Cistaceae	T scap	Wide-Medit.					1		11	9	5	2	1	1	0	EP − BC
312	*Typha domingensis* (Pers.) Steud.	Typhaceae	G rhiz	Cosmop.	1					1	X	X	X	X	X	X	X	AP − AC
313	*Umbilicus horizontalis* (Guss.) DC.	Crassulaceae	G bulb	Medit.-Atl.		1					5	8	4	3	X	3	0	EP − AC
314	*Urospermum dalechampii* (L.) F.W.Schmidt	Asteraceae	H scap	Medit.-Atl.	1	1				1	8	8	5	3	X	3	0	EP − AC
315	*Urospermum picroides* (L.) Scop. ex F.W.Schmidt	Asteraceae	T scap	Wide-Medit.	1	1				1	11	9	5	2	X	2	0	EP − AC
316	*Urtica membranacea* Poir.	Urticaceae	T scap	S-Medit.	1	1				1	7	8	5	3	6	3	0	BP − AC
317	*Urtica urens* L.	Urticaceae	T scap	Subcosmop.		1					7	6	X	5	7	8	0	BP − AC
318	*Valantia muralis* L.	Rubiaceae	T scap	Steno-Medit.	1	1	1	1		1	10	9	4	2	3	1	0	EP − AC
319	*Valerianella microcarpa* Loisel.	Valerianaceae	T scap	Steno-Medit.	1	1				1	11	9	4	2	5	1	0	EP − AC
320	*Verbascum sinuatum* L.	Scrophulariaceae	H bienn	Wide-Medit.	1	1	1	1		1	9	8	5	3	7	7	0	EP − AC + ZC
321	*Verbascum thapsus* L. subsp. *thapsus*	Scrophulariaceae	H bienn	Europ.-Caucas.		1					8	X	4	4	7	7	0	EP − AC + ZC
322	*Vicia benghalensis* L.	Fabaceae	T scap	Steno-Medit.	1					1	11	9	4	2	5	2	0	EP − BC + AUC1
323	*Vicia bithynica* (L.) L.	Fabaceae	T scap	Wide-Medit.	1					1	7	7	5	3	5	5	0	EP − BC + AUC1
324	*Vicia leucantha* Biv.	Fabaceae	T scap	SW-Medit.						1	11	11	5	2	7	2	0	EP − BC + AUC1
325	*Vicia macrocarpa* (Moris) Bertol.	Fabaceae	T scap	Medit.-Turan.		1					5	5	6	X	X	X	0	EP − BC + AUC1
326	*Vicia sativa* L.	Fabaceae	T scap	Subcosmop.	1					1	5	5	6	X	X	X	0	EP − BC + AUC1
327	*Vicia villosa* Roth	Fabaceae	T scap	Steno-Medit.	1						7	6	5	4	4	5	0	EP − BC + AUC1
328	*Xanthium italicum* Moretti	Asteraceae	T scap	S-Europ.	1					1	8	7	5	5	X	1	0	EP − ZC1
				TOTAL	269	147	54	57	55	257								

* In the impossibility to check the exsiccata of Albo (stored in the Herbarium of the University of Naples “Federico II”, NAP), due to the restrictions imposed by the Sars-CoV-2, the interpretation of the plant names reported by Albo (1919) resulted sometimes difficult. For instance, Albo reported the local occurrence of eight different taxa belonging to the genus *Statice* (=*Limonium*). Apart from *Statice sinuata* (=*L. sinuatum*, still occurring on the islet), the other seven names probably correspond to three taxa only, i.e., the ones still occurring there. Based on morphological, ecological and occurrence data, in fact, the best match for Albo’s names is the following: *Statice psiloclada* var. *panormitana*, *St. psiloclada* var. *gracilis* and *St. psiloclada* var. *albida* probably correspond to *L. hyblaeum*, the records of *Statice minuta* var*. cosyrensis* and *St. minuta* var. *minutiflora* should instead be referred to *L. syracusanum*, while *St. minuta* var. *virgata* and *St. minuta* var. *dubia* may coincide with *L. virgatum*. ** *Koeleria splendens* and *Prangos ferulacea* were almost certainly recorded by mistake.

## Appendix B. Syntaxonomical Scheme of the Vegetation Units Recorded from the Island of Capo Passero (SE-Sicily)

Crithmo-Staticetea Br.-Bl. in Br.-Bl., Roussine and Nègre 1952
Crithmo-Staticetalia Molinier 1934
Crithmo-Staticion Molinier 1934
***Crithmo maritimi-Limonietum virgati*** Pirone 1995
***Limonietum hyblaei*** Bartolo, Brullo and Marcenò 1982
Salicornietea fruticosae Br.-Bl. and R. Tx. ex A. Bolòs y Vayreda and O. Bolòs in A. Bolòs y Vayreda 1950
Salicornietalia fruticosae Br.-Bl. 1933
Arthrocnemion macrostachyi Rivas-Mart. and M. Costa 1984
***Limonio virgati-Arthrocnemetum macrostachyi*** Biondi, Casavecchia and Guerra 2006
Suaedion brevifoliae Br.-Bl. and O. de Bolòs 1958
***Halimiono portulacoidis-Suaedetum******verae*** Molinier and Tallon 1970 corr. Géhu in Géhu and al. 1984
Saginetea maritimae Westhoff, Van Leeuwen and Adriani 1962
Frankenietalia pulverulentae Rivas-Mart. ex Castroviejo and Porta 1976
Frankenion pulverulentae Rivas-Mart. ex Castroviejo and Porta 1976
***Polypogonetum subspathacei*** Gamisans 1992
***Parapholido incurvae-Catapodietum balearici*** Rivas-Mart. et al. 1990 corr. Brullo and Giusso 2003
***Senecio pygmaeus*** community
Mesembryanthemion crystallini Rivas-Mart., Wildpret, Del Arco, O. Rodríguez, Pérez de Paz, García Gallo, Acebes, T.E. Díaz and Fernández-González 1993
***Spergulario bocconei-Mesembryanthemetum nodiflori*** Costa in Costa et al. 1997
Ononido-Rosmarinetea Br.-Bl. in A. Bolòs y Vayreda 1950
Cisto-Micromerietalia julianae Oberd. 1954
Cisto cretici-Ericion manipuliflorae Horvatic 1958
***Chamaeropo humilis-Sarcopoterietum spinosi*** Barbagallo, Brullo and Fagotto 1979
Quercetea ilicis Br.-Bl. ex A. Bolòs and O. de Bolòs in A. Bolòs y Vayreda 1950
Pistacio lentisci-Rhamnetalia alaterni Rivas-Mart. 1975
Oleo-Ceratonion siliquae Br.-Bl. ex Guinochet and Drouineau 1944
***Pistacio lentisci-Chamaeropetum humilis*** Brullo and Marcenò 1985
Ammophiletea Br.-Bl. et Tx. ex Westhoff et al. 1946
Ammophiletalia Br.-Bl. et Tx. ex Westhoff et al. 1946
Ammophilion australis Br.-Bl. 1921
***Medicagini marinae-Ammophiletum australis*** Br.-Bl. 1921 corr. Prieto and Diaz 1991
Crucianelletalia maritimae Sissing 1974
Crucianellion maritimae Rivas Goday and Rivas-Mart. 1958
***Centaureo sphaerocephalae-Ononidetum ramosissimae*** Br.-Bl. and Frei in Frei 1937
Cakiletea maritimae Tx. and Preising in Tx. Ex Br.-Bl. and Tx. 1952
Atriplicetalia littoralis Sissingh in Westhoff et al. 1946
Atriplicion littoralis Nordhagen 1940
***Salsolo-Cakiletum maritimae*** Costa and Mansanet 1981 corr. Rivas-Mart. et al. 1992
Helianthemetea guttati Rivas Goday and Rivas-Mart. 1963
Vulpietalia Pignatti 1953
Laguro ovati-Vulpion fasciculatae Géhu and Biondi 1994
***Silene nicaensis*** and ***Senecio coronopifolius*** community
Stipo-Trachynietea distachyae S. Brullo in S. Brullo et al. 2001
Stipo-Bupleuretalia semicompositi S. Brullo in S. Brullo et al. 2001
Plantagini-Catapodion marini S. Brullo 1985
***Stipellula capensis and Asteriscus aquaticus*** community
Phragmito-Magnocaricetea Klika in Klika and Novák 1941
Phragmitetalia Koch 1926
Phragmition Koch 1926
***Typhetum domingensis*** Brullo, Minissale and Spampinato 1994

## Appendix C

**Table A2:** Phytosociological relevés from the Island of Capo Passero (SE Sicily)—shrub vegetation.

Cluster	1	1	1	1	1	1	1	1	1	1	1	1	1	1	1	1	1	1	1	1	1	1	1	1	1	2	2	2	2	2	2	2	2	2	2	2	2	2	2	2	2	**Occurrences**
Relevé number	1	8	198	2	171	10	15	3	4	6	7	16	145	146	147	148	157	5	9	11	12	13	14	172	189	17	190	191	202	24	134	135	18	19	20	21	22	23	25	26	27	
Shrub layer (cover %)	99	60	60	80	70	95	55	95	95	99	70	80	85	90	85	90	100	95	70	90	98	95	70	80	90	85	100	100	100	85	100	90	98	98	98	55	90	90	90	90	80	
Herbaceous layer (cover %)	15	65	-	20	-	45	40	45	20	35	15	35	-	-	-	-	-	10	20	5	30	35	55	-	-	15	-	-	-	4	-	-	8	45	35	20	20	20	20	40	45	
Shrub layer—mean height (cm)	70	100	-	100	-	100	120	100	100	70	70	100	-	-	-	-	-	100	80	70	80	100	80	-	-	40	-	-	-	45	-	-	40	45	50	40	30	40	40	35	30	
Herbaceous layer—mean height (cm)	40	50	-	30	-	30	45	25	15	30	15	15	-	-	-	-	-	20	40	10	60	40	25	-	-	15	-	-	-	10	-	-	25	50	45	25	25	40	25	40	15	
Plot size (square m)	50	50	100	200	100	80	70	25	100	40	100	100	100	100	100	100	25	100	200	50	100	100	100	100	50	100	50	50	100	80	100	100	50	20	100	50	100	100	50	70	50	
Exposure	SW	-	-	-	-	-	-	WWN	-	-	-	-	S	S	-	-	-	-	S	-	-	-	-	-	-	-	-	-	-	-	S	S	-	-	-	S	-	-	W	NW	-	
Slope (°)	2	-	-	-	-	-	-	-	-	-	-	-	5	5	-	-	-	-	-	-	-	-	-	-	-	-	-	-	-	-	4	5	-	-	-	-	-	-	-	1	-	
Stones and rocks (cover %)	-	-	-	-	-	-	-	-	-	-	-	-	-	-	-	-	-	-	-	-	-	-	-	-	-	-	-	-	-	-	-	-	-	-	-	-	-	-	-	-	-	
**Char. Ass.**																																									
*Chamaerops humilis* L.	5	4	3	4	4	3	3	3	2	2	4	4	4	3	3	3	3	3	3	2	3	4	4	3	4	.	1	+	1	.	+	+	.	.	.	.	r	.	.	.	.	**31**
*Poterium spinosum* L.	.	1	.	.	.	.	.	.	.	.	.	.	.	.	.	2	1	3	3	4	4	1	3	2	2	5	5	5	5	5	4	4	5	5	5	3	5	5	5	5	5	**27**
**Char. Oleo-Ceratonion**																																									
*Pistacia lentiscus* L.	.	1	+	3	4	4	2	4	3	5	2	1	3	4	4	3	4	1	2	2	+	1	2	4	2	1	+	2	.	2	1	2	+	+	+	+	1	+	1	.	+	**38**
*Asparagus acutifolius* L.	+	1	+	1	.	1	+	1	1	1	1	1	+	+	+	1	.	.	1	.	1	+	1	+	1	.	+	+	.	1	+	+	.	+	1	1	1	1	.	.	.	**31**
*Arisarum vulgare* O.Targ.Tozz. subsp. *vulgare*	1	.	.	1	.	.	.	2	2	1	.	2	.	.	.	.	.	1	.	.	+	2	.	.	1	1	1	1	.	.	.	.	+	1	+	.	.	+	+	+	.	**19**
*Teucrium fruticans* L. subsp. *fruticans*	.	.	.	.	.	.	.	.	.	.	.	.	.	.	.	.	.	.	.	.	.	2	3	2	1	1	.	.	.	.	.	.	1	.	1	.	.	.	.	.	.	**7**
*Cytisus infestus* (C. Presl) Guss. subsp. *infestus*	.	.	.	.	+	.	.	.	2	.	.	.	.	.	+	+	.	.	.	+	.	.	.	.	.	.	.	.	.	.	.	.	.	.	.	.	.	.	.	.	.	**5**
*Phillyrea latifolia* L.	.	.	.	1	+	.	.	1	.	.	.	.	.	.	.	.	.	.	.	.	.	.	.	.	.	.	.	.	.	.	.	.	.	.	.	.	.	.	.	.	.	**3**
**Char. Cisto-Micromerietalia julianae**																																									
*Micromeria graeca* (L.) Benth. ex Rchb. subsp. *graeca*	.	.	.	.	.	.	.	.	.	.	.	.	.	.	.	.	.	.	.	.	.	.	.	.	.	.	.	.	.	.	.	.	.	.	1	+	1	2	.	.	.	**4**
*Phagnalon saxatile* (L.) Cass.	.	.	.	.	.	.	.	.	+	.	.	.	.	.	.	.	.	.	.	.	.	.	+	+	+	.	+	.	.	.	.	.	.	.	.	.	.	+	.	+	.	**7**
*Thymelaea hirsuta* (L.) Endl.	.	.	.	.	.	.	.	.	.	.	.	.	.	.	.	.	.	.	.	.	.	.	.	.	.	.	.	+	.	2	.	+	.	.	.	.	.	.	.	.	.	**3**
**Other species**																																									
*Dactylis glomerata* L. subsp. *hispanica* (Roth) Nyman	.	+	.	1	1	1	1	+	+	1	+	.	+	+	.	+	+	1	1	1	+	+	1	+	+	.	+	+	.	.	.	.	.	.	+	.	.	+	.	.	+	**26**
*Stachys major* (L.) Bartolucci & Peruzzi	3	2	.	2	2	1	2	1	1	1	+	.	.	.	.	+	.	2	2	1	1	+	.	1	.	.	.	.	.	.	+	+	+	+	+	+	+	.	1	1	.	**26**
*Charybdis pancration* (Steinh.) Speta	.	.	.	.	+	.	.	.	.	.	.	.	+	+	+	.	.	.	.	.	1	1	1	+	1	1	+	+	.	+	2	1	1	.	+	+	.	.	.	.	1	**19**
*Asphodelus ramosus* L. subsp. *ramosus*	.	.	.	.	+	.	.	+	+	.	.	.	.	.	.	.	.	.	.	+	+	+	1	.	.	1	+	+	.	.	+	+	1	1	1	3	.	.	1	+	.	**18**
*Avena barbata* Pott ex Link	+	+	+	+	.	+	+	.	.	+	.	.	.	.	.	.	.	+	.	+	+	.	+	.	.	.	.	.	.	.	.	.	+	.	+	.	.	+	+	1	+	**17**
*Lotus creticus* L.	.	.	.	.	.	.	.	.	.	.	.	.	.	.	.	.	.	.	.	.	.	.	.	.	.	+	.	.	+	1	.	.	+	+	+	+	1	+	1	2	+	**12**
*Thapsia garganica* L. subsp. *garganica*	+	.	+	1	1	.	.	.	.	+	.	.	+	.	+	+	.	.	+	.	.	+	+	.	.	.	.	.	.	.	.	.	.	.	.	+	.	.	.	.	.	**12**
*Scorpiurus subvillosus* L.	.	.	.	+	.	.	.	+	.	+	.	.	.	.	.	.	.	.	.	+	+	+	.	.	.	.	.	.	.	.	.	.	.	.	+	.	+	+	+	.	2	**11**
*Vicia sativa* L.	.	.	.	+	.	+	+	+	.	+	.	.	.	.	.	.	.	+	.	.	1	+	.	.	.	.	.	.	.	.	.	.	.	+	+	.	.	.	+	.	.	**11**
*Lotus edulis* L.	.	.	.	.	.	.	.	.	.	+	.	.	.	.	.	.	.	.	.	+	+	.	+	.	.	.	.	.	.	.	.	.	+	+	+	.	.	.	+	+	1	**10**
*Lysimachia arvensis* (L.) U. Manns & Anderb. subsp. *arvensis*	.	.	.	.	.	.	.	.	.	+	.	.	.	.	.	.	.	.	.	.	+	+	.	.	.	.	.	.	.	.	.	+	.	.	+	.	+	+	+	+	+	**10**
*Hyparrhenia hirta* (L.) Stapf subsp. *hirta*	.	.	.	.	.	+	.	.	.	1	.	.	.	.	+	.	.	.	.	.	.	+	.	+	+	.	.	.	.	.	.	.	.	2	1	.	.	.	1	.	.	**9**
*Sonchus bulbosus* (L.) N.Kilian & Greuter subsp. *bulbosus*	.	.	.	.	.	.	.	+	1	2	.	.	.	.	.	.	.	+	.	+	.	+	+	.	.	.	.	.	.	.	.	.	.	.	.	.	.	+	+	.	.	**9**
*Carex divisa* Huds.	.	.	.	.	1	.	.	.	.	.	2	3	+	.	+	.	.	.	.	.	.	.	.	.	+	.	+	+	.	.	.	.	.	.	.	.	.	.	.	.	.	**8**
*Plantago serraria* L.	.	+	.	.	.	.	.	.	+	+	.	.	.	.	.	.	.	.	.	.	+	.	+	.	.	.	.	.	.	.	.	.	+	.	+	1	.	.	.	.	.	**8**
*Urospermum dalechampii* (L.) F.W.Schmidt	.	.	.	.	.	.	+	.	.	.	.	.	.	.	.	.	.	.	.	.	.	.	.	.	.	.	.	.	.	+	.	+	+	+	+	.	.	+	.	.	+	**8**
*Convolvulus althaeoides* L.	.	.	.	.	.	.	+	.	.	.	.	.	.	.	.	.	.	.	.	.	+	.	.	.	.	.	.	.	.	+	.	.	+	+	+	.	+	.	.	.	.	**7**
*Hypericum perfoliatum* L.	.	.	.	.	.	+	+	.	.	.	.	.	.	.	.	.	.	.	.	+	+	+	+	.	.	.	.	.	.	.	.	.	.	.	+	.	.	.	.	.	.	**7**
*Lagurus ovatus* L. subsp. *ovatus*	.	.	.	.	.	.	.	.	.	.	.	.	.	.	.	.	.	.	.	.	.	.	.	.	.	.	.	.	.	+	+	+	+	.	+	.	+	+	.	.	.	**7**
*Lobularia maritima* (L.) Desv.	.	.	.	.	.	.	+	.	.	.	.	.	.	.	.	.	+	+	.	.	.	.	.	.	.	.	.	.	.	.	.	+	.	.	+	.	.	+	+	.	.	**7**
*Andropogon distachyos* L.	.	.	.	.	+	2	.	.	.	.	.	.	.	.	.	.	.	.	2	.	.	1	3	1	.	.	.	.	.	.	.	.	.	.	.	.	.	.	.	.	.	**6**
*Anisantha fasciculata* (C. Presl) Nevski subsp. *fasciculata*	.	.	.	.	.	.	.	.	.	+	.	.	.	.	.	.	.	.	.	.	.	.	.	.	.	.	.	.	.	.	.	.	+	.	+	.	+	+	+	.	.	**6**
*Brachypodium distachyon* (L.) P.Beauv.	.	+	.	.	.	.	+	.	.	.	.	.	.	.	.	.	.	.	.	.	.	.	.	.	.	.	.	.	.	.	.	.	.	.	+	+	.	.	2	+	.	**6**
*Briza maxima* L.	.	.	.	.	.	+	.	.	.	.	.	.	.	.	.	.	.	.	.	+	.	.	.	.	.	.	.	.	.	.	+	+	.	.	+	.	.	.	+	.	.	**6**
*Centaurium tenuiflorum* (Hoffmanns. and Link) Fritsch subsp. *tenuiflorum*	.	+	.	.	.	+	.	.	.	.	.	.	.	.	.	.	.	.	.	+	.	.	.	.	.	.	.	.	.	+	.	.	.	.	.	.	.	+	+	.	.	**6**
*Galactites tomentosus* Moench	.	.	1	.	.	.	.	.	.	.	.	.	.	.	.	.	.	.	.	.	.	.	r	.	.	.	.	.	.	.	.	.	.	.	.	+	.	.	+	+	+	**6**
*Lathyrus clymenum* L.	.	.	.	+	.	.	+	.	.	+	.	.	.	.	.	.	.	.	.	.	.	.	.	.	.	.	.	.	.	.	.	.	.	.	.	.	.	.	1	3	1	**6**
*Rumex bucephalophorus* L. subsp. *bucephalophorus*	.	.	.	.	.	.	.	.	.	.	.	.	.	.	.	.	.	.	.	.	.	.	.	.	.	.	.	.	.	.	+	+	.	.	+	.	+	+	+	.	.	**6**
*Lotus tetragonolobus* L.	.	.	.	.	.	.	.	.	.	+	.	.	.	.	.	.	.	.	.	.	.	.	.	.	.	.	.	.	.	.	.	.	+	+	.	+	.	+	.	.	.	**5**
*Mandragora autumnalis* Bertol.	.	.	.	.	.	.	.	.	+	.	.	+	.	.	.	.	.	.	.	.	.	+	.	.	.	+	.	.	.	.	.	.	.	+	.	.	.	.	.	.	.	**5**
*Ononis natrix* L. subsp. *ramosissima* (Desf.) Batt.	.	.	.	.	.	.	.	.	.	.	.	.	.	.	.	.	.	.	.	.	.	.	.	.	.	.	.	.	1	1	+	+	.	.	.	.	.	r	.	.	.	**5**
*Sonchus oleraceus* L.	.	.	.	.	.	.	.	.	.	+	.	.	.	.	.	.	.	+	.	.	.	.	.	.	.	.	.	.	.	.	.	.	.	.	.	.	.	.	+	+	+	**5**
*Valantia muralis* L.	.	.	+	.	.	.	.	.	.	.	.	.	.	.	.	.	.	.	.	.	.	.	.	.	.	.	.	.	.	.	+	+	.	.	.	.	.	+	+	.	.	**5**
*Vicia benghalensis* L.	1	.	.	1	.	.	.	.	.	.	.	.	.	.	.	.	.	.	.	.	.	.	.	.	.	.	.	.	.	.	.	.	1	1	3	.	.	.	.	.	.	**5**
*Bellardia trixago* (L.) All.	.	.	.	.	.	.	.	.	.	.	.	.	.	.	.	.	.	.	.	.	.	.	.	.	.	.	.	.	.	+	.	+	.	.	+	.	.	+	.	.	.	**4**
*Blackstonia perfoliata* (L.) Huds.	.	.	.	.	.	+	.	.	.	.	.	.	.	.	.	.	.	.	.	.	+	.	.	.	.	.	.	.	.	+	.	.	.	.	.	.	.	+	.	.	.	**4**
*Cachrys libanotis* L.	.	.	.	.	+	.	.	.	.	.	.	.	.	.	.	+	.	.	.	.	.	.	.	.	.	.	.	.	.	.	1	+	.	.	.	.	.	.	.	.	.	**4**
*Cachrys sicula* L.	.	.	.	.	.	.	.	.	.	.	.	.	+	1	.	.	.	.	.	.	.	.	.	.	.	.	.	.	.	.	+	+	.	.	.	.	.	.	.	.	.	**4**
*Carlina corymbosa* L.	.	.	.	.	.	.	.	.	.	.	.	.	.	.	.	.	.	.	.	.	.	.	.	.	.	.	.	.	.	+	.	+	.	+	+	.	.	.	.	.	.	**4**
*Catapodium rigidum* (L.) C.E.Hubb. subsp. *rigidum*	.	.	.	.	.	.	.	.	.	+	.	.	.	.	.	.	.	.	.	.	.	.	.	.	.	.	.	.	.	.	.	.	+	.	.	.	.	+	+	.	.	**4**
*Linum strictum* L.	.	.	.	.	.	.	.	.	.	.	.	.	.	.	.	.	.	.	.	.	.	.	.	.	.	.	.	.	.	+	.	.	.	.	+	.	.	+	.	.	1	**4**
*Lotus ornithopodioides* L.	.	.	.	.	.	.	.	.	.	+	.	.	.	.	.	.	.	.	.	.	.	+	.	.	.	.	.	.	.	.	.	.	.	.	+	.	.	.	.	.	+	**4**
*Reichardia picroides* (L.) Roth	.	.	.	.	.	.	+	+	.	+	.	.	.	.	.	.	.	.	.	.	.	.	.	.	.	.	.	.	.	.	.	.	.	.	.	.	.	.	.	.	+	**4**
*Stachys romana* (L.) E.H.L.Krause	.	.	+	.	.	.	.	.	.	.	.	.	.	.	.	.	.	.	.	.	.	.	.	.	.	.	.	.	.	.	.	.	.	.	.	.	.	.	+	+	+	**4**
*Silene colorata* Poir,	.	.	.	.	.	.	.	.	.	.	.	.	.	.	.	.	.	.	.	.	.	.	.	.	.	.	.	.	.	+	.	+	.	.	.	.	+	+	.	.	.	**4**
*Stipellula capensis* (Thunb.) Röser & H.R.Hamasha	.	.	.	.	.	.	.	.	.	.	.	.	.	.	.	.	.	.	.	.	.	.	.	.	.	.	.	.	.	.	.	.	.	.	.	.	.	+	+	+	+	**4**
*Ajuga iva* (L.) Schreb. subsp. *iva*	.	.	.	.	.	+	.	.	.	.	.	.	.	.	.	.	.	.	.	.	.	.	.	.	.	.	.	.	.	.	.	.	.	.	.	+	.	.	+	.	.	**3**
*Festuca myuros* L.	.	.	1	.	.	.	.	.	.	.	.	.	.	.	.	.	.	.	.	.	.	.	.	.	.	.	.	.	.	.	+	+	.	.	.	.	.	.	.	.	.	**3**
*Lotus cytisoides* L.	.	.	.	.	+	.	.	.	.	.	.	.	.	.	.	.	.	.	.	.	.	.	.	.	.	.	.	.	.	.	+	1	.	.	.	.	.	.	.	.	.	**3**
*Medicago littoralis* Rohde ex Loisel.	.	.	+	.	.	.	.	.	.	.	.	.	.	.	.	.	.	.	.	.	.	.	.	.	.	.	.	.	.	.	.	.	.	.	+	.	.	+	.	.	.	**3**
*Medicago minima* (L.) L.	.	.	.	.	.	.	.	+	.	.	.	.	.	.	.	.	.	.	.	.	.	.	.	.	.	.	.	.	.	.	.	.	+	.	.	.	.	+	.	.	.	**3**
*Medicago polymorpha* L.	.	.	.	.	.	.	.	.	.	+	.	.	.	.	.	.	.	.	.	.	.	.	.	.	.	.	.	.	.	.	.	.	.	.	.	.	.	.	+	.	+	**3**
*Mercurialis annua* L.	.	.	.	.	.	.	.	.	.	.	.	.	.	.	.	.	.	.	.	.	.	.	.	.	.	.	.	.	.	.	.	.	.	.	.	.	.	+	r	+	.	**3**
*Tripodion tetraphyllum* (L.) Fourr.	.	.	.	.	.	.	.	.	.	.	.	.	.	.	.	.	.	.	.	.	+	.	.	.	.	.	.	.	.	.	.	.	+	.	.	.	.	.	.	.	1	**3**
*Polypogon subspathaceus* Req.	.	.	.	.	.	.	.	.	.	.	.	.	+	+	+	.	.	.	.	.	.	.	.	.	.	.	.	.	.	.	.	.	.	.	.	.	.	.	.	.	.	**3**
*Rubia peregrina* L.	1	.	.	1	.	.	.	.	.	.	.	.	.	.	.	.	.	.	.	.	.	1	.	.	.	.	.	.	.	.	.	.	.	.	.	.	.	.	.	.	.	**3**
*Trifolium campestre* Schreb.	.	.	.	.	.	.	.	.	.	+	.	.	.	.	.	.	.	.	.	.	.	.	.	.	.	.	.	.	.	.	.	.	.	.	.	.	.	+	.	.	+	**3**
*Vicia bithynica* (L.) L.	.	.	.	.	.	.	+	.	.	.	.	.	.	.	.	.	.	.	.	.	2	.	.	.	.	.	.	.	.	.	.	.	.	.	.	.	.	.	.	.	1	**3**
*Anisantha madritensis* (L.) Nevski subsp. *madritensis*	.	.	.	.	.	.	.	.	.	.	.	.	.	.	.	.	.	.	.	.	.	.	.	.	.	.	.	.	.	.	.	.	.	.	.	.	.	.	+	+	.	**2**
*Avellinia festucoides* (Link) Valdés & H. Scholz	.	.	.	.	.	.	.	.	.	.	.	.	.	.	.	.	.	.	.	.	.	.	.	.	.	.	.	.	.	.	.	.	.	.	.	.	1	+	.	.	.	**2**
*Bromus hordeaceus* L. subsp. *hordeaceus*	.	.	2	.	.	.	.	.	.	.	.	.	.	.	.	.	.	.	.	.	.	.	.	.	.	.	.	.	1	.	.	.	.	.	.	.	.	.	.	.	.	**2**
*Centaurium pulchellum* (Sw.) Druce subsp. *pulchellum*	.	.	.	.	.	.	.	.	.	.	.	.	.	.	.	.	.	.	.	.	.	.	+	.	.	.	.	.	.	.	.	.	+	.	.	.	.	.	.	.	.	**2**
*Erodium malacoides* (L.) L’Hér. subsp. *malacoides*	.	.	.	.	.	.	.	.	.	.	.	.	.	.	.	.	.	.	.	.	.	+	.	.	.	.	.	.	.	.	.	.	.	.	.	.	.	.	+	.	.	**2**
*Euphorbia exigua* L. subsp. *exigua*	.	.	.	.	.	.	+	.	.	+	.	.	.	.	.	.	.	.	.	.	.	.	.	.	.	.	.	.	.	.	.	.	.	.	.	.	.	.	.	.	.	**2**
*Euphorbia peplus* L.	.	.	.	+	.	.	.	.	+	.	.	.	.	.	.	.	.	.	.	.	.	.	.	.	.	.	.	.	.	.	.	.	.	.	.	.	.	.	.	.	.	**2**
*Hypericum perforatum* L. subsp. *perforatum*	.	.	.	.	+	.	.	.	.	.	.	.	.	.	.	.	.	.	.	.	.	.	.	+	.	.	.	.	.	.	.	.	.	.	.	.	.	.	.	.	.	**2**
*Linum trigynum* L.	.	.	.	.	.	.	.	.	.	.	.	.	.	.	.	.	.	.	.	.	.	.	+	.	.	.	.	.	.	+	.	.	.	.	.	.	.	.	.	.	.	**2**
*Medicago orbicularis* (L.) Bartal.	.	.	.	.	.	.	.	.	.	.	.	.	.	.	.	.	.	.	.	.	.	.	.	.	.	.	.	.	.	.	.	.	.	.	.	.	.	.	+	.	+	**2**
*Medicago truncatula* Gaertn.	.	.	.	.	.	.	.	.	.	.	.	.	.	.	.	.	.	+	.	.	.	.	.	.	.	.	.	.	.	.	.	.	.	.	.	.	.	.	.	.	+	**2**
*Melica ciliata* L. subsp. *magnolii* (Godr. & Gren.) K.Richt.	.	.	.	.	.	.	1	.	.	.	.	.	.	.	.	.	.	.	.	.	.	.	+	.	.	.	.	.	.	.	.	.	.	.	.	.	.	.	.	.	.	**2**
*Melica minuta* L. subsp. *latifolia* (Coss.) W.Hempel	.	.	.	.	.	2	.	1	.	.	.	.	.	.	.	.	.	.	.	.	.	.	.	.	.	.	.	.	.	.	.	.	.	.	.	.	.	.	.	.	.	**2**
*Oloptum miliaceum* (L.) Röser and H.R.Hamasha	.	.	.	.	.	.	2	.	.	.	.	.	.	.	.	.	.	.	.	.	.	.	.	.	.	.	.	.	.	.	.	.	.	.	1	.	.	.	.	.	.	**2**
*Rostraria cristata* (L.) Tzvelev	.	.	.	.	.	.	.	.	.	.	.	.	.	.	.	.	.	.	.	.	.	.	.	.	.	.	.	.	.	.	.	.	.	.	.	.	+	+	.	.	.	**2**
*Rumex thyrsoides* Desf.	.	.	.	+	.	.	+	.	.	.	.	.	.	.	.	.	.	.	.	.	.	.	.	.	.	.	.	.	.	.	.	.	.	.	.	.	.	.	.	.	.	**2**
*Scolymus hispanicus* L. subsp. *hispanicus*	.	.	+	.	.	.	.	.	.	.	.	.	.	.	.	.	.	.	.	.	.	.	.	.	.	.	.	.	+	.	.	.	.	.	.	.	.	.	.	.	.	**2**
*Senecio coronopifolius* Desf.	.	.	.	.	.	.	.	.	.	.	.	.	.	.	.	.	.	.	.	.	.	.	.	.	.	.	.	.	.	.	.	.	.	.	.	.	+	+	.	.	.	**2**
*Sonchus tenerrimus* L.	.	.	.	+	.	.	.	.	.	.	.	.	.	.	.	.	.	.	.	.	.	.	.	.	.	.	.	.	.	.	.	.	.	.	.	.	.	.	.	+	.	**2**
*Tordylium apulum* L.	.	.	.	.	.	.	.	.	.	.	.	.	.	.	.	.	.	+	.	.	.	.	.	.	.	.	.	.	.	.	.	.	.	.	.	.	.	.	+	.	.	**2**
*Trifolium stellatum* L.	.	.	.	.	.	.	.	.	.	.	.	.	.	.	.	.	.	.	.	.	.	.	.	.	.	.	.	.	.	.	.	.	.	.	.	.	.	.	+	.	+	**2**
*Triglochin laxiflora* Guss.	.	.	.	+	.	.	.	.	.	.	.	+	.	.	.	.	.	.	.	.	.	.	.	.	.	.	.	.	.	.	.	.	.	.	.	.	.	.	.	.	.	**2**
*Urospermum picroides* (L.) Scop. ex F.W.Schmidt	.	.	.	+	.	.	.	.	.	+	.	.	.	.	.	.	.	.	.	.	.	.	.	.	.	.	.	.	.	.	.	.	.	.	.	.	.	.	.	.	.	**2**
*Vicia villosa* Roth	.	.	.	.	.	.	.	.	.	.	.	.	.	.	.	.	.	.	.	.	.	.	.	.	.	.	.	.	.	.	+	+	.	.	.	.	.	.	.	.	.	**2**
*Allium roseum* L. subsp. *roseum*	.	.	.	.	.	.	.	.	.	.	.	.	.	.	.	.	.	.	.	.	.	.	.	.	.	.	.	.	.	.	.	.	.	.	+	.	.	.	.	.	.	**1**
*Andryala integrifolia* L.	.	.	+	.	.	.	.	.	.	.	.	.	.	.	.	.	.	.	.	.	.	.	.	.	.	.	.	.	.	.	.	.	.	.	.	.	.	.	.	.	.	**1**
*Anisantha rigida* (Roth) Hyl.	.	.	.	.	.	.	.	.	.	.	.	.	.	.	.	.	.	.	.	.	.	.	.	.	.	.	.	.	.	.	.	.	.	.	.	.	.	+	.	.	.	**1**
*Barlia robertiana* (Loisel.) Greuter	.	.	.	.	.	.	.	.	.	.	.	.	.	.	.	.	.	.	.	.	.	.	.	.	.	.	.	.	.	.	.	.	r	.	.	.	.	.	.	.	.	**1**
*Brachypodium retusum* (Pers.) P.Beauv.	.	3	.	.	.	.	.	.	.	.	.	.	.	.	.	.	.	.	.	.	.	.	.	.	.	.	.	.	.	.	.	.	.	.	.	.	.	.	.	.	.	**1**
*Calendula arvensis* (Vaill.) L.	.	.	.	.	.	.	.	.	.	+	.	.	.	.	.	.	.	.	.	.	.	.	.	.	.	.	.	.	.	.	.	.	.	.	.	.	.	.	.	.	.	**1**
*Campanula erinus* L.	.	.	.	.	.	.	.	.	.	.	.	.	.	.	.	.	.	.	.	.	.	.	.	.	.	.	.	.	.	.	.	.	.	.	.	.	.	+	.	.	.	**1**
*Cerastium semidecandrum* L.	.	.	.	.	.	.	.	.	.	.	.	.	.	.	.	.	.	.	.	.	.	.	.	.	.	.	.	.	.	.	.	.	.	.	.	.	+	.	.	.	.	**1**
*Clinopodium nepeta* (L.) Kuntze subsp. *nepeta*	.	.	.	.	.	.	.	.	.	.	.	.	.	.	.	.	.	.	.	.	.	.	.	.	.	.	.	.	.	.	.	.	.	.	r	.	.	.	.	.	.	**1**
*Crassula tillaea* Lest.-Garl.	.	.	.	.	.	.	.	.	+	.	.	.	.	.	.	.	.	.	.	.	.	.	.	.	.	.	.	.	.	.	.	.	.	.	.	.	.	.	.	.	.	**1**
*Cynara cardunculus* L. subsp. *cardunculus*	.	1	.	.	.	.	.	.	.	.	.	.	.	.	.	.	.	.	.	.	.	.	.	.	.	.	.	.	.	.	.	.	.	.	.	.	.	.	.	.	.	**1**
*Daucus pumilus* (L.) Hoffmanns. & Link	.	.	.	.	.	.	.	.	.	.	.	.	.	.	.	.	.	.	.	.	.	.	.	.	.	.	.	.	.	.	.	.	.	.	.	.	.	+	.	.	.	**1**
*Dittrichia viscosa* (L.) Greuter subsp. *viscosa*	.	.	.	.	.	.	.	.	.	.	.	.	.	.	.	.	.	.	.	.	+	.	.	.	.	.	.	.	.	.	.	.	.	.	.	.	.	.	.	.	.	**1**
*Echinophora spinosa* L. *	.	.	.	.	.	.	.	.	.	.	.	.	.	.	.	.	.	.	.	.	.	.	.	.	.	.	.	.	+	.	.	.	.	.	.	.	.	.	.	.	.	**1**
*Erodium laciniatum* (Cav.) Willd. subsp. *laciniatum*	.	.	.	.	.	.	.	.	.	.	.	.	.	.	.	.	.	.	.	.	.	.	.	.	.	.	.	.	+	.	.	.	.	.	.	.	.	.	.	.	.	**1**
*Ervum tetraspermum* L.	.	.	.	.	.	.	.	2	.	.	.	.	.	.	.	.	.	.	.	.	.	.	.	.	.	.	.	.	.	.	.	.	.	.	.	.	.	.	.	.	.	**1**
*Euphorbia helioscopia* L. subsp. *helioscopia*	.	.	.	.	.	.	.	.	.	.	.	.	.	.	.	.	.	.	.	.	.	.	.	.	.	.	.	.	.	.	.	.	.	.	.	+	.	.	.	.	.	**1**
*Euphorbia terracina* L.	.	.	.	.	.	.	.	.	.	.	.	.	.	.	.	.	.	.	.	.	.	.	.	.	.	.	.	.	.	.	.	.	.	.	.	.	.	+	.	.	.	**1**
*Filago pygmaea* L.	.	.	.	.	.	.	.	.	.	.	.	.	.	.	.	.	.	.	.	.	.	.	.	.	.	.	.	.	.	.	.	.	.	.	.	.	+	.	.	.	.	**1**
*Galium murale* (L.) All.	.	.	.	.	.	.	.	.	+	.	.	.	.	.	.	.	.	.	.	.	.	.	.	.	.	.	.	.	.	.	.	.	.	.	.	.	.	.	.	.	.	**1**
*Gladiolus italicus* Mill.	.	.	.	+	.	.	.	.	.	.	.	.	.	.	.	.	.	.	.	.	.	.	.	.	.	.	.	.	.	.	.	.	.	.	.	.	.	.	.	.	.	**1**
*Hedypnois rhagadioloides* (L.) F.W.Schmidt	.	.	.	.	.	.	.	.	.	.	.	.	.	.	.	.	.	.	.	+	.	.	.	.	.	.	.	.	.	.	.	.	.	.	.	.	.	.	.	.	.	**1**
*Hyoseris scabra* L.	.	.	+	.	.	.	.	.	.	.	.	.	.	.	.	.	.	.	.	.	.	.	.	.	.	.	.	.	.	.	.	.	.	.	.	.	.	.	.	.	.	**1**
*Limonium virgatum* (Willd.) Fourr.	.	.	.	.	.	.	.	.	.	.	+	.	.	.	.	.	.	.	.	.	.	.	.	.	.	.	.	.	.	.	.	.	.	.	.	.	.	.	.	.	.	**1**
*Narcissus miniatus* Donn.-Morg., Koop. and Zonn.	.	.	.	.	.	.	.	.	.	.	.	+	.	.	.	.	.	.	.	.	.	.	.	.	.	.	.	.	.	.	.	.	.	.	.	.	.	.	.	.	.	**1**
*Onobrychis caput-galli* (L.) Lam.	.	.	.	.	.	.	.	.	.	.	.	.	.	.	.	.	.	.	.	.	.	.	.	.	.	.	.	.	.	.	.	.	.	.	.	.	.	.	.	.	+	**1**
*Oxalis pes-caprae* L.	.	.	.	.	.	.	.	.	.	.	.	.	.	.	.	.	.	.	.	.	.	.	.	.	.	+	.	.	.	.	.	.	.	.	.	.	.	.	.	.	.	**1**
*Pancratium maritimum* L.	.	.	.	.	.	.	.	.	.	.	.	.	.	.	.	.	+	.	.	.	.	.	.	.	.	.	.	.	.	.	.	.	.	.	.	.	.	.	.	.	.	**1**
*Phedimus stellatus* (L.) Raf.	.	.	.	.	.	.	.	.	.	.	.	.	.	.	.	.	.	.	.	.	.	.	.	.	.	.	.	.	.	.	.	.	.	.	.	.	.	.	+	.	.	**1**
*Poterium sanguisorba* L.	.	.	.	.	.	.	.	.	.	.	.	.	.	.	.	.	.	.	.	.	.	.	.	.	.	+	.	.	.	.	.	.	.	.	.	.	.	.	.	.	.	**1**
*Salvia clandestina* L.	.	.	.	.	.	.	.	.	.	.	.	.	.	.	.	.	.	.	.	.	.	.	.	.	.	.	.	.	.	.	.	.	.	.	.	.	.	+	.	.	.	**1**
*Sedum rubens* L.	.	.	.	.	.	.	.	.	+	.	.	.	.	.	.	.	.	.	.	.	.	.	.	.	.	.	.	.	.	.	.	.	.	.	.	.	.	.	.	.	.	**1**
*Silene nicaensis* All.	.	.	.	.	.	.	.	.	.	.	.	.	.	.	.	.	.	.	.	.	.	.	.	.	.	.	.	.	+	.	.	.	.	.	.	.	.	.	.	.	.	**1**
*Silene vulgaris* (Moench) Garcke subsp. *tenoreana* (Colla) Soldano & F.Conti	.	.	.	.	.	.	+	.	.	.	.	.	.	.	.	.	.	.	.	.	.	.	.	.	.	.	.	.	.	.	.	.	.	.	.	.	.	.	.	.	.	**1**
*Sixalix atropurpurea* (L.) Greuter & Burdet	.	.	.	.	.	.	.	.	.	.	.	.	.	.	.	.	.	.	.	.	.	.	.	.	.	.	.	.	.	.	.	.	.	.	.	.	.	r	.	.	.	**1**
*Sulla spinosissima* (L.) B.H.Choi & H.Ohashi	.	.	.	.	.	.	.	.	.	.	.	.	.	.	.	.	.	.	.	.	.	.	.	.	.	.	.	.	.	.	.	.	.	.	.	.	.	+	.	.	.	**1**
*Valerianella microcarpa* Loisel.	.	.	.	.	.	.	.	.	.	.	.	.	.	.	.	.	.	.	.	.	.	.	.	.	.	.	.	.	.	.	.	.	.	.	.	.	+	.	.	.	.	**1**

**Table A3:** Phytosociological relevés from the Island of Capo Passero (SE Sicily)—dry grasslands.

Cluster	3	3	3	3	3	3	3	3	3	3	3	3	3	3	3	3	3	3	3	3	3	3	3	3	3	3	3	3	3	4	4	4	4	4	4	4	4	4	4	4	4	4	4	4	4	4	4	4	4	4	4	**Occurrences**
Relevé number	28	30	29	31	32	34	33	35	66	42	64	43	44	81	45	46	47	48	54	36	41	173	39	40	59	60	121	62	63	37	38	49	50	51	113	52	55	56	107	195	196	197	53	111	112	58	57	72	82	73	86	
Shrub layer (cover %)	-	-	-	-	-	-	-	85	60	5	30	-	-	-	-	-	-	-	-	-	-	85	-	-	-	-	-	-	-	-	-	-	-	-	-	-	-	-	-				4	-	-	30	-	55	-	-	-	
Herbaceous layer (cover %)	70	85	90	40	80	60	40	40	55	85	55	95	80	5	95	90	60	80	75	85	90	-	65	65	55	55	-	30	5	70	80	65	85	70	85	30	80	65	80	80	80	60	45	15	40	5	15	15	45	90	55	
Shrub layer—mean height (cm)	-	-	-	-	-	-	-	50	70	50	60	-	-	-	-	-	-	-	-	-	-	-	-	-	-	-	-	-	-	-	-	-	-	-	-	-	-	-	-	-	-	-	20	-	-	20	-	60	-	-	-	
Herbaceous layer—mean height (cm)	25	15	30	15	15	15	30	20	20	40	30	50	20	2	10	10	25	10	15	25	30	-	25	12	10	5	-	3	3	20	30	10	20	20	8	10	15	15	8	-	-	-	7	4	10	10	10	20	15	45	20	
Plot size (square m)	100	50	100	50	40	100	20	100	60	70	80	100	25	2	10	5	100	25	50	100	150	50	100	20	1	2	-	1	0.5	100	50	100	100	50	100	5	50	50	100	100	100	50	4	30	20	20	4	50	25	50	25	
Exposure	-	-	-	-	-	SSW	S	-	-	-	-	-	-	S	WWN	WWN	NW	NW	-	W	-	-	-	-	-	-	-	-	-	S	W	W	-	-	-	-	-	-	-	-	-	-	S	SSE	N	-	S	-	S	-	-	
Slope (°)	-	-	-	-	-	15	-	-	-	-	-	-	-	-	-	-	1	1	-	-	-	-	-	-	-	-	-	-	-	-	-	10	-	-	-	-	-	-	-	-	-	-	20	-	5	-	-	-	-	-	-	
Stones and rocks (cover %)	-	-	-	-	10	80	65	-	-	-	-	-	-	-	-	-	-	-	-	-	60	-	-	-	-	-	-	-	-	45	40	45	-	-	35	60	40	-	85	-	-	-	-	-	-	-	-	-	-	-	-	
**Char. Saginetea maritimae**																																																				
*Catapodium balearicum* (Willk.) H.Scholz.	.	.	.	.	+	+	.	.	.	.	.	+	+	+	.	+	+	+	.	.	.	.	.	.	.	.	.	.	+	.	.	+	+	.	1	.	+	1	3	.	1	1	.	+	+	+	+	+	+	.	.	**23**
*Parapholis incurva* (L.) C.E.Hubb.	.	.	.	.	.	.	.	.	.	.	.	.	.	.	.	.	.	.	.	.	.	.	.	.	.	.	.	.	.	.	.	+	.	2	1	.	+	1	2	+	1	.	.	+	+	+	+	+	+	.	.	**14**
*Beta vulgaris* L. subsp. *maritima* (L.) Arcang.	.	.	.	.	.	.	.	.	.	.	.	.	.	.	.	.	.	+	.	.	.	.	.	.	.	.	.	.	.	1	2	+	+	2	4	.	.	+	3	.	.	.	.	+	.	.	.	.	.	.	.	**10**
*Centaurium tenuiflorum* (Hoffmanns. and Link) Fritsch subsp. *tenuiflorum*	+	.	.	.	.	.	.	.	.	.	.	.	+	.	+	.	.	.	+	.	.	.	.	.	1	+	.	.	.	.	.	.	+	+	.	+	+	.	.	.	.	.	.	.	.	.	.	.	.	.	.	**10**
*Mesembryanthemum nodiflorum* L.	.	.	.	.	.	+	.	.	.	.	.	.	.	.	.	.	.	.	.	.	.	.	.	.	.	.	.	.	.	1	2	2	1	+	1	.	.	.	.	.	.	.	.	.	+	.	.	.	.	.	.	**8**
*Plantago weldenii* Rchb.	.	.	.	.	.	.	.	.	.	.	.	.	.	.	.	.	.	.	.	.	.	.	.	.	+	+	.	.	.	.	.	.	+	.	.	.	.	.	1	.	.	.	+	.	+	.	.	.	.	.	.	**6**
*Polypogon subspathaceus* Req.	.	.	.	.	.	.	.	.	.	.	.	.	.	.	.	.	.	.	.	.	.	.	.	.	2	3	.	.	.	.	.	.	.	.	.	.	+	.	.	.	.	.	.	.	.	.	.	.	.	.	.	**3**
*Sagina maritima* Don	.	.	.	.	.	.	.	.	.	.	.	.	.	.	.	.	.	.	.	.	.	.	.	.	+	1	.	.	.	.	.	.	.	.	.	.	.	.	+	.	.	.	.	.	.	.	.	.	.	.	.	**3**
*Senecio pygmaeus* DC.	.	.	.	.	.	.	.	.	.	.	.	.	.	.	.	.	.	.	.	.	.	.	.	.	.	.	.	.	.	.	.	.	.	.	+	.	.	.	+	.	.	.	.	.	+	.	.	.	.	.	.	**3**
*Silene sedoides* Poir. subsp. *sedoides*	.	.	.	.	.	.	.	.	.	.	.	.	.	.	.	.	.	.	.	.	.	.	.	.	.	.	.	.	.	.	.	.	.	.	.	.	.	.	.	.	.	.	.	+	+	.	.	.	.	.	.	**2**
*Polycarpon tetraphyllum* (L.) L. subsp. *alsinifolium* (Biv.) Ball.	.	.	.	.	.	.	.	.	.	.	.	.	.	1	.	.	.	.	.	.	.	.	.	.	.	.	.	.	.	.	.	.	.	.	.	.	.	.	.	.	.	.	.	.	.	.	.	.	.	.	.	**1**
**Diff. Sub-halo-nitrophilous plants**																																																				
*Limonium sinuatum* (L.) Mill.	.	.	.	.	r	.	.	.	.	.	.	r	.	.	+	.	1	1	.	.	.	.	.	.	.	.	.	.	.	1	1	3	3	4	2	1	4	3	3	3	1	2	1	+	1	.	.	+	1	.	.	**23**
*Lotus creticus* L.	.	.	.	.	.	.	.	.	.	.	1	.	.	.	1	+	.	+	.	.	.	.	.	.	.	.	.	.	.	.	.	.	.	.	1	.	.	.	.	.	.	+	+	.	.	.	+	+	+	+	1	**12**
*Limonium virgatum* (Willd.) Fourr.	.	.	.	.	.	.	.	.	.	.	.	.	.	.	.	.	.	.	.	.	.	.	.	.	.	.	.	.	.	.	.	+	.	1	+	.	.	.	.	.	.	.	.	1	1	.	.	+	2	.	.	**7**
*Cichorium spinosum* L.	.	.	.	.	.	.	.	.	.	.	.	.	.	.	.	.	.	.	.	.	.	.	.	.	.	.	.	.	.	.	.	.	.	.	.	1	.	.	.	.	.	.	1	+	3	3	.	.	2	.	.	**6**
*Frankenia hirsuta* L.	.	.	.	.	.	.	.	.	.	.	.	.	.	.	.	.	.	.	.	.	.	.	.	.	.	.	.	.	.	.	.	.	.	+	2	.	.	.	1	.	.	.	.	+	+	.	.	.	.	.	.	**5**
*Aeluropus lagopoides* (L.) Trin. ex Thwaites	.	.	.	.	.	.	.	.	.	.	.	.	.	.	.	.	.	.	.	.	.	.	.	.	.	.	.	.	.	.	.	.	.	.	.	.	2	1	2	.	.	.	.	+	.	.	.	.	.	.	.	**4**
*Lotus cytisoides* L.	.	.	.	.	.	.	.	.	.	.	.	.	.	.	.	.	.	.	1	.	.	.	.	.	.	.	.	.	.	.	.	.	.	+	.	.	.	.	1	.	.	.	.	+	.	.	.	.	.	.	.	**4**
*Suaeda vera* J.F.Gmel.	.	.	.	.	.	.	.	.	.	.	.	.	.	.	.	.	.	.	.	.	.	.	.	.	.	.	.	.	.	.	.	.	.	.	.	.	.	.	+	.	.	.	.	.	.	.	.	.	.	.	.	**1**
*Crithmum maritimum* L.	.	.	.	.	.	.	.	.	.	.	.	.	.	.	.	.	.	.	.	.	.	.	.	.	.	.	.	.	.	.	.	.	.	.	.	.	.	.	.	.	.	.	.	+	.	.	.	.	.	.	.	**1**
**Char. Stipo-Trachynetea**																																																				
*Asteriscus aquaticus* (L.) Less.	+	+	3	2	4	1	.	.	.	+	.	1	.	.	4	+	3	3	1	.	1	.	+	3	1	1	.	.	.	2	2	3	3	2	+	2	3	.	.	1	2	+	+	+	1	.	.	.	.	.	.	**32**
*Stipellula capensis* (Thunb.) Röser & H.R.Hamasha	1	1	1	+	+	3	+	.	.	2	1	+	1	+	1	+	+	+	+	+	+	.	.	.	.	.	.	.	.	+	1	.	2	2	+	+	.	.	.	.	.	.	+	.	.	.	1	.	.	.	.	**27**
*Euphorbia peplis* L.	.	.	+	.	.	+	.	+	.	.	1	1	3	1	2	2	1	+	.	1	+	.	3	1	+	+	.	.	+	2	2	.	.	.	+	.	.	.	.	.	.	.	1	1	+	+	1	+	1	+	.	**29**
*Valantia muralis* L.	.	.	.	+	+	1	+	.	.	.	.	+	2	.	+	+	.	1	.	.	+	.	.	.	.	.	.	+	+	+	.	.	.	1	.	+	+	.	.	.	1	.	2	+	+	+	1	+	+	.	.	**24**
*Tordylium apulum* L.	.	.	+	+	+	2	+	.	.	.	.	.	.	.	+	.	+	+	.	.	+	.	.	.	.	.	.	.	.	.	.	.	.	+	.	+	.	.	.	.	.	.	.	.	+	.	.	.	.	.	.	**12**
*Sedum rubens* L.	+	+	+	.	1	+	.	.	.	.	.	.	.	.	.	.	.	.	.	.	.	.	.	1	+	.	.	1	1	.	.	.	+	.	.	+	2	+	.	.	.	.	+	.	+	.	.	.	.	.	.	**15**
*Catapodium rigidum* (L.) C.E.Hubb. subsp. *rigidum*	.	.	.	+	1	+	+	.	.	.	.	+	+	+	+	+	.	+	.	+	+	.	.	.	+	.	.	+	.	.	.	.	.	.	.	.	.	.	.	.	.	.	.	.	.	.	.	.	.	.	.	**14**
*Medicago littoralis* Rohde ex Loisel.	.	.	.	.	.	.	.	.	.	.	.	+	+	.	.	+	.	.	.	.	.	.	.	.	.	.	.	.	.	.	.	.	1	.	+	.	.	.	+	.	.	.	+	+	+	+	+	.	+	+	.	**13**
*Ajuga iva* (L.) Schreb. subsp. *iva*	.	+	.	+	+	.	+	.	.	+	+	+	+	.	+	.	.	.	.	1	+	.	.	+	.	.	.	.	.	.	.	.	.	.	.	.	.	.	.	.	.	.	.	.	.	.	.	.	.	.	.	**12**
*Anisantha fasciculata* (C. Presl) Nevski subsp. *fasciculata*	.	.	.	1	+	1	+	.	.	.	.	1	2	.	.	+	.	.	.	.	.	.	.	.	.	.	.	.	.	.	.	.	.	.	.	+	.	.	.	.	.	.	.	.	.	+	+	.	.	.	.	**10**
*Euphorbia exigua* L. subsp. *exigua*	+	+	+	+	+	.	+	.	.	.	.	.	.	.	.	.	.	+	.	.	+	.	.	.	+	.	.	.	.	.	.	.	.	.	.	.	.	.	.	.	.	.	.	.	.	+	.	.	.	.	.	**10**
*Scorpiurus subvillosus* L.	3	4	3	+	.	+	+	.	.	.	.	.	.	.	.	.	+	.	.	.	.	.	.	.	.	.	.	.	.	.	.	.	.	.	.	.	.	.	+	.	.	.	.	.	.	.	+	.	.	.	.	**9**
*Hypochoeris achyrophorus* L.	.	.	.	+	+	+	+	.	.	.	.	+	+	.	+	+	.	.	.	.	.	.	.	.	.	.	.	.	.	.	.	.	.	.	.	.	.	.	.	.	.	+	.	.	.	.	.	.	.	.	.	**9**
*Festuca fasciculata* Forssk.	.	.	.	.	.	.	.	.	.	4	1	.	1	+	.	.	.	.	.	.	.	.	.	.	.	.	.	.	.	.	.	.	.	.	.	+	.	.	.	.	.	.	.	+	.	.	+	+	.	+	.	**9**
*Lotus edulis* L.	+	+	+	.	.	.	.	.	.	+	.	.	.	.	.	.	+	2	.	.	.	.	.	.	.	.	.	.	.	.	.	.	1	.	.	.	.	.	.	.	.	.	.	.	.	+	.	.	.	.	.	**8**
*Hedypnois rhagadioloides* (L.) F.W.Schmidt	.	.	.	+	.	.	+	.	.	.	+	+	+	+	.	.	.	.	.	.	.	.	.	.	.	.	.	.	.	.	.	.	.	.	.	.	.	.	.	.	.	.	+	.	.	.	.	.	+	.	.	**8**
*Anisantha madritensis* (L.) Nevski subsp. *madritensis*	.	.	.	+	+	.	1	.	.	.	.	.	1	.	.	+	.	.	.	.	.	.	.	.	.	.	+	.	.	.	.	.	.	.	.	+	.	.	.	.	.	.	.	.	.	.	.	.	.	.	.	**7**
*Festuca myuros* L.	.	.	.	.	1	+	.	.	.	+	.	3	1	.	1	.	.	.	.	.	.	.	.	.	+	.	.	.	.	.	.	.	.	.	.	.	.	.	.	.	.	.	.	.	.	.	.	.	.	.	.	**7**
*Lotus ornithopodioides* L.	+	1	+	.	+	.	+	.	.	.	.	.	.	.	.	.	.	.	.	.	.	.	+	.	.	.	.	.	.	.	.	.	.	.	.	.	.	.	.	.	.	.	.	.	.	.	+	.	.	.	.	**7**
*Echium parviflorum* Moench	.	+	.	.	.	.	+	.	.	.	.	+	.	.	1	.	1	+	.	.	.	.	.	.	.	.	.	.	.	.	.	.	.	.	.	+	.	.	.	.	.	.	.	.	.	.	.	.	.	.	.	**7**
*Campanula erinus* L.	.	.	.	.	.	.	.	+	+	.	+	+	+	1	.	.	.	.	.	+	.	.	.	.	.	.	.	.	.	.	.	.	.	.	.	.	.	.	.	.	.	.	.	.	.	.	.	.	.	.	.	**7**
*Hyoseris scabra* L.	.	.	.	.	.	.	.	.	.	.	.	+	1	.	+	.	+	+	.	+	.	.	.	.	.	.	.	.	.	.	.	.	.	.	.	.	.	.	.	.	.	.	.	.	.	.	.	.	.	.	.	**6**
*Trifolium stellatum* L.	.	+	.	+	+	+	.	.	.	.	.	.	.	.	.	.	.	+	.	.	.	.	.	.	.	.	.	.	.	.	.	.	.	.	.	.	.	.	.	.	.	.	.	.	.	.	.	.	.	.	.	**5**
*Linum strictum* L.	+	+	1	.	.	+	.	.	.	+	.	.	.	.	.	.	.	.	.	.	.	.	.	.	.	.	.	.	.	.	.	.	.	.	.	.	.	.	.	.	.	.	.	.	.	.	.	.	.	.	.	**5**
*Trifolium scabrum* L.	.	.	.	.	.	+	.	.	.	.	.	.	.	.	.	.	.	.	.	.	.	.	.	.	.	.	.	.	.	.	.	.	+	.	.	.	.	.	.	.	.	.	.	.	+	+	.	.	.	.	.	**4**
*Filago pygmaea* L.	.	.	.	+	.	.	.	.	.	.	+	.	.	.	.	.	.	.	.	.	.	.	.	.	.	.	.	+	.	.	.	.	.	.	.	.	.	.	.	.	.	.	.	.	.	.	+	.	.	.	.	**4**
*Crassula tillaea* Lest.-Garl.	.	.	.	.	+	.	.	.	.	.	.	.	.	.	.	.	.	.	.	.	.	.	.	+	.	.	.	1	1	.	.	.	.	.	.	.	.	.	.	.	.	.	.	.	.	.	.	.	.	.	.	**4**
*Medicago truncatula* Gaertn.	1	2	1	.	.	.	.	.	.	.	.	.	.	.	.	.	.	.	.	.	.	.	.	.	.	.	.	.	.	.	.	.	.	.	.	.	.	.	.	.	.	.	.	.	.	.	.	.	.	.	.	**3**
*Plantago afra* L. subsp. *afra*	.	.	.	.	.	.	.	.	.	.	.	.	.	.	.	.	.	.	.	.	.	.	.	.	.	.	.	.	.	.	.	.	.	.	.	.	.	.	.	+	2	1	.	.	.	.	.	.	.	.	.	**3**
*Filago pyramidata* L.	.	.	+	+	.	.	.	.	.	.	.	.	.	.	.	.	.	.	.	.	.	.	.	.	.	.	.	.	.	.	.	.	.	.	.	.	.	.	.	.	.	.	.	.	.	.	.	.	.	.	.	**2**
*Lotus tetragonolobus* L.	.	+	.	.	.	+	.	.	.	.	.	.	.	.	.	.	.	.	.	.	.	.	.	.	.	.	.	.	.	.	.	.	.	.	.	.	.	.	.	.	.	.	.	.	.	.	.	.	.	.	.	**2**
*Brachypodium distachyon* (L.) P.Beauv.	.	.	.	.	.	+	.	.	.	.	.	.	.	.	.	.	.	.	.	.	.	.	.	.	.	+	.	.	.	.	.	.	.	.	.	.	.	.	.	.	.	.	.	.	.	.	.	.	.	.	.	**2**
*Anthemis secundiramea* Biv.	.	.	.	.	.	.	.	.	.	.	.	.	.	.	.	.	.	.	1	.	.	.	.	.	.	.	.	.	.	.	.	.	.	.	.	.	.	.	.	.	.	.	1	.	.	.	.	.	.	.	.	**2**
*Medicago minima* (L.) L.	.	.	.	.	+	.	.	.	.	.	.	.	+	.	.	.	.	.	.	.	.	.	.	.	.	.	.	.	.	.	.	.	.	.	.	.	.	.	.	.	.	.	.	.	.	.	.	.	.	.	.	**2**
*Sulla spinosissima* (L.) B.H.Choi & H.Ohashi	.	.	.	.	.	.	.	.	.	.	.	+	.	.	.	.	.	.	.	.	.	.	.	.	.	.	.	.	.	.	.	.	.	.	.	.	.	.	.	.	.	.	.	.	.	.	.	.	.	.	.	**1**
*Onobrychis caput-galli* (L.) Lam.	.	+	.	.	.	.	.	.	.	.	.	.	.	.	.	.	.	.	.	.	.	.	.	.	.	.	.	.	.	.	.	.	.	.	.	.	.	.	.	.	.	.	.	.	.	.	.	.	.	.	.	**1**
*Moraea sisyrinchium* (L.) Ker Gawl.	.	.	.	.	.	+	.	.	.	.	.	.	.	.	.	.	.	.	.	.	.	.	.	.	.	.	.	.	.	.	.	.	.	.	.	.	.	.	.	.	.	.	.	.	.	.	.	.	.	.	.	**1**
*Festuca myuros* L.	.	.	.	.	.	.	.	.	.	.	.	.	.	.	.	.	.	.	.	.	.	.	.	.	.	.	.	.	.	.	.	.	.	.	.	.	.	.	.	.	+	.	.	.	.	.	.	.	.	.	.	**1**
*Atractylis cancellata* L.	.	.	.	+	.	.	.	.	.	.	.	.	.	.	.	.	.	.	.	.	.	.	.	.	.	.	.	.	.	.	.	.	.	.	.	.	.	.	.	.	.	.	.	.	.	.	.	.	.	.	.	**1**
*Reichardia intermedia* (Sch. Bip.) Samp.	.	.	.	.	.	.	+	.	.	.	.	.	.	.	.	.	.	.	.	.	.	.	.	.	.	.	.	.	.	.	.	.	.	.	.	.	.	.	.	.	.	.	.	.	.	.	.	.	.	.	.	**1**
**Other species**																																																				
*Lysimachia arvensis* (L.) U.Manns & Anderb. subsp. *arvensis*	+	+	+	+	1	+	+	+	+	1	+	1	+	+	+	2	+	+	.	3	+	.	1	+	1	1	.	.	.	+	1	+	.	.	.	.	.	.	.	.	.	.	.	.	.	.	.	+	.	+	.	**29**
*Lobularia maritima* (L.) Desv.	+	.	.	.	+	2	.	.	+	.	+	+	+	+	+	+	+	.	.	+	+	.	1	+	.	.	.	.	.	2	+	+	+	.	.	+	.	.	.	.	.	.	.	.	+	.	.	+	.	+	.	**23**
*Thapsia garganica* L. subsp. *garganica*	1	+	.	1	1	1	+	.	.	1	1	.	.	.	+	.	+	.	.	2	1	3	1	1	.	.	.	.	.	1	1	1	+	+	2	.	r	.	.	.	.	.	.	.	.	.	.	.	.	.	.	**22**
*Rumex bucephalophorus* L. subsp. *bucephalophorus*	.	.	.	.	+	.	.	+	3	1	1	1	1	.	2	3	2	3	+	.	+	1	.	1	.	.	.	+	+	.	.	.	.	.	.	+	.	.	.	.	.	.	.	.	+	.	+	.	.	.	.	**20**
*Stachys romana* (L.) E.H.L.Krause	+	+	1	+	+	+	+	.	.	+	.	.	+	.	.	.	+	+	+	.	.	.	.	.	+	.	.	.	.	.	.	+	1	+	.	+	.	.	.	.	.	.	+	.	+	+	.	.	.	.	.	**20**
*Echium arenarium* Guss.	.	.	+	.	.	+	.	.	+	+	2	+	.	.	+	+	.	+	4	.	.	.	.	.	.	.	.	.	.	.	1	.	.	+	.	+	+	.	.	.	.	.	1	+	.	.	.	+	+	1	.	**19**
*Mandragora autumnalis* Bertol.	.	.	.	+	+	1	.	+	.	.	.	+	+	.	1	+	.	.	.	1	+	.	2	+	.	.	.	.	.	1	.	1	.	.	1	.	.	.	.	.	.	.	.	.	.	.	.	.	+	.	.	**16**
*Erodium malacoides* (L.) L’Hér. subsp. *malacoides*	.	+	+	+	+	+	+	.	.	.	.	+	.	.	.	+	+	+	.	.	.	.	.	.	.	.	.	.	.	.	+	+	+	+	.	.	.	.	.	.	.	.	.	.	.	+	.	.	.	.	.	**15**
*Reichardia picroides* (L.) Roth	.	+	.	.	.	+	+	.	.	.	.	.	+	.	.	+	+	+	.	.	+	.	.	.	.	.	.	.	.	.	.	+	.	+	+	+	.	.	.	.	.	.	.	.	+	+	1	.	.	.	.	**15**
*Lagurus ovatus* L. subsp. *ovatus*	.	.	.	.	.	+	.	.	.	+	+	+	+	+	+	+	.	.	.	+	.	.	.	.	.	.	.	.	.	.	.	.	.	.	.	.	.	.	.	.	+	+	.	+	.	.	.	+	.	+	.	**14**
*Scolymus hispanicus* L. subsp. *hispanicus*	.	.	.	.	.	+	.	.	+	.	1	.	.	.	.	.	.	.	1	.	.	1	.	+	.	.	.	.	.	2	.	1	.	+	.	.	.	.	.	.	.	.	.	+	1	.	.	.	.	1	1	**13**
*Calendula arvensis* (Vaill.) L.	.	+	.	+	.	1	+	+	.	.	.	+	.	.	.	+	+	.	.	+	+	.	.	+	.	.	.	.	.	+	.	.	.	.	.	.	.	.	.	.	.	.	.	.	.	.	.	.	.	.	.	**12**
*Erodium laciniatum* (Cav.) Willd. subsp. *laciniatum*	.	.	.	+	.	.	.	.	+	+	3	+	+	+	+	+	.	.	.	+	.	.	.	.	.	.	.	.	.	.	.	+	.	.	.	.	.	.	.	.	.	.	.	.	.	.	.	+	.	.	.	**12**
*Hordeum murinum* L. subsp. *leporinum* (Link) Arcang.	.	.	.	.	.	+	.	.	.	.	.	.	+	.	.	+	.	.	.	.	.	.	.	.	.	.	.	.	.	.	.	.	.	2	+	.	.	+	+	.	.	1	+	+	+	.	+	.	.	.	.	**12**
*Silene colorata* Poir,	.	.	.	.	.	.	.	+	2	+	+	+	+	+	.	.	.	.	.	+	.	.	.	.	.	.	.	.	.	.	.	.	.	.	.	.	.	.	.	.	.	.	.	.	.	.	.	+	+	1	+	**12**
*Sonchus bulbosus* (L.) N.Kilian & Greuter subsp. *bulbosus*	.	.	.	+	.	.	.	.	.	.	.	.	1	.	.	+	.	+	.	.	+	.	.	.	.	.	.	.	.	.	.	.	.	.	+	.	.	.	+	.	.	.	.	.	.	+	+	+	.	+	+	**12**
*Arisarum vulgare* O.Targ.Tozz. subsp. *vulgare*	.	.	.	+	.	+	.	2	.	.	.	.	+	.	.	.	.	.	.	1	1	2	2	2	.	.	.	.	.	.	.	+	.	.	3	.	.	.	.	.	.	.	.	.	.	.	.	.	.	.	.	**11**
*Galactites tomentosus* Moench	+	1	1	.	.	+	+	.	.	.	.	+	.	.	r	.	+	.	.	.	.	.	.	.	.	.	.	.	.	+	1	.	.	+	.	.	.	.	.	.	.	.	.	.	.	.	.	.	.	.	.	**11**
*Mercurialis annua* L.	.	.	.	.	.	.	.	+	1	r	.	.	+	.	r	.	+	.	.	+	+	.	+	.	.	.	.	.	.	1	1	.	.	.	.	.	.	.	.	.	.	.	.	.	.	.	.	.	.	.	.	**11**
*Carduus argyroa* Biv.	.	.	.	.	.	.	.	.	.	+	.	+	+	.	.	.	1	+	.	+	.	.	+	.	.	.	.	.	.	1	1	.	.	.	.	.	.	.	.	.	.	.	.	.	.	.	.	.	.	.	.	**9**
*Charybdis pancration* (Steinh.) Speta	2	3	2	1	1	1	.	.	.	.	.	.	.	.	.	.	.	.	.	1	+	1	.	.	.	.	.	.	.	.	.	.	.	.	.	.	.	.	.	.	.	.	.	.	.	.	.	.	.	.	.	**9**
*Hirschfeldia incana* (L.) Lagr.-Foss. subsp. *incana*	.	.	.	.	.	1	.	.	+	+	.	.	.	.	+	+	+	.	.	.	.	.	.	.	.	.	.	.	.	1	1	.	.	+	.	.	.	.	.	.	.	.	.	.	.	.	.	.	.	.	.	**9**
*Polycarpon tetraphyllum* (L.) L. subsp. *diphyllum* (Cav.) O. Bolòs & Font Quer	.	.	.	.	+	.	.	.	.	.	.	.	.	.	.	.	+	+	.	+	.	.	.	.	.	+	.	+	+	.	.	.	+	+	.	.	.	.	.	.	.	.	.	.	.	.	.	.	.	.	.	**9**
*Asphodelus ramosus* L. subsp. *ramosus*	1	.	.	.	+	.	1	.	.	.	.	.	.	.	.	.	.	.	.	3	4	2	+	1	.	.	.	.	.	.	.	.	.	.	.	.	.	.	.	.	.	.	.	.	.	.	.	.	.	.	.	**8**
*Plantago serraria* L.	2	+	3	2	+	+	.	.	.	+	.	1	.	.	.	.	.	.	.	.	.	.	.	.	.	.	.	.	.	.	.	.	.	.	.	.	.	.	.	.	.	.	.	.	.	.	.	.	.	.	.	**8**
*Rostraria cristata* (L.) Tzvelev	.	+	+	+	.	.	.	.	.	.	.	.	+	.	+	.	+	.	.	+	.	.	.	.	+	.	.	.	.	.	.	.	.	.	.	.	.	.	.	.	.	.	.	.	.	.	.	.	.	.	.	**8**
*Trifolium campestre* Schreb.	+	+	+	+	+	+	+	.	.	.	.	.	.	.	.	.	.	.	.	.	.	.	.	.	+	.	.	.	.	.	.	.	.	.	.	.	.	.	.	.	.	.	.	.	.	.	.	.	.	.	.	**8**
*Ecbalium elaterium* (L.) A.Rich.	.	.	.	.	.	.	.	+	.	.	.	.	.	.	.	r	.	.	.	1	.	.	2	.	.	.	.	.	.	1	+	.	.	+	.	.	.	.	.	.	.	.	.	.	.	.	.	.	.	.	.	**7**
*Ononis natrix* L. subsp. *ramosissima* (Desf.) Batt.	.	.	.	.	.	.	.	+	1	.	1	.	.	.	.	.	.	.	.	1	.	+	.	.	.	.	.	.	.	.	.	.	.	.	.	.	.	.	.	.	.	.	.	.	.	.	.	1	.	1	.	**7**
*Poterium spinosum* L.	.	.	+	.	.	.	.	3	+	1	2	1	1	.	.	.	.	.	.	.	.	.	.	.	.	.	.	.	.	.	.	.	.	.	.	.	.	.	.	.	.	.	.	.	.	.	.	.	.	.	.	**7**
*Sonchus oleraceus* L.	.	+	.	.	.	+	+	.	.	.	.	.	.	.	.	.	.	.	.	.	.	.	.	.	.	.	.	.	.	+	.	.	.	+	.	.	.	.	+	.	.	.	.	.	.	.	+	.	.	.	.	**7**
*Centaurium pulchellum* (Sw.) Druce subsp. *pulchellum*	.	.	.	.	.	+	+	.	.	+	.	+	.	.	.	.	.	.	.	.	.	.	.	.	.	.	.	.	.	.	.	.	.	.	.	.	+	.	.	.	.	.	+	.	.	.	.	.	.	.	.	**6**
*Euphorbia terracina* L.	.	.	.	.	.	.	.	+	.	.	1	.	.	.	.	.	.	.	.	1	.	2	.	.	.	.	.	.	.	.	.	.	.	.	.	.	.	.	.	.	.	.	.	.	.	.	.	+	.	1	.	**6**
*Allium ampeloprasum* L.	+	.	.	3	.	.	.	.	.	.	.	.	1	.	.	.	.	.	.	.	.	.	.	.	.	.	.	.	.	.	.	.	.	+	.	.	.	.	.	.	.	.	.	.	.	+	.	.	.	.	.	**5**
*Euphorbia peplus* L.	.	.	.	+	+	.	+	.	.	.	.	.	.	.	.	.	.	.	.	.	+	.	.	.	.	.	+	.	.	.	.	.	.	.	.	.	.	.	.	.	.	.	.	.	.	.	.	.	.	.	.	**5**
*Sonchus tenerrimus* L.	.	.	+	.	.	.	.	.	.	.	.	.	.	.	.	.	+	+	.	.	.	.	.	.	.	.	.	.	.	.	.	.	.	.	.	.	.	.	.	.	.	.	.	.	.	.	+	+	.	.	.	**5**
*Asparagus acutifolius* L.	.	.	.	.	.	+	.	+	+	.	.	.	+	.	.	.	.	.	.	.	.	.	.	.	.	.	.	.	.	.	.	.	.	.	.	.	.	.	.	.	.	.	.	.	.	.	.	.	.	.	.	**4**
*Astragalus boeticus* L.	.	.	.	.	.	.	.	.	.	.	.	.	.	.	+	+	.	+	.	+	.	.	.	.	.	.	.	.	.	.	.	.	.	.	.	.	.	.	.	.	.	.	.	.	.	.	.	.	.	.	.	**4**
*Cakile maritima* Scop. subsp. *maritima*	.	.	.	.	.	.	.	.	1	.	.	.	.	.	.	.	.	.	.	.	.	.	.	.	.	.	.	.	.	.	.	.	.	.	.	.	.	.	.	.	.	.	.	.	.	.	.	+	.	+	+	**4**
*Centaurea sphaerocephala* L. subsp. *sphaerocephala*	.	.	.	.	.	.	.	.	.	.	.	.	.	.	.	.	.	.	.	.	.	+	.	.	.	.	.	.	.	.	.	.	.	.	.	.	.	.	.	.	.	.	.	.	.	.	.	2	.	3	2	**4**
*Dactylis glomerata* L. subsp. *hispanica* (Roth) Nyman	+	.	.	.	.	2	.	.	.	+	.	.	.	.	.	.	.	.	.	.	.	.	.	.	.	.	.	.	.	.	.	+	.	.	.	.	.	.	.	.	.	.	.	.	.	.	.	.	.	.	.	**4**
*Daucus pumilus* (L.) Hoffmanns. & Link	.	.	.	.	.	.	.	.	+	.	.	.	.	.	.	.	.	.	.	.	.	.	.	.	.	.	.	.	.	.	.	.	.	.	.	.	.	.	.	.	.	.	.	.	.	.	.	+	+	+	.	**4**
*Malva cretica* Cav. subsp. *cretica*	.	+	+	+	.	.	+	.	.	.	.	.	.	.	.	.	.	.	.	.	.	.	.	.	.	.	.	.	.	.	.	.	.	.	.	.	.	.	.	.	.	.	.	.	.	.	.	.	.	.	.	**4**
*Oxalis pes-caprae* L.	.	.	.	.	.	.	.	1	.	.	.	.	.	.	.	.	.	.	.	2	.	2	.	+	.	.	.	.	.	.	.	.	.	.	.	.	.	.	.	.	.	.	.	.	.	.	.	.	.	.	.	**4**
*Pancratium maritimum* L.	.	.	.	.	.	.	.	.	.	.	.	.	.	.	.	.	.	.	.	.	.	.	.	.	.	.	.	.	.	.	.	.	.	.	.	.	.	.	.	.	.	.	.	.	.	.	.	1	1	+	1	**4**
*Salvia clandestina* L.	.	.	.	.	.	.	.	.	.	+	.	+	+	.	+	.	.	.	.	.	.	.	.	.	.	.	.	.	.	.	.	.	.	.	.	.	.	.	.	.	.	.	.	.	.	.	.	.	.	.	.	**4**
*Senecio coronopifolius* Desf.	.	.	.	.	.	.	.	.	+	.	.	.	.	.	.	.	.	.	.	.	.	.	.	.	.	.	.	.	.	.	.	.	.	.	.	.	.	.	.	.	.	.	.	.	.	.	.	+	+	+	.	**4**
*Solanum nigrum* L.	.	.	.	.	.	.	.	r	.	.	.	.	.	.	.	.	.	.	.	+	.	.	.	.	.	.	1	.	.	.	+	.	.	.	.	.	.	.	.	.	.	.	.	.	.	.	.	.	.	.	.	**4**
*Sonchus asper* (L.) Hill. subsp. *asper*	.	.	+	.	.	.	.	.	.	.	.	.	.	.	.	.	+	+	.	.	.	.	.	.	.	.	.	.	.		.	.	.	.	.	.	.	.	.	.	.	.	.	.	.	.	.	.	.	.	.	**4**
*Andryala integrifolia* L.	.	.	.	.	.	.	.	.	.	+	+	.	+	.	.	.	.	.	.	.	.	.	.	.	.	.	.	.	.	.	.	.	.	.	.	.	.	.	.	.	.	.	.	.	.	.	.	.	.	.	.	**3**
*Anisantha rigida* (Roth) Hyl.	.	.	.	.	.	.	.	+	.	.	.	.	.	.	.	.	.	.	.	.	.	.	.	.	.	.	.	.	.	.	.	.	.	+	.	.	.	.	.	.	.	.	.	.	.	.	.	.	.	+	.	**3**
*Cuscuta planiflora* Ten.	.	.	.	+	.	.	.	.	.	.	.	.	.	.	.	.	.	.	.	.	.	.	.	.	.	.	.	.	.	.	.	.	.	.	.	.	.	.	.	.	.	.	.	.	.	.	1	.	.	+	.	**3**
*Cynodon dactylon* (L.) Pers.	.	.	.	.	.	.	.	.	.	.	.	.	.	.	.	.	.	.	1	.	.	.	.	.	.	.	.	.	.	.	.	.	.	+	.	.	.	.	.	.	.	.	+	.	.	.	.	.	.	.	.	**3**
*Dittrichia viscosa* (L.) Greuter subsp. *viscosa*	.	.	.	.	.	.	.	2	3	.	.	.	.	.	.	.	.	.	.	.	.	+	.	.	.	.	.	.	.	.	.	.	.	.	.	.	.	.	.	.	.	.	.	.	.	.	.	.	.	.	.	**3**
*Echium plantagineum* L.	.	.	.	.	.	.	.	.	.	.	.	.	.	.	.	+	r	.	.	.	.	.	.	.	.	.	.	.	.	.	+	.	.	.	.	.	.	.	.	.	.	.	.	.	.	.	.	.	.	.	.	**3**
*Euphorbia helioscopia* L. subsp. *helioscopia*	.	.	.	+	.	.	.	.	.	.	.	.	.	.	.	.	+	+	.	.	.	.	.	.	.	.	.	.	.	.	.	.	.	.	.	.	.	.	.	.	.	.	.	.	.	.	.	.	.	.	.	**3**
*Medicago polymorpha* L.	.	+	.	.	+	.	.	.	.	.	.	.	.	.	.	.	.	.	.	.	.	.	.	.	.	.	.	.	.	.	.	.	.	.	.	.	.	.	.	.	.	.	.	.	.	+	.	.	.	.	.	**3**
*Silene nicaensis* All.	.	.	.	.	.	.	.	.	.	.	.	.	.	.	.	.	.	.	.	.	.	.	.	.	.	.	.	.	.	.	.	.	.	.	.	.	.	.	.	.	.	+	.	.	.	.	+	+	.	.	.	**3**
*Silene vulgaris* (Moench) Garcke subsp. *tenoreana* (Colla) Soldano & F.Conti	.	.	.	.	.	.	.	.	.	.	.	.	.	.	.	.	.	.	.	.	.	.	.	.	.	.	.	.	.	.	.	1	.	.	.	.	.	.	.	.	.	.	.	.	+	1	.	.	.	.	.	**3**
*Stachys major* (L.) Bartolucci & Peruzzi	.	.	.	.	.	+	.	+	.	.	.	.	.	.	.	.	.	.	.	.	+	.	.	.	.	.	.	.	.	.	.	.	.	.	.	.	.	.	.	.	.	.	.	.	.	.	.	.	.	.	.	**3**
*Trifolium nigrescens* Viv. subsp. *nigrescens*	.	.	.	.	.	.	.	.	.	.	.	.	.	.	+	+	+	.	.	.	.	.	.	.	.	.	.	.	.	.	.	.	.	.	.	.	.	.	.	.	.	.	.	.	.	.	.	.	.	.	.	**3**
*Urospermum picroides* (L.) Scop. ex F.W.Schmidt	.	.	.	.	.	.	+	.	.	.	.	.	.	.	+	.	.	.	.	.	.	.	.	.	.	.	.	.	.	.	.	.	.	.	.	.	.	.	.	.	.	.	.	.	.	+	.	.	.	.	.	**3**
*Arenaria serpyllifolia* L. subsp. *serpyllifolia*	.	.	.	.	.	.	.	.	.	.	.	.	.	.	.	.	.	.	.	.	.	.	.	.	.	.	.	.	.	.	.	.	.	.	.	.	.	.	.	+	.	+	.	.	.	.	.	.	.	.	.	**2**
*Asphodelus fistulosus* L.	.	.	.	.	.	.	.	.	.	.	.	.	.	.	.	.	.	.	.	1	.	.	.	.	3	.	.	.	.	.	.	.	.	.	.	.	.	.	.	.	.	.	.	.	.	.	.	.	.	.	.	**2**
*Avena barbata* Pott ex Link	.	.	.	.	.	.	.	.	.	.	.	.	.	.	.	.	.	.	.	+	.	.	.	.	.	.	.	.	.	.	.	.	.	.	.	.	.	.	.	.	.	.	.	.	.	.	.	.	.	r	.	**2**
*Avena sterilis* L. subsp. *sterilis*	.	.	.	+	.	+	.	.	.	.	.	.	.	.	.	.	.	.	.	.	.	.	.	.	.	.	.	.	.	.	.	.	.	.	.	.	.	.	.	.	.	.	.	.	.	.	.	.	.	.	.	**2**
*Blackstonia perfoliata* (L.) Huds.	+	.	.	.	.	.	.	.	.	.	.	.	.	.	.	.	.	.	.	.	.	.	.	.	+	.	.	.	.	.	.	.	.	.	.	.	.	.	.	.	.	.	.	.	.	.	.	.	.	.	.	**2**
*Borago officinalis* L.	.	.	.	.	.	.	.	.	.	.	.	.	.	.	.	+	r	.	.	.	.	.	.	.	.	.	.	.	.	.	.	.	.	.	.	.	.	.	.	.	.	.	.	.	.	.	.	.	.	.	.	**2**
*Carlina lanata* L.	.	.	+	.	.	+	.	.	.	.	.	.	.	.	.	.	.	.	.	.	.	.	.	.	.	.	.	.	.	.	.	.	.	.	.	.	.	.	.	.	.	.	.	.	.	.	.	.	.	.	.	**2**
*Chenopodiastrum murale* (L.) S. Fuentes, Uotila & Borsch	.	.	.	.	.	.	.	.	.	.	.	.	.	.	.	.	.	.	.	.	.	.	.	.	.	.	.	.	.	.	1	r	.	.	.	.	.	.	.	.	.	.	.	.	.	.	.	.	.	.	.	**2**
*Convolvulus althaeoides* L.	.	.	.	.	.	.	.	.	.	+	.	.	.	.	.	.	.	.	.	+	.	.	.	.	.	.	.	.	.	.	.	.	.	.	.	.	.	.	.	.	.	.	.	.	.	.	.	.	.	.	.	**2**
*Cynoglossum creticum* Mill.	.	.	.	.	.	.	.	.	.	.	.	.	.	.	.	.	.	.	.	+	.	.	.	.	.	.	.	.	.	.	.	.	.	.	.	.	.	.	.	.	.	.	.	.	.	.	.	.	.	+	.	**2**
*Erodium cicutarium* (L.) L’Hér.	.	.	.	.	.	.	.	.	.	.	.	.	.	.	.	.	+	1	.	.	.	.	.	.	.	.	.	.	.	.	.	.	.	.	.	.	.	.	.	.	.	.	.	.	.	.	.	.	.	.	.	**2**
*Euphorbia paralias* L.	.	.	.	.	.	.	.	.	.	.	.	.	.	.	.	.	.	.	.	.	.	.	.	.	.	.	.	.	.	.	.	.	.	.	.	.	.	.	.	.	.	.	.	.	.	.	.	.	.	1	1	**2**
*Galium verrucosum* Huds. subsp. *verrucosum*	.	.	.	.	.	.	.	.	.	.	.	.	.	.	.	.	.	.	.	+	+	.	.	.	.	.	.	.	.	.	.	.	.	.	.	.	.	.	.	.	.	.	.	.	.	.	.	.	.	.	.	**2**
*Gastridium ventricosum* (Gouan) Schinz & Thell.	+	.	.	.	.	+	.	.	.	.	.	.	.	.	.	.	.	.	.	.	.	.	.	.	.	.	.	.	.	.	.	.	.	.	.	.	.	.	.	.	.	.	.	.	.	.	.	.	.	.	.	**2**
*Hyoscyamus albus* L.	.	.	.	.	.	.	.	.	.	.	.	.	.	.	.	.	.	.	.	.	.	.	.	.	.	.	.	.	.	+	+	.	.	.	.	.	.	.	.	.	.	.	.	.	.	.	.	.	.	.	.	**2**
*Lagurus ovatus* L. subsp. *nanus* (Guss.) Messeri	.	.	.	.	.	.	.	.	.	.	.	.	.	.	.	.	.	.	.	.	.	.	.	.	.	.	.	.	.	.	.	.	.	.	+	.	.	.	.	.	.	.	.		.	.	.	.	.	.	.	**2**
*Lythrum hyssopifolia* L.	.	.	.	.	.	.	.	.	.	.	.	.	.	.	.	.	.	.	.	.	.	.	.	.	+	+	.	.	.	.	.	.	.	.	.	.	.	.	.	.	.	.	.	.	.	.	.	.	.	.	.	**2**
*Matthiola tricuspidata* (L.) R.Br.	.	.	.	.	.	.	.	.	.	.	.	.	.	.	.	.	.	.	.	.	.	.	.	.	.	.	.	.	.	.	.	.	.	.	.	.	.	.	.	.	.	.	.	.	.	.	.	+	+	.	.	**2**
*Nigella damascena* L.	.	.	.	.	.	.	.	.	.	.	.	.	+	.	.	.	.	.	.	+	.	.	.	.	.	.	.	.	.	.	.	.	.	.	.	.	.	.	.	.	.	.	.	.	.	.	.	.	.	.	.	**2**
*Phedimus stellatus* (L.) Raf.	.	.	.	.	.	.	.	.	.	.	.	.	.	.	.	.	.	.	.	.	.	.	.	.	.	.	.	1	+	.	.	.	.	.	.	.	.	.	.	.	.	.	.	.	.	.	.	.	.	.	.	**2**
*Polypogon monspeliensis* (L.) Desf.	.	.	.	.	.	.	.	.	.	.	.	.	.	.	.	.	.	.	.	.	.	.	.	.	.	.	.	.	.	.	.	.	.	.	.	.	.	.	.	2	+	.	.	.	.	.	.	.	.	.	.	**2**
*Poterium sanguisorba* L.	.	.	.	+	.	.	.	.	.	.	.	.	.	.	.	.	.	.	.	.	+	.	.	.	.	.	.	.	.	.	.	.	.	.	.	.	.	.	.	.	.	.	.	.	.	.	.	.	.	.	.	**2**
*Sagina apetala* Ard. subsp. *apetala*	.	.	.	.	.	.	.	.	.	.	.	.	.	.	.	.	.	.	.	.	.	.	.	.	.	.	.	+	1	.	.	.	.	.	.	.	.	.	.	.	.	.	.	.	.	.	.	.	.	.	.	**2**
*Thymelaea hirsuta* (L.) Endl.	.	.	.	.	.	.	.	.	.	.	.	.	.	.	.	.	.	.	.	.	.	.	.	.	.	.	.	.	.	.	.	.	.	.	.	.	.	.	.	.	.	.	.	.	.	.	.	3	1	.	.	**2**
*Tripodion tetraphyllum* (L.) Fourr.	.	+	1	.	.	.	.	.	.	.	.	.	.	.	.	.	.	.	.	.	.	.	.	.	.	.	.	.	.	.	.	.	.	.	.	.	.	.	.	.	.	.	.	.	.	.	.	.	.	.	.	**2**
*Urospermum dalechampii* (L.) F.W.Schmidt	.	.	.	.	.	.	+	.	.	.	.	.	.	.	.	.	.	.	.	.	.	.	.	.	.	.	3	.	.	.	.	.	.	.	.	.	.	.	.	.	.	.	.	.	.	.	.	.	.	.	.	**2**
*Briza maxima* L.	.	.	.	.	.	.	+	.	.	.	.	.	.	.	.	.	.	.	.	.	.	.	.	.	.	.	.	.	.	.	.	.	.	.	.	.	.	.	.	.	.	.	.	.	.	.	.	.	.	.	.	**1**
*Bromus scoparius* L.	.	.	.	.	.	+	.	.	.	.	.	.	.	.	.	.	.	.	.	.	.	.	.	.	.	.	.	.	.	.	.	.	.	.	.	.	.	.	.	.	.	.	.	.	.	.	.	.	.	.	.	**1**
*Cachrys libanotis* L.	.	.	.	.	.	.	.	.	.	.	.	.	.	.	.	.	.	.	.	.	.	2	.	.	.	.	.	.	.	.	.	.	.	.	.	.	.	.	.	.	.	.	.	.	.	.	.	.	.	.	.	**1**
*Capparis orientalis* Veill.	.	.	.	.	.	.	.	.	.	.	.	.	.	.	.	.	.	.	.	.	.	.	.	.	.	.	.	.	.	1	.	.	.	.	.	.	.	.	.	.	.	.	.	.	.	.	.	.	.	.	.	**1**
*Carlina corymbosa* L.	.	.	.	.	.	+	.	.	.	.	.	.	.	.	.	.	.	.	.	.	.	.	.	.	.	.	.	.	.	.	.	.	.	.	.	.	.	.	.	.	.	.	.	.	.	.	.	.	.	.	.	**1**
*Cerastium semidecandrum* L.	.	.	.	.	.	.	.	.	+	.	.	.	.	.	.	.	.	.	.	.	.	.	.	.	.	.	.	.	.	.	.	.	.	.	.	.	.	.	.	.	.	.	.	.	.	.	.	.	.	.	.	**1**
*Cerinthe major* L. subsp. *major*	.	.	.	.	.	.	.	.	.	.	.	.	.	.	.	.	.	.	.	.	+	.	.	.	.	.	.	.	.	.	.	.	.	.	.	.	.	.	.	.	.	.	.	.	.	.	.	.	.	.	.	**1**
*Cynara cardunculus* L. subsp. *cardunculus*	.	.	.	.	.	.	.	.	.	.	.	.	.	.	.	.	.	.	.	.	.	.	.	.	.	.	.	.	.	+	.	.	.	.	.	.	.	.	.	.	.	.	.	.	.	.	.	.	.	.	.	**1**
*Daucus carota* L. subsp. *carota*	.	.	.	.	.	.	.	.	.	.	.	.	.	.	.	.	.	.	.	.	.	.	.	.	.	.	.	.	.	.	r	.	.	.	.	.	.	.	.	.	.	.	.	.	.	.	.	.	.	.	.	**1**
*Eryngium campestre* L.	.	.	.	.	.	.	.	.	.	.	.	.	.	.	.	.	.	.	.	.	1	.	.	.	.	.	.	.	.	.	.	.	.	.	.	.	.	.	.	.	.	.	.	.	.	.	.	.	.	.	.	**1**
*Galium murale* (L.) All.	.	.	.	.	.	.	.	.	.	.	.	.	.	.	.	.	.	.	.	.	.	.	.	.	.	.	.	+	.	.	.	.	.	.	.	.	.	.	.	.	.	.	.	.	.	.	.	.	.	.	.	**1**
*Geranium molle* L.	.	.	.	.	.	.	.	.	.	.	.	.	.	.	.	+	.	.	.	.	.	.	.	.	.	.	.	.	.	.	.	.	.	.	.	.	.	.	.	.	.	.	.	.	.	.	.	.	.	.	.	**1**
*Gladiolus italicus* Mill.	+	.	.	.	.	.	.	.	.	.	.	.	.	.	.	.	.	.	.	.	.	.	.	.	.	.	.	.	.	.	.	.	.	.	.	.	.	.	.	.	.	.	.	.	.	.	.	.	.	.	.	**1**
*Glaucium flavum* Crantz	.	.	.	.	.	.	.	.	.	.	.	.	.	.	.	.	.	.	.	.	.	+	.	.	.	.	.	.	.	.	.	.	.	.	.	.	.	.	.	.	.	.	.	.	.	.	.	.	.	.	.	**1**
*Hedypnois cretica* (L.) Willd.	.	.	.	.	.	.	.	.	.	.	.	.	.	.	.	.	.	.	.	.	.	.	.	.	.	.	.	.	.	.	.	.	.	.	.	.	.	.	.	.	.	.	.	.	.	.	+	.	.	.	.	**1**
*Hyparrhenia hirta* (L.) Stapf subsp. *hirta*	.	.	.	.	.	.	2	.	.	.	.	.	.	.	.	.	.	.	.	.	.	.	.	.	.	.	.	.	.	.	.	.	.	.	.	.	.	.	.	.	.	.	.	.	.	.	.	.	.	.	.	**1**
*Hypericum perfoliatum* L.	.	.	.	.	.	.	.	.	.	.	.	.	.	.	.	.	.	.	.	.	.	.	.	+	.	.	.	.	.	.	.	.	.	.	.	.	.	.	.	.	.	.	.	.	.	.	.	.	.	.	.	**1**
*Logfia gallica* (L.) Cosson & Germ.	.	.	.	.	.	.	.	.	.	.	.	.	.	1	.	.	.	.	.	.	.	.	.	.	.	.	.	.	.	.	.	.	.	.	.	.	.	.	.	.	.	.	.	.	.	.	.	.	.	.	.	**1**
*Medicago orbicularis* (L.) Bartal.	.	.	.	+	.	.	.	.	.	.	.	.	.	.	.	.	.	.	.	.	.	.	.	.	.	.	.	.	.	.	.	.	.	.	.	.	.	.	.	.	.	.	.	.	.	.	.	.	.	.	.	**1**
*Orchis italica* Poir.	.	.	.	.	.	+	.	.	.	.	.	.	.	.	.	.	.	.	.	.	.	.	.	.	.	.	.	.	.	.	.	.	.	.	.	.	.	.	.	.	.	.	.	.	.	.	.	.	.	.	.	**1**
*Ornithogalum gussonei* Ten.	.	.	.	+	.	.	.	.	.	.	.	.	.	.	.	.	.	.	.	.	.	.	.	.	.	.	.	.	.	.	.	.	.	.	.	.	.	.	.	.	.	.	.	.	.	.	.	.	.	.	.	**1**
*Orobanche minor* Sm.	.	.	.	.	.	.	.	.	.	.	.	.	.	.	+	.	.	.	.	.	.	.	.	.	.	.	.	.	.	.	.	.	.	.	.	.	.	.	.	.	.	.	.	.	.	.	.	.	.	.	.	**1**
*Papaver hybridum* L.	.	.	.	.	.	.	.	.	.	.	.	.	.	.	.	.	.	.	.	+	.	.	.	.	.	.	.	.	.	.	.	.	.	.	.	.	.	.	.	.	.	.	.	.	.	.	.	.	.	.	.	**1**
*Papaver somniferum* L.	.	.	.	.	.	.	.	.	.	.	.	.	.	.	.	.	.	.	.	.	.	.	+	.	.	.	.	.	.	.	.	.	.	.	.	.	.	.	.	.	.	.	.	.	.	.	.	.	.	.	.	**1**
*Phalaris paradoxa* L.	.	.	.	.	.	+	.	.	.	.	.	.	.	.	.	.	.	.	.	.	.	.	.	.	.	.	.	.	.	.	.	.	.	.	.	.	.	.	.	.	.	.	.	.	.	.	.	.	.	.	.	**1**
*Pistacia lentiscus* L.	.	.	.	.	.	.	.	.	.	.	.	.	.	.	.	.	.	.	.	.	1	.	.	.	.	.	.	.	.	.	.	.	.	.	.	.	.	.	.	.	.	.	.	.	.	.	.	.	.	.	.	**1**
*Rumex pulcher* L. subsp. *pulcher*	.	.	.	.	.	.	.	.	.	.	.	.	.	.	.	.	.	.	.	+	.	.	.	.	.	.	.	.	.	.	.	.	.	.	.	.	.	.	.	.	.	.	.	.	.	.	.	.	.	.	.	**1**
*Rumex spinosus* L.	.	.	.	.	.	.	.	.	.	.	.	.	.	.	.	.	.	.	.	1	.	.	.	.	.	.	.	.	.	.	.	.	.	.	.	.	.	.	.	.	.	.	.	.	.	.	.	.	.	.	.	**1**
*Rumex thyrsoides* Desf.	+	.	.	.	.	.	.	.	.	.	.	.	.	.	.	.	.	.	.	.	.	.	.	.	.	.	.	.	.	.	.	.	.	.	.	.	.	.	.	.	.	.	.	.	.	.	.	.	.	.	.	**1**
*Theligonum cynocrambe* L.	.	.	.	.	.	.	.	.	.	.	.	.	.	.	.	.	.	.	.	.	+	.	.	.	.	.	.	.	.	.	.	.	.	.	.	.	.	.	.	.	.	.	.	.	.	.	.	.	.	.	.	**1**
*Verbascum sinuatum* L.	.	.	.	.	.	.	.	.	.	.	.	.	.	.	.	.	.	.	.	.	.	.	.	1	.	.	.	.	.	.	.	.	.	.	.	.	.	.	.	.	.	.	.	.	.	.	.	.	.	.	.	**1**
*Vicia benghalensis* L.	.	.	.	.	.	.	+	.	.	.	.	.	.	.	.	.	.	.	.	.	.	.	.	.	.	.	.	.	.	.	.	.	.	.	.	.	.	.	.	.	.	.	.	.	.	.	.	.	.	.	.	**1**

**Table A4:** Phytosociological relevés from the Island of Capo Passero (SE Sicily)—psammophilous vegetation.

Cluster	5	5	5	5	5	5	5	5	5	5	5	5	5	5	5	5	5	5	5	5	5	5	5	5	6	6	6	6	6	6	7	7	7	7	7	7	7	
Relevé number	65	126	127	67	68	70	69	71	200	201	74	75	76	77	87	174	175	199	122	177	176	125	124	178	78	179	123	79	80	85	83	162	84	158	159	160	161	
Shrub layer (cover %)	-	75	75	15	40	40	70	30	80	60	-	-	30	-	45	70	65	30	90	90	70	90	85	90	15	85	90	10	10		-	70	-	50	65	75	70	
Herbaceous layer (cover %)	85	-	-	45	45	55	40	60	-	-	45	55	20	20		-	-	-	-	-	-	-	-	-	60	-	-	70	85	45	40	-	10	-	-	-	-	
Shrub layer—mean height (cm)	-	-	-	50	50	55	60	45	-	-	-	-	45	-	-	-	-	-	-	-	-	-	-	-	40	-	-	40	40	-	-	-	-	-	-	-	-	
Herbaceous layer—mean height (cm)	10	-	-	25	35	15	20	40	-	-	5	5	20	15	35	-	-	-	-	-	-	-	-	-	60	-	-	90	80	70	30	-	35	-	-	-	-	
Plot size (square m)	4	4	4	30	100	100	50	100	50	100	2	2	20	20	30	20	20	100	100	20	20	100	100	20	20	20	25	50	50	20	30	10	60	10	10	10	10	
Exposure	-	-	-	-	-	-	-	-	S	S	WWN	S	-	E	-	-	-	-	-	-	-	-	-	-	-	-	-	-	-	-	-	-	-	-	-	-	-	
Slope (°)	-	-	-	-	-	-	-	-	10	20	-	-	-	-	-	-	-	-	-	-	-	-	-	-	-	-	-	-	-	-	-	-	-	-	-	-	-	
**Char. Ass.**																																					
*Ononis natrix* L. subsp. *ramosissima* (Desf.) Batt.	+	+	+	2	4	4	4	3	2	3	+	+	3	+	2	3	3	.	4	4	4	4	3	3	2	2	3	1	1	.	.	.	.	.	.	.	.	**28**
*Calamagrostis arenaria* (L.) Roth subsp. *arundinacea* (Husn.) Banfi, Galasso & Bartolucci	.	.	.	.	.	.	.	.	.	.	.	.	.	.	.	.	.	1	.	.	.	1	.	.	5	4	3	4	5	3	.	.	+	.	.	.	.	**9**
*Cakile maritima* Scop. subsp. *maritima*	.	.	.	+	3	1	1	3	.	.	.	.	.	.	.	1	+	.	.	+	.	.	.	+	.	+	.	1	1	+	3	3	1	2	3	3	3	**20**
**Char. Ammophiletea**																																					
*Euphorbia terracina* L.	.	.	.	1	1	1	1	1	.	.	1	.	+	+	.	1	1	.	2	2	1	2	.	1	1	1	1	+	1	.	.	.	.	+	1	+	+	**24**
*Scolymus hispanicus* L. subsp. *hispanicus*	.	.	.	2	1	1	1	2	+	.	.	.	.	.	+	+	1	.	1	1	2	1	1	1	+	2	+	.	.	.	.	.	.	+	+	+	+	**22**
*Euphorbia paralias* L.	.	.	.	.	.	.	.	.	+	1	.	.	1	2	3	2	3	2	.	+	.	.	.	.	.	.	.	.	.	1	1	.	1	.	.	.	.	**12**
*Centaurea sphaerocephala* L. subsp. *sphaerocephala*	.	.	.	.	.	.	2	2	.	.	+	.	.	.	.	.	.	.	1	2	1	.	3	3	.	.	.	.	.	.	.	.	.	.	.	.	.	**8**
*Lotus creticus* L.	+	.	+	.	.	.	.	.	1	+	+	1	.	.	.	.	.	.	1	+	.	+	+	+	.	.	.	.	.	1	.	.	.	.	.	.	.	**12**
*Pancratium maritimum* L.	.	.	.	.	.	+	+	+	.	.	.	.	+	1	.	+	+	+	.	.	+	+	+	.	.	.	.	.	.	.	.	.	.	.	.	.	.	**11**
*Echinophora spinosa* L.	.	.	.	.	.	.	.	.	+	.	.	.	.	.	.	.	.	+	.	.	.	.	.	.	.	.	.	.	.	.	.	.	.	.	.	.	.	**2**
*Lotus cytisoides* L.	.	.	.	.	.	.	.	.	.	.	.	.	.	.	.	.	.	.	.	.	.	.	.	.	.	.	+	.	.	.	.	.	.	.	.	.	.	**1**
*Seseli tortuosum* L. subsp. *maritimum* C. Brullo, Brullo, Giusso & Sciandr.	.	.	.	.	.	.	+	+	.	.	.	.	.	.	.	.	.	.	.	.	.	.	.	.	.	.	.	.	.	.	.	.	.	.	.	.	.	**2**
*Sporobolus virginicus* (L.) Kunth	.	.	.	.	.	.	.	.	.	.	.	.	.	.	.	.	.	+	.	.	.	.	.	.	.	.	.	.	.	.	.	.	.	.	.	.	.	**1**
*Achillea maritima* (L.) Ehrend. & Y.P.Guo subsp. *maritima*	.	.	.	.	.	.	.	.	.	.	.	.	.	.	.	.	.	+	.	.	.	.	.	.	.	.	.	.	.	.	.	.	.	.	.	.	.	**1**
*Eryngium maritimum* L.	.	.	.	.	.	.	.	.	.	.	.	.	.	.	.	.	.	+	.	.	.	.	.	.	.	.	.	.	.	.	.	.	.	.	.	.	.	**1**
**Oltre species**																																					
*Echium arenarium* Guss.	1	1	1	+	+	1	1	2	+	.	2	1	1	1	+	.	+	.	.	.	.	.	+	+	.	.	.	.	.	.	.	.	.	.	.	.	.	**17**
*Lysimachia arvensis* (L.) U. Manns & Anderb. subsp. *arvensis*	1	.	+	.	1	+	+	+	.	.	+	+	+	+	.	.	.	.	+	.	.	+	+	.	+	.	+	+	.	.	.	.	.	.	.	.	.	**16**
*Silene colorata* Poir.	3	2	4	.	2	1	1	1	.	.	+	+	1	.	.	.	.	.	+	.	.	+	+	.	+	.	+	.	+	.	.	.	.	.	.	.	.	**16**
*Silene nicaensis* All.	.	3	2	1	1	1	1	1	+	1	+	+	+	+	+	.	.	.	.	.	.	.	+	+	.	.	.	.	.	.	.	.	.	.	.	.	.	**16**
*Erodium laciniatum* (Cav.) Willd. subsp. *laciniatum*	1	3	3	+	1	2	1	+	1	+	+	+	+	+	.	.	.	.	.	.	.	.	.	.	.	.	.	.	+	.	.	.	.	.	.	.	.	**15**
*Daucus pumilus* (L.) Hoffmanns. & Link	.	.	.	2	+	+	1	1	+	2	1	+	+	.	.	.	.	+	.	.	.	.	.	.	.	.	.	+	+	1	.	.	.	.	.	.	.	**14**
*Euphorbia peplis* L.	+	.	.	.	.	1	+	.	.	.	1	1	+	1	+	.	.	.	.	.	.	.	.	.	.	.	.	.	+	.	+	.	+	.	.	.	.	**11**
*Glaucium flavum* Crantz	.	.	.	.	.	.	.	.	.	.	.	.	.	.	.	+	+	.	.	.	.	.	.	+	.	+	.	.	.	.	.	.	.	1	+	+	+	**8**
*Senecio coronopifolius* Desf.	+	.	.	.	1	+	+	.	.	.	.	+	.	.	.	.	.	.	.	.	.	.	.	.	.	.	.	+	+	+	.	.	.	.	.	.	.	**8**
*Lobularia maritima* (L.) Desv.	+	+	.	.	+	+	+	+	.	.	.	.	.	.	.	.	.	.	.	.	.	.	+	.	.	.	.	.	.	.	.	.	.	.	.	.	.	**7**
*Matthiola tricuspidata* (L.) R.Br.	.	.	.	.	.	.	.	2	.	.	.	+	+	+	.	.	.	.	.	.	.	.	.	.	.	.	.	.	.	1	.	.	.	.	.	.	.	**5**
*Mercurialis annua* L.	+	.	.	.	+	+	.	.	.	.	.	.	.	.	.	.	.	.	.	.	.	.	.	.	.	.	.	1	+	.	.	.	.	.	.	.	.	**5**
*Salsola tragus* L.	.	.	.	.	.	.	.	.	.	.	.	.	.	.	.	.	.	2	.	.	.	.	.	.	.	.	.	.	.	.	1	+	.	.	.	1	+	**5**
*Cutandia maritima* (L.) Benth. ex Barbey	.	.	.	+	.	.	.	.	.	.	.	.	.	.	.	.	.	+	.	.	.	.	.	.	.	.	.	.	.	1	.	.	.	.	.	.	.	**3**
*Hedypnois rhagadioloides* (L.) F.W.Schmidt	.	.	.	.	.	+	.	.	.	.	+	+	.	.	.	.	.	.	.	.	.	.	.	.	.	.	.	.	.	.	.	.	.	.	.	.	.	**3**
*Medicago littoralis* Rohde ex Loisel.	.	.	.	.	.	.	.	.	+	+	.	+	.	.	.	.	.	.	.	.	.	.	.	.	.	.	.	.	.	.	.	.	.	.	.	.	.	**3**
*Polycarpon tetraphyllum* (L.) L. subsp. *alsinifolium* (Biv.) Ball.	.	.	.	.	.	.	.	.	.	.	1	3	1	.	.	.	.	.	.	.	.	.	.	.	.	.	.	.	.	.	.	.	.	.	.	.	.	**3**
*Polycarpon tetraphyllum* (L.) L. subsp. *diphyllum* (Cav.) O. Bolòs & Font Quer	.	+	+	.	.	.	.	.	.	.	.	.	.	.	.	.	.	.	.	.	.	.	.	.	.	.	.	.	+	.	.	.	.	.	.	.	.	**3**
*Poterium spinosum* L.	.	.	.	.	.	.	1	.	.	.	.	.	.	.	.	.	.	.	.	.	.	.	.	.	.	1	1	.	.	.	.	.	.	.	.	.	.	**3**
*Rumex bucephalophorus* L. subsp. *bucephalophorus*	1	.	.	.	.	+	.	.	.	.	.	.	.	.	.	.	.	.	.	.	.	.	.	+	.	.	.	.	.	.	.	.	.	.	.	.	.	**3**
*Sonchus oleraceus* L.	.	.	.	.	.	.	.	+	.	.	+	.	.	.	.	.	.	.	.	.	.	.	.	.	.	.	.	+	.	.	.	.	.	.	.	.	.	**3**
*Asparagus acutifolius* L.	.	.	.	.	.	.	.	.	.	.	.	.	.	.	.	.	.	.	.	.	.	.	+	.	.	.	+	.	.	.	.	.	.	.	.	.	.	**2**
*Calendula arvensis* (Vaill.) L.	+	.	.	.	.	+	.	.	.	.	.	.	.	.	.	.	.	.	.	.	.	.	.	.	.	.	.	.	.	.	.	.	.	.	.	.	.	**2**
*Hyoseris scabra* L.	+	.	.	.	.	+	.	.	.	.	.	.	.	.	.	.	.	.	.	.	.	.	.	.	.	.	.	.	.	.	.	.	.	.	.	.	.	**2**
*Lagurus ovatus* L. subsp. *ovatus*	.	.	.	.	.	+	+	.	.	.	.	.	.	.	.	.	.	.	.	.	.	.	.	.	.	.	.	.	.	.	.	.	.	.	.	.	.	**2**
*Ononis variegata* L.	.	.	.	.	.	.	.	.	.	.	.	+	.	.	.	.	.	+	.	.	.	.	.	.	.	.	.	.	.	.	.	.	.	.	.	.	.	**2**
*Polypogon monspeliensis* (L.) Desf.	.	.	.	.	.	.	.	.	.	.	.	.	.	.	.	.	.	.	.	.	.	.	.	.	.	.	.	.	.	.	.	1	.	+	.	.	.	**2**
*Ajuga iva* (L.) Schreb. subsp. *iva*	+	.	.	.	.	.	.	.	.	.	.	.	.	.	.	.	.	.	.	.	.	.	.	.	.	.	.	.	.	.	.	.	.	.	.	.	.	**1**
*Bromus hordeaceus* L. subsp. *hordeaceus*	.	.	.	.	.	.	.	.	1	.	.	.	.	.	.	.	.	.	.	.	.	.	.	.	.	.	.	.	.	.	.	.	.	.	.	.	.	**1**
*Cachrys libanotis* L.	.	.	.	.	.	.	.	.	.	.	.	.	.	.	.	.	.	.	.	.	.	.	.	.	.	.	2	.	.	.	.	.	.	.	.	.	.	**1**
*Cachrys pungens* Jan ex Guss.	.	.	.	.	.	.	.	.	.	.	.	.	.	.	.	.	.	.	.	.	.	.	+	.	.	.	.	.	.	.	.	.	.	.	.	.	.	**1**
*Cachrys sicula* L.	.	.	.	.	.	.	.	.	.	.	.	.	.	.	.	.	.	.	.	.	.	.	.	.	.	.	+	.	.	.	.	.	.	.	.	.	.	**1**
*Campanula erinus* L.	+	.	.	.	.	.	.	.	.	.	.	.	.	.	.	.	.	.	.	.	.	.	.	.	.	.	.	.	.	.	.	.	.	.	.	.	.	**1**
*Catapodium rigidum* (L.) C.E.Hubb. subsp. *rigidum*	+	.	.	.	.	.	.	.	.	.	.	.	.	.	.	.	.	.	.	.	.	.	.	.	.	.	.	.	.	.	.	.	.	.	.	.	.	**1**
*Cynoglossum creticum* Mill.	.	.	.	.	.	+	.	.	.	.	.	.	.	.	.	.	.	.	.	.	.	.	.	.	.	.	.	.	.	.	.	.	.	.	.	.	.	**1**
*Daucus carota* L. subsp. *carota*	.	.	.	.	.	.	.	+	.	.	.	.	.	.	.	.	.	.	.	.	.	.	.	.	.	.	.	.	.	.	.	.	.	.	.	.	.	**1**
*Ecbalium elaterium* (L.) A.Rich.	.	.	.	.	.	.	.	+	.	.	.	.	.	.	.	.	.	.	.	.	.	.	.	.	.	.	.	.	.	.	.	.	.	.	.	.	.	**1**
*Rumex spinosus* L.	.	.	.	.	.	r	.	.	.	.	.	.	.	.	.	.	.	.	.	.	.	.	.	.	.	.	.	.	.	.	.	.	.	.	.	.	.	**1**
*Festuca ciliata* Gouan	1	.	.	.	.	.	.	.	.	.	.	.	.	.	.	.	.	.	.	.	.	.	.	.	.	.	.	.	.	.	.	.	.	.	.	.	.	**1**
*Festuca fasciculata* Forssk.	.	.	.	+	.	.	.	.	.	.	.	.	.	.	.	.	.	.	.	.	.	.	.	.	.	.	.	.	.	.	.	.	.	.	.	.	.	**1**
*Hirschfeldia incana* (L.) Lagr.-Foss. subsp. *incana*	.	.	.	.	.	.	.	.	.	.	.	.	.	.	.	.	.	.	.	.	.	.	.	.	.	.	.	+	.	.	.	.	.	.	.	.	.	**1**
*Limonium sinuatum* (L.) Mill.	.	.	.	.	.	+	.	.	.	.	.	.	.	.	.	.	.	.	.	.	.	.	.	.	.	.	.	.	.	.	.	.	.	.	.	.	.	**1**
*Limonium virgatum* (Willd.) Fourr.	.	.	.	.	.	.	.	.	.	.	.	.	.	2	.	.	.	.	.	.	.	.	.	.	.	.	.	.	.	.	.	.	.	.	.	.	.	**1**
*Orobanche minor* Sm.	.	.	.	.	.	.	.	.	.	.	r	.	.	.	.	.	.	.	.	.	.	.	.	.	.	.	.	.	.	.	.	.	.	.	.	.	.	**1**
*Papaver rhoeas* L. subsp. *rhoeas*	.	.	.	.	.	.	.	.	.	.	.	.	.	r	.	.	.	.	.	.	.	.	.	.	.	.	.	.	.	.	.	.	.	.	.	.	.	**1**
*Pistacia lentiscus* L.	.	.	.	.	.	.	.	.	.	.	.	.	.	.	.	.	.	.	.	.	.	.	.	.	.	.	+	.	.	.	.	.	.	.	.	.	.	**1**
*Polygonum maritimum* L.	.	.	.	.	.	.	.	.	.	.	.	.	.	.	.	.	.	.	.	.	.	.	.	.	.	.	.	.	.	.	+	.	.	.	.	.	.	**1**
*Senecio pygmaeus* DC.	.	+	.	.	.	.	.	.	.	.	.	.	.	.	.	.	.	.	.	.	.	.	.	.	.	.	.	.	.	.	.	.	.	.	.	.	.	**1**
*Solanum nigrum* L.	.	.	.	.	.	.	.	.	.	.	.	.	.	.	.	.	.	.	.	.	.	.	.	.	+	.	.	.	.	.	.	.	.	.	.	.	.	**1**
*Stachys major* (L.) Bartolucci & Peruzzi	.	.	.	.	.	.	.	1	.	.	.	.	.	.	.	.	.	.	.	.	.	.	.	.	.	.	.	.	.	.	.	.	.	.	.	.	.	**1**
*Valantia muralis* L.	.	.	.	.	.	.	.	.	.	.	.	+	.	.	.	.	.	.	.	.	.	.	.	.	.	.	.	.	.	.	.	.	.	.	.	.	.	**1**
*Xanthium italicum* Moretti	.	.	.	.	.	.	.	.	.	.	.	.	.	.	.	.	.	.	.	.	.	.	.	.	.	.	.	.	.	.	+	.	.	.	.	.	.	**1**

**Table A5:** Phytosociological relevés from the Island of Capo Passero (SE Sicily)—rocky coast vegetation.

Cluster	8	8	8	8	8	8	8	8	8	8	8	8	8	8	8	8	8	8	8	8	8	9	9	9	9	9	9	9	9	9	9	9	9	10	10	10	10	10	10	10	10	10	10	10	10
Relevé number	61	89	128	133	151	152	153	154	92	192	193	194	149	150	95	96	98	114	97	104	103	88	90	155	156	93	94	106	108	129	132	130	131	91	101	117	119	169	170	110	115	116	102	109	105
Shrub layer (cover %)	-	-	65	80	85	85	85	90	-	10	10	20	70	80	55	55	45	35	45	70	45	-	-	90	90	-	-	-	10	65	85	90	90	40	60	30	15	60	70	55	25	55	60	40	30
Herbaceous layer (cover %)	45	70	-	-	-	-	-	-	15	-	-	-	-	-								85	95	-	-	65	85	70	55	-	-	-	-	-	-	-	-	-	-	-	-	-	-	-	-
Shrub layer—mean height (cm)	-	-	-	-	-	-	-	-	-	-	-	-	-	-	15	7	20	7	15	20	10	-	-	-	-	-	-	-	40	-	-	-	-	18	30	20	10	-	-	10	35	25	40	40	15
Herbaceous layer—mean height (cm)	10	8	-	-	-	-	-	-	15	-	-	-	-	-								10	6	-	-	15	15	5	10	-	-	-	-	-	-	-	-	-	-	-	-	-	-	-	-
Plot size (square m)	4	100	4	16	10	10	10	10	30	25	100	100	10	10	40	100	20	20	10	20	10	100	100	20	20	30	100	10	4	4	4	4	4	20	100	25	100	70	20	30	40	60	50	100	30
Exposure	-	-	-	-	-	-	-	-	-	-	-	-	-	-	-	-	-	SSE	-	-	-	-	NNW	-	-	-	-	NW	-	-	-	-	-	-	-	-	E	N	-	S	-	-	-	NNE	NW
Slope (°)	-	-	-	-	-	-	-	-	-	-	-	-	-	-	-	-	-	-	-	-	-	-	10	-	-	-	-	2	-	-	-	-	-	-	-	-	-	10	-	-	-	-	-	-	40
Stones and rocks (cover %)	30	35	-	-	-	-	-	-	40	-	-	-	-	-	40	40	35	70	30	35	40	-	15	-	-	20	35	35	70	-	-	-	-	65	60	75	80	-	-	85	80	85	80	95	95
**Char. Ass.**																																													
*Limonium hyblaeum* Brullo	.	.	.	.	.	.	.	.	.	1	1	+	.	.	.	.	.	.	.	.	.	.	.	.	.	.	.	.	.	.	.	.	.	.	.	.	.	.	.	.	.	.	.	+	.
*Limonium virgatum* (Willd.) Fourr.	.	2	2	2	1	1	2	+	2	1	1	1	3	3	1	2	2	1	1	2	1	+	1	.	.	.	+	+	1	.	.	.	.	.	+	1	1	+	1	+	1	2	+	2	1
*Mesembryanthemum nodiflorum* L.	.	2	2	.	+	+	+	+	1	.	+	+	.	.	1	3	+	2	+	.	.	4	5	3	4	2	4	3	2	4	2	2	1	.	.	.	.	+	+	.	.	.	.	.	+
*Suaeda vera* J.F.Gmel.	.	.	.	.	.	.	.	.	.	.	.	.	.	.	.	.	.	.	.	.	.	.	.	+	+	.	.	.	.	.	.	.	.	.	.	.	.	.	.	.	.	.	3	2	+
**Char. Crithmo-Staticetea**																																													
*Crithmum maritimum* L.	.	.	.	.	.	.	.	.	1	.	1	+	.	.	.	+	.	.	.	+	.	.	.	.	.	.	r	.	1	.	.	.	.	3	4	2	2	3	3	3	2	3	2	2	3
*Frankenia hirsuta* L.	2	2	2	.	+	+	1	+	+	1	1	1	1	+	1	1	+	+	+	+	+	+	1	+	1	3	2	3	2	.	.	.	.	2	+	.	.	.	+	+	.	+	+	1	1
*Lotus cytisoides* L.	.	.	1	.	.	.	.	.	.	.	.	.	.	.	.	.	.	+	1	1	+	.	.	.	.	.	.	+	+	.	.	.	.	.	1	.	.	.	.	+	1	1	3	+	1
*Cichorium spinosum* L.	.	.	.	.	.	.	.	+	.	.	.	+	.	.	.	.	.	.	.	.	.	.	.	.	.	.	.	.	.	.	.	.	.	.	.	.	.	.	.	3	2	1	.	.	.
*Spergularia heldreichii* E.Simon & P.Monnier	.	.	.	.	.	.	.	.	1	.	.	.	.	.	+	.	1	2	.	.	.	.	.	.	.	1	1	.	+	.	.	.	.	1	.	.	.	.	.	.	.	.	.	.	.
**Char. Salicornietea fruticosae**																																													
*Arthrocaulon meridionalis* Es.Ramírez, Rufo, Sánchez Mata, V.Fuente	.	.	+	.	.	.	.	.	.	.	1	.	.	.	.	+	1	+	r	.	.	.	.	.	.	.	.	.	1	.	.	.	.	.	.	1	1	.	+	.	.	.	.	1	.
*Aeluropus lagopoides* (L.) Trin. ex Thwaites	.	.	.	.	.	.	.	.	.	.	.	.	.	.	3	3	3	2	3	4	3	.	.	.	.	.	.	.	.	.	.	.	.	.	.	.	.	.	.	.	.	.	.	.	.
*Halimione portulacoides* (L.) Aellen	.	.	.	.	.	.	.	.	.	.	.	.	.	.	.	.	.	.	.	.	.	.	.	.	.	.	.	.	.	.	.	.	.	.	.	.	.	.	.	.	.	.	.	.	.
**Char. Saginetea maritimae**																																													
*Parapholis incurva* (L.) C.E.Hubb.	.	.	1	.	+	+	+	+	+	1	+	.	.	.	+	+	+	+	.	.	.	.	.	.	+	+	1	1	1	1	2	+	1	1	+	.	.	.	.	+	.	.	2	+	2
*Beta vulgaris* L. subsp. *maritima* (L.) Arcang.	.	.	.	.	.	.	.	.	+	.	.	.	.	.	1	+	.	1	1	1	1	1	1	.	.	1	2	1	1	3	2	3	3	+	+	.	+	.	.	+	.	.	1	+	1
*Catapodium balearicum* (Willk.) H.Scholz.	.	.	1	.	.	.	.	.	.	+	.	+	.	.	+	+	.	.	.	+	.	.	.	.	.	.	.	1	+	.	.	.	.	.	.	.	.	.	.	+	.	.	+	+	+
*Frankenia pulverulenta* L. subsp. *pulverulenta*	.	.	.	.	.	.	.	.	.	.	.	.	.	.	.	.	.	.	.	.	.	+	+	.	.	.	.	+	.	1	2	+	1	.	.	.	.	.	.	.	.	.	.	.	.
*Spergularia bocconei* (Scheele) Graebn.	.	.	.	.	.	.	.	.	.	+	+	.	.	.	.	.	.	.	.	.	.	.	.	.	.	.	.	.	.	1	+	3	1	.	.	.	.	.	.	.	.	.	.	.	.
*Spergularia marina* (L.) Besser	+	.	.	.	.	.	.	.	.	.	.	.	.	.	.	+	.	.	.	.	.	.	.	.	.	.	.	+	.	.	.	.	.	.	.	.	.	.	.	.	.	.	.	.	.
*Senecio pygmaeus* DC.	.	.	1	.	.	.	.	.	.	.	.	.	.	.	.	.	.	+	.	.	.	.	.	.	.	.	.	.	+	.	.	.	.	.	.	.	.	.	.	+	.	+	.	+	+
*Sagina maritima* Don	.	.	.	.	.	.	.	.	.	.	.	.	.	.	.	.	.	.	.	.	.	.	.	.	.	.	2	+	+	.	.	.	.	.	.	.		.	.	.	.	.	.	.	.
*Polypogon subspathaceus* Req.	.	.	.	.	.	.	.	.	.	.	.	.	.	.	.	.	.	.	.	.	.	.	.	.	.	.	+	.	.	.	.	.	.	.	.	.	.	.	.	.	.	.	.	.	.
**Other species**																																													
*Limonium sinuatum* (L.) Mill.	2	2	3	4	4	3	3	4	.	.	.	.	1	+	+	+	.	.	+	.	2	2	+	4	2	.	+	1	1	.	.	.	.	.	.	.	.	.	.	.	.	.	.	.	+
*Silene sedoides* Poir. subsp. *sedoides*	.	.	+	.	.	.	.	.	.	.	.	.	.	.	+	+	.	+	+	.	.	.	.	.	.	.	.	+	.	.	.	.	.	1	1	+	+	.	.	+	+	1	.	.	.
*Asteriscus aquaticus* (L.) Less.	+	.	.	1	.	.	.	.	.	.	.	.	.	.	.	.	.	+	.	.	.	+	.	+	1	.	r	+	.	.	.	.	.	.	.	.	.	.	.	.	.	.	.	.	+
*Plantago weldenii* Rchb.	.	.	.	.	.	.	.	.	+	.	.	.	.	.	+	.	.	+	.	+	+	.	.	.	.	.	.	.	.	.	.	.	.	.	.	.	.	.	.	.	.	.	1	.	.
*Reichardia picroides* (L.) Roth	.	.	.	.	.	.	.	.	.	.	.	.	.	.	.	.	.	+	+	.	.	.	.	.	.	.	.	+	.	.	.	.	.	.	+	.	.	.	.	.	.	.	+	.	+
*Sonchus oleraceus* L.	.	.	.	.	.	.	.	.	.	.	.	.	.	.	.	.	.	.	.	.	.	.	.	.	.	.	.	.	+	.	.	.	.	.	.	.	+	.	.	+	.	r	.	+	+
*Valantia muralis* L.	+	.	.	.	.	.	.	.	.	.	.	.	.	.	.	.	.	+	.	.	+	.	.	.	.	.	.	.	+	.	.	.	.	.	.	.	.	.	.	+	.	.	.	.	1
*Ecbalium elaterium* (L.) A.Rich.	.	.	.	.	.	.	.	+	.	.	.	.	.	.	.	.	.	.	.	.	.	1	.	.	+	.	.	.	.	.	.	.	.	.	.	.	.	.	.	1	.	.	.	.	.
*Arenaria leptoclados* (Rchb.) Guss. subsp. *leptoclados*	.	.	.	.	.	.	.	.	.	.	.	.	.	.	.	.	.	.	.	.	.	.	.	.	.	.	+	+	.	.	.	.	.	.	.	.	.	.	.	.	.	.	.	.	1
*Medicago littoralis* Rohde ex Loisel.	.	.	.	.	.	.	.	.	.	.	.	.	.	.	.	.	.	+	.	.	.	.	.	.	.	.	.	.	+	.	.	.	.	.	.	.	.	.	.	.	+	.	.	.	.
*Scolymus hispanicus* L. subsp. *hispanicus*	.	.	.	.	.	.	.	+	.	.	.	.	.	.	.	.	.	.	.	.	.	1	.	+	.	.	.	.	.	.	.	.	.	.	.	.	.	.	.	.	.	.	.	.	.
*Anthemis secundiramea* Biv.	.	.	.	.	.	.	.	.	.	.	.	.	.	.	+	.	.	.	.	.	.	.	.	.	.	.	.	.	.	.	.	.	.	.	.	.	.	.	.	.	.	+	.	.	.
*Chenopodium album* L. subsp. *album*	.	.	.	.	.	.	.	.	.	.	.	.	.	.	.	.	.	.	.	.	.	.	.	+	+	.	.	.	.	.	.	.	.	.	.	.	.	.	.	.	.	.	.	.	.
*Cuscuta planiflora* Ten.	.	.	.	.	.	.	.	.	.	.	.	.	.	.	.	.	.	.	.	.	.	.	.	.	.	.	.	.	.	.	.	.	.	.	.	.	.	.	.	+	.	.	.	.	+
*Echium arenarium* Guss.	.	.	.	.	.	.	.	.	.	.	.	.	.	.	.	.	.	.	.	.	.	+	.	.	.	.	.	.	.	.	.	.	.	.	.	.	+	.	.	.	.	.	.	.	.
*Euphorbia peplis* L.	.	.	.	.	.	.	.	.	.	.	.	.	.	.	.	.	.	+	.	.	.	.	.	.	.	.	.	.	.	.	.	.	.	.	.	.	.	.	.	+	.	.	.	.	.
*Hordeum murinum* L. subsp. *leporinum* (Link) Arcang.	.	.	.	.	.	.	.	.	.	.	.	.	.	.	.	.	.	+	.	.	.	.	.	.	.	.	.	.	.	.	.	.	.	.	.	.	.	.	.	+	.	.	.	.	.
*Rumex bucephalophorus* L. subsp. *bucephalophorus*	+	.	.	.	.	.	.	.	.	.	.	.	.	.	.	.	.	.	.	.	.	.	.	.	.	.	.	.	.	.	.	.	.	.	.	.	.	.	.	+	.	.	.	.	.
*Sedum rubens* L.	.	.	.	.	.	.	.	.	.	.	.	.	.	.	.	.	.	.	.	.	.	.	.	.	.	.	.	+	.	.	.	.	.	.	.	.	.	.	.	.	.	.	.	.	+
*Silene nicaensis* All.	.	.	+	.	.	.	.	.	.	.	.	.	.	.	.	.	.	.	.	.	.	.	.	.	.	.	.	.	.	.	.	.	.	.	.	.	.	.	.	.	.	.	.	.	.
*Anisantha fasciculata* (C. Presl) Nevski subsp. *fasciculata*	.	.	.	.	.	.	.	.	.	.	.	.	.	.	.	.	.	.	.	.	.	.	.	.	.	.	.	.	.	.	.	.	.	.	.	.	.	.	.	+	.	.	.	.	.
*Capparis orientalis* Veill.	.	.	.	.	.	.	.	.	.	.	.	.	.	.	.	.	.	.	.	.	.	.	.	.	.	.	.	.	.	.	.	.	.	.	.	.	.	+	.	.	.	.	.	.	.
*Catapodium pauciflorum* (Merino) Brullo, Giusso, Miniss. & Spamp.	.	.	+	.	.	.	.	.	.	.	.	.	.	.	.	.	.	.	.	.	.	.	.	.	.	.	.	.	.	.	.	.	.	.	.	.	.	.	.	.	.	.	.	.	.
*Centaurium tenuiflorum* (Hoffmanns. and Link) Fritsch subsp. *tenuiflorum*	.	.	.	.	.	.	.	.	.	.	.	.	.	.	.	.	.	.	.	.	.	.	.	.	.	.	+	.	.	.	.	.	.	.	.	.	.	.	.	.	.	.	.	.	.
*Erodium malacoides* (L.) L’Hér. subsp. *malacoides*	.	.	.	.	.	.	.	.	.	.	.	.	.	.	.	.	.	.	.	.	.	.	.	.	.	.	.	.	.	.	.	.	.	.	.	.	.	.	.	.	.	.	.	.	+
*Euphorbia terracina* L.	.	.	+	.	.	.	.	.	.	.	.	.	.	.	.	.	.	.	.	.	.	.	.	.	.	.	.	.	.	.	.	.	.	.	.	.	.	.	.	.	.	.	.	.	.
*Festuca fasciculata* Forssk.	.	.	.	.	.	.	.	.	.	.	.	.	.	.	.	.	.	.	.	.	.	.	.	.	.	.	.	.	.	.	.	.	.	.	.	.	.	.	.	+	.	.	.	.	.
*Hyoscyamus albus* L.	.	.	.	.	.	.	.	.	.	.	.	.	.	.	.	.	.	.	.	.	.	.	.	.	.	.	.	.	.	.	.	.	.	.	.	.	.	.	.	.	.	.	.	.	r
*Lobularia maritima* (L.) Desv.	.	.	.	.	.	.	.	.	.	.	.	.	.	.	.	.	.	.	.	.	.	+	.	.	.	.	.	.	.	.	.	.	.	.	.	.	.	.	.	.	.	.	.	.	.
*Lotus creticus* L.	.	.	.	.	.	.	.	.	.	.	.	.	.	.	.	.	.	+	.	.	.	.	.	.	.	.	.	.	.	.	.	.	.	.	.	.	.	.	.	.	.	.	.	.	.
*Lotus edulis* L.	.	.	.	.	.	.	.	.	.	.	.	.	.	.	.	.	.	.	.	.	.	.	.	.	.	.	.	+	.	.	.	.	.	.	.	.	.	.	.	.	.	.	.	.	.
*Lysimachia arvensis* (L.) U. Manns & Anderb. subsp. *arvensis*	.	.	.	.	.	.	.	.	.	.	.	.	.	.	.	.	.	.	.	.	.	.	.	.	.	.	.	.	.	.	.	.	.	.	.	.	.	.	.	+	.	.	.	.	.
*Mandragora autumnalis* Bertol.	.	.	.	.	.	.	.	.	.	.	.	.	.	.	.	.	.	.	.	.	.	1	.	.	.	.	.	.	.	.	.	.	.	.	.	.	.	.	.	.	.	.	.	.	.
*Mercurialis annua* L.	.	.	.	.	.	.	.	.	.	.	.	.	.	.	.	.	.	.	.	.	.	.	.	.	.	.	.	.	.	.	.	.	.	.	.	.	.	.	.	+	.	.	.	.	.
*Ononis natrix* L. subsp. *ramosissima* (Desf.) Batt.	.	.	.	.	.	.	.	.	.	.	.	.	.	.	.	.	.	.	.	.	.	.	.	.	.	.	.	.	.	.	.	.	.	.	.	.	+	.	.	.	.	.	.	.	.
*Orobanche minor* Sm.	.	.	.	.	.	.	.	.	.	.	.	.	.	.	.	.	.	.	.	.	.	.	.	.	.	.	.	.	.	.	.	.	.	.	.	.	r	.	.	.	.	.	.	.	.
*Pancratium maritimum* L.	.	.	+	.	.	.	.	.	.	.	.	.	.	.	.	.	.	.	.	.	.	.	.	.	.	.	.	.	.	.	.	.	.	.	.	.	.	.	.	.	.	.	.	.	.
*Senecio coronopifolius* Desf.	.	.	.	.	.	.	.	.	.	.	.	.	.	.	.	.	.	.	.	.	.	.	.	.	.	.	.	.	.	.	.	.	.	.	.	.	+	.	.	.	.	.	.	.	.
*Silene colorata* Poir,	.	.	.	.	.	.	.	.	.	.	.	.	.	.	.	.	.	.	.	.	.	.	.	.	.	.	.	.	.	.	.	.	.	.	.	.	+	.	.	.	.	.	.	.	.
*Sonchus bulbosus* (L.) N.Kilian & Greuter subsp. *bulbosus*	.	.	.	.	.	.	.	.	.	.	.	.	.	.	.	.	.	+	.	.	.	.	.	.	.	.	.	.	.	.	.	.	.	.	.	.	.	.	.	.	.	.	.	.	.
*Thapsia garganica* L. subsp. *garganica*	.	.	.	.	.	.	.	.	.	.	.	.	.	.	.	.	.	.	.	.	.	1	.	.	.	.	.	.	.	.	.	.	.	.	.	.	.	.	.	.	.	.	.	.	.

**Table A6:** Phytosociological relevés from the Island of Capo Passero (SE Sicily)—rocky coast vegetation.

Cluster	11	11	11	11	11	11	11	11	11	11	11	11	11	11	11	11	11	11	11	11	11	11	12	12	12	12	12	12	**Occurrences**
Relevé number	99	100	163	168	182	165	141	143	142	183	186	118	139	181	187	180	136	120	138	188	140	137	144	167	166	164	184	185	
Shrub layer (cover %)	30	30	80	75	80	75	80	80	80	85	65	45	70	50	70	60	50	40	60	75	60	60	70	60	60	70	85	90	
Herbaceous layer (cover %)	-	-	-	-	-	-	-	-	-	-	-	-	-	-	-	-	-	-	-	-	-	-	-	-	-	-	-	-	
Shrub layer—mean height (cm)	40	40	-	-	-	-	-	-	-	-	-	30	-	-	-	-	-	30	-	-	-	-	-	-	-	-	-	-	
Herbaceous layer—mean height (cm)	-	-	-	-	-	-	-	-	-	-	-	-	-	-	-	-	-	-	-	-	-	-	-	-	-	-	-	-	
Plot size (square m)	50	50	10	20	40	10	25	100	100	40	40	50	25	20	40	40	100	50	25	40	25	25	100	10	10	10	40	40	
Exposure	-	-	-	-	-	-	-	-	-	-	-	-	-	-	-	-	E	-	-	-	-	-	-	-	-	-	-	-	
Slope (°)	-	-	-	-	-	-	-	-	-	-	-	-	-	-	-	-	3	-	-	-	-	-	-	-	-	-	-	-	
Stones and rocks (cover %)	80	80	-	-	-	-	-	-	-	-	-	90	-	-	-	-	-	90	-	-	-	-	-	-	-	-	-	-	
**Char. Ass.**																													
*Limonium hyblaeum* Brullo	.	+	.	.	.	.	.	.	.	.	.	.	.	.	.	.	.	.	.	.	.	.	.	.	.	.	.	.	**5**
*Limonium virgatum* (Willd.) Fourr.	1	+	+	+	.	+	.	.	+	.	.	.	+	+	1	1	+	1	2	+	3	.	.	1	+	.	.	.	**53**
*Mesembryanthemum nodiflorum* L.	+	+	.	+	.	1	+	+	+	.	.	.	.	.	.	.	+	.	.	.	.	.	+	+	+	+	+	.	**42**
*Suaeda vera* J.F.Gmel.	1	1	1	1	1	1	2	1	1	2	1	.	.	.	.	.	.	.	.	.	.	.	3	3	3	3	4	4	**22**
**Char. Crithmo-Staticetea**																													
*Crithmum maritimum* L.	.	.	.	.	+	.	+	.	+	1	2	1	+	1	1	2	1	.	1	+	.	+	+	1	.	.	.	+	**36**
*Frankenia hirsuta* L.	+	+	1	+	+	1	.	.	.	.	.	.	.	.	.	.	+	.	.	.	.	.	.	+	+	+	.	.	**46**
*Lotus cytisoides* L.	.	.	.	.	.	.	.	.	.	.	.	1	.	.	.	.	.	.	.	.	.	.	.	.	.	.	.	.	**15**
*Cichorium spinosum* L.	.	.	.	.	.	.	.	.	.	.	.	.	.	.	.	.	.	.	.	.	.	.	.	.	.	.	.	.	**5**
*Spergularia heldreichii* E.Simon & P.Monnier	1	.	.	.	.	.	.	.	.	.	.	.	.	.	.	.	.	.	.	.	.	.	.	.	.	.	.	.	**9**
**Char. Salicornietea fruticosae**																													
*Arthrocaulon meridionalis* Es.Ramírez, Rufo, Sánchez Mata, V.Fuente	2	3	3	4	4	3	4	4	4	4	3	3	3	4	4	3	3	3	4	4	3	3	2	1	2	1	.	+	**38**
*Aeluropus lagopoides* (L.) Trin. ex Thwaites	.	.	.	.	.	1	+	+	+	.	.	.	.	.	.	.	.	.	.	.	.	.	1	1	+	.	.	.	**14**
*Halimione portulacoides* (L.) Aellen	.	.	.	.	.	.	.	.	.	.	.	.	.	.	.	.	.	1	+	+	1	1	.	.	+	.	.	.	**6**
**Char. Saginetea maritimae**																													
*Parapholis incurva* (L.) C.E.Hubb.	+	.	.	.	.	+	.	.	.	.	.	.	.	.	.	.	.	.	.	.	.	.	+	+	.	+	.	.	**32**
*Beta vulgaris* L. subsp. *maritima* (L.) Arcang.	+	+	.	.	.	.	+	+	+	+	+	.	.	.	.	.	.	.	.	.	.	.	+	.	.	.	+	+	**34**
*Catapodium balearicum* (Willk.) H.Scholz.	.	.	.	.	.	.	.	.	.	.	.	.	.	.	.	.	.	.	.	.	.	.	.	.	.	.	.	.	**12**
*Frankenia pulverulenta* L. subsp. *pulverulenta*	.	.	.	.	.	.	.	.	.	.	.	.	.	.	.	.	.	.	.	.	.	.	.	.	.	.	.	.	**7**
*Spergularia bocconei* (Scheele) Graebn.	.	.	.	.	.	.	.	.	.	.	.	.	.	.	.	.	+	.	.	.	.	.	.	.	.	.	.	.	**7**
*Spergularia marina* (L.) Besser	.	+	.	.	.	.	.	.	.	.	.	.	.	.	.	.	.	.	.	.	.	.	.	.	.	.	.	.	**4**
*Senecio pygmaeus* DC.	.	.	.	.	.	.	.	.	.	.	.	.	.	.	.	.	.	.	.	.	.	.	.	.	.	.	.	.	**7**
*Sagina maritima* Don	.	.	.	.	.	.	.	.	.	.	.	.	.	.	.	.	.	.	.	.	.	.	.	.	.	.	.	.	**4**
*Polypogon subspathaceus* Req.	.	.	.	.	.	.	.	.	.	.	.	.	.	.	.	.	.	.	.	.	.	.	.	.	.	.	.	.	**1**
**Other species**																													
*Limonium sinuatum* (L.) Mill.	.	.	.	.	.	.	.	.	.	.	.	.	.	.	.	.	.	.	.	.	.	1	1	+	+	.	.	.	**26**
*Silene sedoides* Poir. subsp. *sedoides*	+	1	.	.	.	.	.	.	.	.	.	.	.	.	.	.	.	.	.	.	.	.	.	.	.	.	.	.	**15**
*Asteriscus aquaticus* (L.) Less.	.	.	.	.	.	.	.	.	.	.	.	.	.	.	.	.	.	.	.	.	.	.	.	.	.	.	.	.	**9**
*Plantago weldenii* Rchb.	.	.	.	.	.	.	.	.	.	.	.	.	.	.	.	.	.	.	.	.	.	.	.	.	.	.	.	.	**6**
*Reichardia picroides* (L.) Roth	.	.	.	.	.	.	.	.	.	.	.	.	.	.	.	.	.	.	.	.	.	.	.	.	.	.	.	.	**6**
*Sonchus oleraceus* L.	.	.	.	.	.	.	.	.	.	.	.	.	.	.	.	.	.	.	.	.	.	.	.	.	.	.	.	.	**6**
*Valantia muralis* L.	.	.	.	.	.	.	.	.	.	.	.	.	.	.	.	.	.	.	.	.	.	.	.	.	.	.	.	.	**6**
*Ecbalium elaterium* (L.) A.Rich.	.	.	.	.	.	.	.	.	.	.	.	.	.	.	.	.	.	.	.	.	.	.	.	.	.	.	.	.	**4**
*Arenaria leptoclados* (Rchb.) Guss. subsp. *leptoclados*	.	.	.	.	.	.	.	.	.	.	.	.	.	.	.	.	.	.	.	.	.	.	.	.	.	.	.	.	**3**
*Medicago littoralis* Rohde ex Loisel.	.	.	.	.	.	.	.	.	.	.	.	.	.	.	.	.	.	.	.	.	.	.	.	.	.	.	.	.	**3**
*Scolymus hispanicus* L. subsp. *hispanicus*	.	.	.	.	.	.	.	.	.	.	.	.	.	.	.	.	.	.	.	.	.	.	.	.	.	.	.	.	**3**
*Anthemis secundiramea* Biv.	.	.	.	.	.	.	.	.	.	.	.	.	.	.	.	.	.	.	.	.	.	.	.	.	.	.	.	.	**2**
*Chenopodium album* L. subsp. *album*	.	.	.	.	.	.	.	.	.	.	.	.	.	.	.	.	.	.	.	.	.	.	.	.	.	.	.	.	**2**
*Cuscuta planiflora* Ten.	.	.	.	.	.	.	.	.	.	.	.	.	.	.	.	.	.	.	.	.	.	.	.	.	.	.	.	.	**2**
*Echium arenarium* Guss.	.	.	.	.	.	.	.	.	.	.	.	.	.	.	.	.	.	.	.	.	.	.	.	.	.	.	.	.	**2**
*Euphorbia peplis* L.	.	.	.	.	.	.	.	.	.	.	.	.	.	.	.	.	.	.	.	.	.	.	.	.	.	.	.	.	**2**
*Hordeum murinum* L. subsp. *leporinum* (Link) Arcang.	.	.	.	.	.	.	.	.	.	.	.	.	.	.	.	.	.	.	.	.	.	.	.	.	.	.	.	.	**2**
*Rumex bucephalophorus* L. subsp. *bucephalophorus*	.	.	.	.	.	.	.	.	.	.	.	.	.	.	.	.	.	.	.	.	.	.	.	.	.	.	.	.	**2**
*Sedum rubens* L.	.	.	.	.	.	.	.	.	.	.	.	.	.	.	.	.	.	.	.	.	.	.	.	.	.	.	.	.	**2**
*Silene nicaensis* All.	.	.	.	.	.	.	.	.	.	.	.	.	.	.	.	.	1	.	.	.	.	.	.	.	.	.	.	.	**2**
*Anisantha fasciculata* (C. Presl) Nevski subsp. *fasciculata*	.	.	.	.	.	.	.	.	.	.	.	.	.	.	.	.	.	.	.	.	.	.	.	.	.	.	.	.	**1**
*Capparis orientalis* Veill.	.	.	.	.	.	.	.	.	.	.	.	.	.	.	.	.	.	.	.	.	.	.	.	.	.	.	.	.	**1**
*Catapodium pauciflorum* (Merino) Brullo, Giusso, Miniss. & Spamp.	.	.	.	.	.	.	.	.	.	.	.	.	.	.	.	.	.	.	.	.	.	.	.	.	.	.	.	.	**1**
*Centaurium tenuiflorum* (Hoffmanns. and Link) Fritsch subsp. *tenuiflorum*	.	.	.	.	.	.	.	.	.	.	.	.	.	.	.	.	.	.	.	.	.	.	.	.	.	.	.	.	**1**
*Erodium malacoides* (L.) L’Hér. subsp. *malacoides*	.	.	.	.	.	.	.	.	.	.	.	.	.	.	.	.	.	.	.	.	.	.	.	.	.	.	.	.	**1**
*Euphorbia terracina* L.	.	.	.	.	.	.	.	.	.	.	.	.	.	.	.	.	.	.	.	.	.	.	.	.	.	.	.	.	**1**
*Festuca fasciculata* Forssk.	.	.	.	.	.	.	.	.	.	.	.	.	.	.	.	.	.	.	.	.	.	.	.	.	.	.	.	.	**1**
*Hyoscyamus albus* L.	.	.	.	.	.	.	.	.	.	.	.	.	.	.	.	.	.	.	.	.	.	.	.	.	.	.	.	.	**1**
*Lobularia maritima* (L.) Desv.	.	.	.	.	.	.	.	.	.	.	.	.	.	.	.	.	.	.	.	.	.	.	.	.	.	.	.	.	**1**
*Lotus creticus* L.	.	.	.	.	.	.	.	.	.	.	.	.	.	.	.	.	.	.	.	.	.	.	.	.	.	.	.	.	**1**
*Lotus edulis* L.	.	.	.	.	.	.	.	.	.	.	.	.	.	.	.	.	.	.	.	.	.	.	.	.	.	.	.	.	**1**
*Lysimachia arvensis* (L.) U. Manns & Anderb. subsp. *arvensis*	.	.	.	.	.	.	.	.	.	.	.	.	.	.	.	.	.	.	.	.	.	.	.	.	.	.	.	.	**1**
*Mandragora autumnalis* Bertol.	.	.	.	.	.	.	.	.	.	.	.	.	.	.	.	.	.	.	.	.	.	.	.	.	.	.	.	.	**1**
*Mercurialis annua* L.	.	.	.	.	.	.	.	.	.	.	.	.	.	.	.	.	.	.	.	.	.	.	.	.	.	.	.	.	**1**
*Ononis natrix* L. subsp. *ramosissima* (Desf.) Batt.	.	.	.	.	.	.	.	.	.	.	.	.	.	.	.	.	.	.	.	.	.	.	.	.	.	.	.	.	**1**
*Orobanche minor* Sm.	.	.	.	.	.	.	.	.	.	.	.	.	.	.	.	.	.	.	.	.	.	.	.	.	.	.	.	.	**1**
*Pancratium maritimum* L.	.	.	.	.	.	.	.	.	.	.	.	.	.	.	.	.	.	.	.	.	.	.	.	.	.	.	.	.	**1**
*Senecio coronopifolius* Desf.	.	.	.	.	.	.	.	.	.	.	.	.	.	.	.	.	.	.	.	.	.	.	.	.	.	.	.	.	**1**
*Silene colorata* Poir,	.	.	.	.	.	.	.	.	.	.	.	.	.	.	.	.	.	.	.	.	.	.	.	.	.	.	.	.	**1**
*Sonchus bulbosus* (L.) N.Kilian & Greuter subsp. *bulbosus*	.	.	.	.	.	.	.	.	.	.	.	.	.	.	.	.	.	.	.	.	.	.	.	.	.	.	.	.	**1**
*Thapsia garganica* L. subsp. *garganica*	.	.	.	.	.	.	.	.	.	.	.	.	.	.	.	.	.	.	.	.	.	.	.	.	.	.	.	.	**1**

**Dates of relevés**

Rel. 6, 7, 18, 21, 46, 50, 43, 47, 52, 114, 117, 105 (04 April 1998, Cristaudo); Rel. 8, 9, 10, 12, 16, 24, 29, 42, 53, 59, 55, 78, 100 (16 May 1998, Cristaudo); Rel. 11, 30, 35, 38, 44, 54, 73, 83, 87, 95 (10 April 1999, Cristaudo); Rel. 23, 26, 36, 37, 56, 88, 103, 106, 109, 110, 111, 115 (11 April 1999, Cristaudo); Rel. 4, 14, 31, 33, 39, 68, 69, 71, 72, 74, 84, 85 (08 May 1999, Cristaudo); Rel. 63, 65 (09 May 1999, Cristaudo); Rel. 5, 17, 34, 99, 108, 113 (23 October 1999, Cristaudo); Rel. 1, 3, 15, 20, 27, 48, 49, 118, 119 (01 April 2000, Cristaudo); Rel. 2, 13, 19, 25, 28, 40, 45, 41, 51, 98, 101, 102, 104 120, 121 (26 April 2000, Cristaudo); Rel. 32, 79, 81, 90, 93, 97, 107, 112 (20 May 2000, Cristaudo); Rel. 22, 57, 58, 60, 61, 62, 64, 66, 67, 70, 75, 76, 77, 80, 82, 86, 89, 91, 94, 96, 116 (04 June 2000, Cristaudo);
Rel. 122–148 (17 April 2019, Sciandrello); Rel. 149–170 (29 June 2019, Sciandrello); Rel. 171–172 (20 April 2006, Minissale, Sciandrello, Spampinato); Rel. 173–191 (03 February 2017, Sciandrello); Rel. 192–202 (Pirola 1959).
